# Finite-state parameter space maps for pruning partitions in modularity-based community detection

**DOI:** 10.1038/s41598-022-20142-6

**Published:** 2022-09-23

**Authors:** Ryan A. Gibson, Peter J. Mucha

**Affiliations:** 1 Department of Mathematics, University of North Carolina, Chapel Hill, NC 27599-3250 USA; 2 Department of Computer Science, University of North Carolina, Chapel Hill, NC 27599-3175 USA; 3 Department of Applied Physical Sciences, University of North Carolina, Chapel Hill, NC 27599-3050 USA; 4 Department of Mathematics, Dartmouth College, Hanover, NH 03755-3551 USA

**Keywords:** Applied mathematics, Statistical physics

## Abstract

Partitioning networks into communities of densely connected nodes is an important tool used widely across different applications, with numerous methods and software packages available for community detection. Modularity-based methods require parameters to be selected (or assume defaults) to control the resolution and, in multilayer networks, interlayer coupling. Meanwhile, most useful algorithms are heuristics yielding different near-optimal results upon repeated runs (even at the same parameters). To address these difficulties, we combine recent developments into a simple-to-use framework for pruning a set of partitions to a subset that are self-consistent by an equivalence with the objective function for inference of a degree-corrected planted partition stochastic block model (SBM). Importantly, this combined framework reduces some of the problems associated with the stochasticity that is inherent in the use of heuristics for optimizing modularity. In our examples, the pruning typically highlights only a small number of partitions that are fixed points of the corresponding map on the set of somewhere-optimal partitions in the parameter space. We also derive resolution parameter upper bounds for fitting a constrained SBM of *K* blocks and demonstrate that these bounds hold in practice, further guiding parameter space regions to consider. With publicly available code (http://github.com/ragibson/ModularityPruning), our pruning procedure provides a new baseline for using modularity-based community detection in practice.

## Introduction

Many real-world data sets can be naturally encoded as networks in which the objects of interest and their relationships are represented by nodes and edges, respectively. Network analysis has proved to be a powerful tool across applications in biology, computer science, sociology, neuroscience, and many other fields. Community detection (also known as graph partitioning and network clustering) is a particularly popular technique^1–4^. While the interpretation of different community structures is typically domain-specific, the existence of communities and their memberships are often of significant interest. In social networks, communities may demarcate the limits of social cliques or groups. In biological networks encoding gene-protein relationships, clusters can reveal information about pathways and processes. In technological networks, the hierarchical structure of communities can be used to compress data and to detect abnormalities. In computer science, many standard problems, such as scheduling work across clusters, can be naturally reduced to graph partitioning. Though a single definition of “community” has never been widely accepted (see, e.g., Peel et al.^5^ and Priebe et al.^6^), many of the different definitions of communities lead to formulations that identify groups of nodes that are more densely connected internally within the communities than to the rest of the network, in line with other notions of unsupervised clustering of data.

One of the most popular methods for community detection is to maximize a quantity known as modularity, which measures the total weight of edges within communities relative to that expected under an appropriate random graph model. Modularity was first introduced by Newman and Girvan^7^ for undirected networks and later extended to a variety of other settings. Modularity optimization has a number of well-known limitations that makes it problematic as a method of community detection: it is a descriptive measure without any underlying statistical or generative principle, it only finds assortative structures, it is biased towards balanced communities of similar sizes, particularly when the null model does not describe the network well (including but not limited to when the community sizes vary drastically), and the global optimization is NP-hard^8^. Indeed, we encourage readers to consult the extensive discussion by Peixoto^9^. Nevertheless, modularity remains one of the most popular methods for community detection in real-world networks, in part because a number of fast heuristics are readily available across multiple computational platforms. Perhaps most notably, the Louvain^10^ algorithm is very widely used because of its apparent balance between speed and the quality of the results, while the newer Leiden^11^ algorithm promises further improvement. At the same time, multilayer modularity^12^ is one of a relatively small handful of community detection methods available for multilayer networks^13^, a general framework in which a collection of interrelated networks are treated as individual “layers” in a larger, connected data structure. These are appropriate for handling networks with multiple kinds of connections (multiplex), networks that vary over time, networks of networks, and other structures. The generalized formulations of modularity include a resolution parameter^14^ that can be used to control the number and sizes of communities found at the corresponding maximum. But the possibility of multiple important scales of communities across different resolution parameters, the need to consider different interlayer coupling values in multilayer networks, and the run-to-run variation in communities from heuristic algorithms lead to serious challenges reconciling results. In practice, users must typically reconcile multiple partitions of nodes into communities while exploring the parameter space, if they even realize the need to address these issues (and it would seem many do not).

Our method introduced here aims to make it easier for community detection users who are already employing modularity to use it better, to avoid at least some of these pitfalls. To this end, we combine two previously disconnected advances from the recent literature: the Convex Hull of Admissible Modularity Partitions (CHAMP) algorithm^15,16^ for post-processing a set of partitions, and the equivalence between the objective functions for modularity and for inference of degree-corrected planted partitions recently identified by Newman^17^ and Pamfil et al.^18^. We briefly introduce each of these prior works next (see also “Methods” and the detailed discussion of each of these prior methods in Sections A–C of the Supplementary Information [SI]). We then combine these methods and demonstrate our results from using them together to define a simpler map.

The CHAMP algorithm^15,16^ provides a framework for quickly post-processing a provided set of partitions of nodes into communities, identifying the “admissible” subset of partitions that have non-empty domains of optimality in the resolution/coupling parameter space (relative to the provided set of partitions). The CHAMP approach is highly flexible in that it post-processes a set of partitions to identify the best subset (in the modularity sense), however many different partitions are provided and independent of the possibly multiple resolution parameter values or even different community detection methods that were used to obtain these partitions in the first place. Moreover, CHAMP does not prescribe one way to handle the admissible subset of partitions that are somewhere-optimal candidates, allowing users to make decisions based on the number of partitions in the admissible subset and the sizes and shapes of the associated domains of optimality. Of course, because CHAMP post-processes sets of community labels found by other means, the overall quality of the results obtained can depend strongly on the number and quality of the partitions that are input into CHAMP. However, and importantly, the added computational cost of CHAMP is trivial compared to that typically used to obtain partitions of nodes into communities in the first place. In these senses, applying CHAMP can only improve one’s perspective on how to handle disparate partitions of nodes into communities obtained for a given data set.

Another popular method for detecting communities is to fit a generative model known as a “stochastic block model” (SBM) to the network (see, e.g., Karrer & Newman^19^, Peixoto^20^, and Funke & Becker^21^). Importantly, SBMs are statistically principled and can consider more general block structures than the “assortative” structures explicitly sought in modularity maximization (though we will only consider assortative community structure here). While the descriptive nature of modularity and the generative approach of SBMs would at first appear to have little in common, Newman^17^ demonstrated an equivalence between the objective functions for modularity maximization and statistical inference for a particular degree-corrected planted partition SBM. Newman then used this equivalence to define an iterative procedure to obtain the modularity resolution parameter, $\gamma$, corresponding to a selected number of communities in a network (see “Methods” and Section A of the Supplementary Information [SI]). Pamfil et al.^18^ generalized Newman’s equivalence to multilayer networks of various types and extended the iterative procedure to obtain both $\gamma$ and the interlayer coupling $\omega$ (see “Methods” and SI Section B). Importantly, in so doing they also demonstrated conditions under which the iterative procedure leads to unstable fixed points.

**Figure 1:**
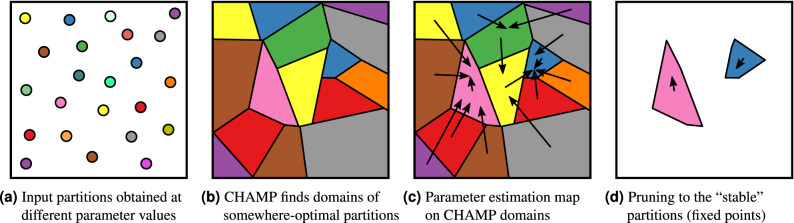
Visualization of our method. (**a**) Input partitions are obtained, usually through modularity maximization at various points across the resolution/coupling parameter space. (**b**) We use CHAMP^15,16^ to find the partitions’ domains of optimality (relative to the input set) within the space, discarding partitions that are nowhere optimal. (**c**) For each remaining partition, we use the objective function equivalence^17,18^ to estimate the “correct” parameter point, depicted here by arrows from each partition’s domain to its parameter estimate. (**d**) The “stable” partitions are those whose parameters fall within their domains of optimality; that is, they are fixed points of the map. By combining these methods, the resulting map is deterministic (conditional on the set of partitions input to CHAMP) on a finite set of states, so all fixed points are inherently stable.

As depicted in Fig. 1, our method combines the iterative parameter estimation from modularity-SBM equivalence^17,18^ with the post-processing of CHAMP^15,16^ to resolve issues that arise from the heuristic nature of modularity maximization. Bringing together these different elements, we provide a complete methodology for exploring a 2D $(\gamma ,\omega )$ parameter space in modularity-based community detection in multilayer networks. Running CHAMP on a set of input partitions, however obtained, identifies the finite subset of admissible partitions that are somewhere optimal (relative to the input set) and their associated domains of optimality. We then compute the estimated parameter point for each partition, per Newman^17^ for (single-layer) networks or the appropriate Pamfil et al.^18^ model for multilayer networks. Combining these previously disconnected approaches, we identify which domain of optimality that estimated parameter point resides in, and its corresponding partition, thus mapping each partition in the admissible CHAMP subset to a member of the same subset. That is, given a set of input community partitions, however obtained, this synthesized approach defines a deterministic map on the subset of admissible partitions. The fixed points of this finite-state map are the “stable” partitions that yield the highest modularity (from the input set) at their associated “correct” parameters in the corresponding SBM equivalence. Importantly, because this deterministic map defined on a given CHAMP subset includes only a finite number of possible states (partitions in the admissible subset), any fixed point of the map is inherently stable.

## Results

We demonstrate the advantages of this combined approach on (1) a well-studied, small (single-layer) network, (2) a synthetic hierarchical community model, (3) a synthetic multilayer network, and (4) a real-world multilayer network. In these demonstrations, we emphasize the differences between pre-specifying a fixed number of communities *K*, as in Newman’s iterative approach^17^, versus allowing *K* to be determined by the modularity maximization heuristic, as in Pamfil et al.^18^ (see also SI:B.5 about allowing *K* to vary). To further guide parameter selection in practice, we also derive an upper bound $\gamma _\text {max}$ for the estimated resolution parameter for *K* equal-sized communities. Additional details appear in the Supplementary Information (SI), including details about the previously-known objective function equivalencies^17,18^ (SI:A–B, in a common notation), discussion of the previously-developed CHAMP method^15,16^ (SI:C), further results on our demonstration examples (SI:D–G), additional examples (SI:H–I), full derivation of $\gamma _\text {max}(K)$ (SI:J), and a practical discussion about performance and the possibility of periodic orbits (SI:K). The code and data used to generate our results are available at http://github.com/ragibson/ModularityPruning.

### Zachary karate club

 We start with the (unweighted) Zachary karate club network^22^, one of the most popular examples of community structure in the network science literature. This network describes the social relationships between individuals in a university karate club shortly before a disagreement split the group in two. The Zachary karate club is so well studied (see the Zachary Karate Club Club^23^) that one might accuse us of using a gratuitous example; but even this simple example demonstrates the value of our approach.

**Figure 2:**
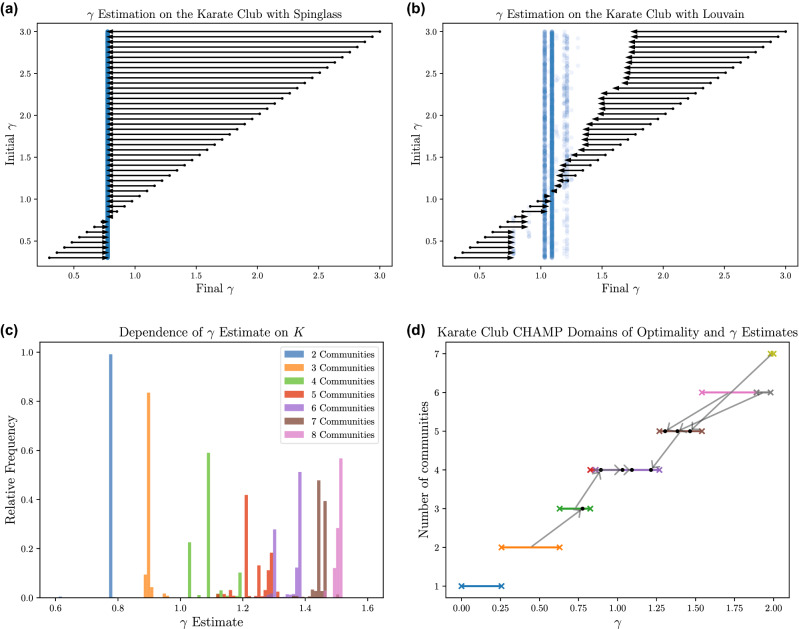
Iterative steps to determine “correct” values for the resolution parameter $\gamma$ on the Zachary karate club. Behavior observed using individual partitions obtained at each $\gamma$ by the (**a**) spin glass algorithm^14^ as implemented in igraph^24^, restricted to finding $K=2$ communities and the (**b**) Louvain algorithm^10^, which does not fix *K*, as implemented by Traag^25^. Arrows show average movement (over 100 trials) in $\gamma$ with the base of the tail at the initial $\gamma$ used and the head of the arrow indicating the (averaged) $\gamma$ estimate. Blue points indicate paired initial and final $\gamma$ values obtained after multiple steps (from multiple runs at each $\gamma$). (**c**) Frequencies of $\gamma$ estimates from 1e7 runs of Louvain on a uniform $\gamma \in [0,2]$ grid, relative to observed partitions with the same number of communities *K*. (**d**) Domains of optimality and associated $\gamma$ estimates for the 9 partitions of the Zachary karate club admitted by CHAMP starting from the 1e7 Louvain calls on $\gamma \in [0,2]$ mentioned above. Each of these 9 partitions is indicated by a horizontal line segment bounded by ‘x’ symbols at height indicating the number of communities. The “correct” value of $\gamma$ for each of these partitions (except the trivial 1-community partition, which has no such estimate) is indicated by an arrow from the midpoint of the partition to a corresponding dot, possibly within another domain. Notably, only one of these partitions (with $K=4$) yields a fixed point under the map, with its arrow pointing inside its domain.

The behavior of $\gamma$ estimate iterations using two different modularity maximization algorithms is shown in Fig. 2. Whereas the Louvain algorithm^10^ does not restrict the number of communities *K* (similar to Pamfil et al.’s^18^ use of GenLouvain^26^), the spin glass algorithm^14^ is run here with $K=2$ to compare directly with Newman’s results with fixed $K=2$^17^. (In such a small network, one might instead add a resolution parameter to a method like community_optimal_modularity in igraph, which recasts modularity maximization into an integer programming problem to guarantee the global optimum; but for the purposes of the present example we prefer to employ heuristics like those used for larger networks, to demonstrate the possible behaviors.) Matching Newman’s iterative scheme, which consistently converged to an optimal estimate $\gamma \approx 0.78$ for a 2-community partition closely matching the true split, the scheme using the spin glass algorithm converges to this $\gamma$ after a single iteration, regardless of the initial $\gamma$ (see Fig. 2a).

In contrast, the Pamfil et al. approach does not keep *K* fixed during the iterations^18^ (see SI:B.5 for discussion of some consequences of allowing *K* to vary). Applied to this simple single-layer example, we observe greater stochasticity when allowing *K* to vary within the iterative scheme’s use of Louvain (see Fig. 2b). The iterations most frequently converge to estimates with $1.0< \gamma < 1.1$, corresponding to different 4-community partitions. However, the scheme can also converge to the 2-community partition with $\gamma \approx 0.78$ if the iterative procedure is initialized with a small $\gamma$ value and if in the employed stopping condition the follow-up Louvain calls fail to identify the higher-quality 3-community partition at that $\gamma$. That 3-community partition then provides a $\gamma \approx 0.9$ estimate, and as seen in Fig. 2b it is possible to stop at that result; but there is a 4-community partition with higher quality there, and so the scheme frequently proceeds to a 4-community partition with $1.0< \gamma < 1.1$. As expected, there is a strong dependence between a partition’s $\gamma$ estimate and its number of communities, as seen in Fig. 2c since increasing $\gamma$ typically promotes a larger number of smaller communities. Notably, the difference between the exceptionally stable $K=2$ fixed point and the stochastic fluctuations when allowing *K* to vary are striking, especially for such a small, simple network. In hindsight, these fluctuations are perhaps less surprising when one considers that the duality between modularity optimization and SBM inference depends on estimates of the SBM parameters, which may differ greatly when the number of blocks is changed as allowed by Pamfil et al. (but not Newman). Hence, in general, the results of resolution parameter estimation can strongly depend on the number of communities returned by the modularity maximization heuristic of choice.

The stochastic nature of the above results are greatly suppressed in our combined approach depicted in Fig. 1 that includes CHAMP. Indeed, given an identified set of input partitions, the iterative scheme becomes a finite-state deterministic map on the admissible subset of those partitions, from which we can trivially identify the fixed points without any further problems of randomness or stability. Of course, we pay for this simplicity by finding a set of input partitions to prune in the first place. For a small network like the Zachary karate club, we can very reasonably generate an excessive number of input partitions. Indeed, it is a common misinterpretation, understandable in light of the examples described by Weir et al.^15^, that CHAMP requires large numbers of input partitions; however, CHAMP will identify an admissible subset from *any* set of partitions, regardless of cardinality, and it is easily confirmed by comparing results with the corresponding package^16^ with different numbers of inputs that the number of partitions in CHAMP’s admissible subset and the main features of the corresponding domains of optimality typically stabilize rapidly in practice, with only slow or modest improvement upon adding further candidate partitions. CHAMP does not itself inherently require a large number of input partitions, though of course the overall quality may improve as the number of input partitions increases. Nevertheless, to be sure our results here are not impacted by a relative lack of input partitions we ran the Louvain algorithm, as implemented by Traag^25^, 10,000,000 times on a uniformly spaced $0\le \gamma \le 2$ grid on a desktop computer (8-core i7-9700K CPU, 16 GB DDR4 3200 MHz) in less than 5 minutes. However, the CHAMP domains are qualitatively similar with only 100 Louvain calls, and we typically obtain the same final pruned fixed point partitions for the karate club with as few as a dozen Louvain calls. In our trial, we found 539 unique partitions, from which CHAMP identifies only 9 partitions as admissible (somewhere optimal), with domains of optimality and $\gamma$ estimates shown in Fig. 2d When the number of communities *K* is left unrestricted, there is only one fixed point: the 4-community partition in Fig. 2d Note this corresponds to the partition that Pamfil et al.’s iterative procedure most frequently converged to in Fig. 2b On the other hand, when the number of communities is restricted prior to running CHAMP, we find exactly one fixed point for each choice of $K \in \{2, 3, \dots , 8\}$. Given the relatively simple behavior observed in Fig. 2a it is unsurprising that the stable 2-community partition remains the same as that identified using Newman’s procedure. Meanwhile, the 4-community fixed point that was stable for unrestricted *K* in Fig. 2d is necessarily also a fixed point when we restrict to partitions with $K=4$ communities.

### Synthetic example with multiple community scales

**Figure 3:**
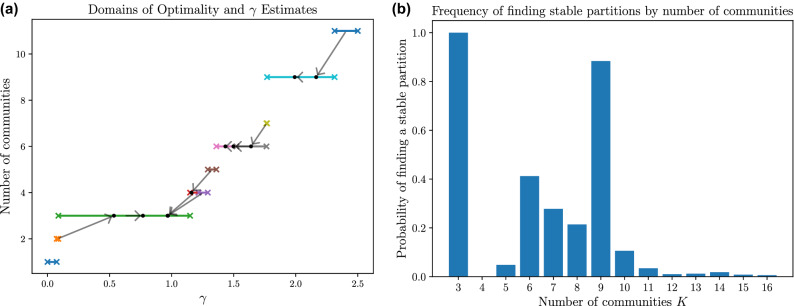
Hierarchical block model networks with 3 communities of 3 subcommunities each. (**a**) The parameter map on a single network realization, plotted in the manner of Figure 2d with each line segment between ‘x’ symbols indicating the domain of optimality of a partition and the “correct” estimate of $\gamma$ indicated by an arrow from its midpoint to corresponding dot. Stable fixed point partitions with 3, 6, and 9 communities are identified by each having its $\gamma$ estimate within its own domain. (**b**) Frequencies of stable partitions of *K* communities on 500 realizations from this model. A 3-community structure is always identified and a stable 9-subcommunity structure appears in ${\sim } 90\%$ of realizations. Other numbers of communities are also frequently identified. Each network realization was partitioned with 1000 runs of Louvain on a uniform grid of $\gamma \in [0,2.5]$.

 The reduction to a deterministic map allows us to more easily handle networks in which there are multiple “correct” values for the resolution parameter. For example, a network with significant community structure at multiple different scales may have one value of $\gamma$ that corresponds to larger communities and a different value corresponding to smaller communities within the larger ones. Having seen this occur in practice, we demonstrate such behavior using a simple hierarchical block model with multiple partitions of a network that are simultaneously stable under the parameter estimation map. Figure 3 shows results from random graphs of 450 nodes generated with 9 equal-sized blocks grouped into 3 communities of 3 subcommunities each, with node pairs within the same subcommunity connected with probability 0.12, within the same community but different subcommunities with probability 0.03, and in different communities with probability 1/600. Figure 3a visualizes the resolution parameter map for a single network realization from this model, simultaneously yielding 3 stable partitions: one well aligned with the 3 planted communities, another aligned with the planted 9-subcommunity split, and a partition that correctly identifies the possibility of subdividing each of the 3 communities but highlights a (presumably random) substructure with 6 communities. Figure 3b explores this behavior by accumulating the frequencies of the stable partitions identified across 500 network realizations from this model.

### Synthetic multilayer temporal network

 We next focus on a synthetic temporal network model used in Pamfil et al.^18^, initially proposed by Ghasemian et al.^27^, generated as follows. Ground-truth community membership in the first layer is split evenly between *K* available community labels. For each subsequent layer, the community label is copied from the previous layer with probability $\eta$; otherwise, the community is randomly assigned from all *K* possible labels. Using these assigned communities, edges are independently placed between pairs of nodes in each layer with probability $p_\text {in}$ for nodes in the same community and with probability $p_\text {out}$ otherwise. The probability ratio $\varepsilon = p_\text {out} / p_\text {in}$ describes the strength of the community structure in these layers (smaller values of $\varepsilon$ placing more edges within than between communities).

**Figure 4:**
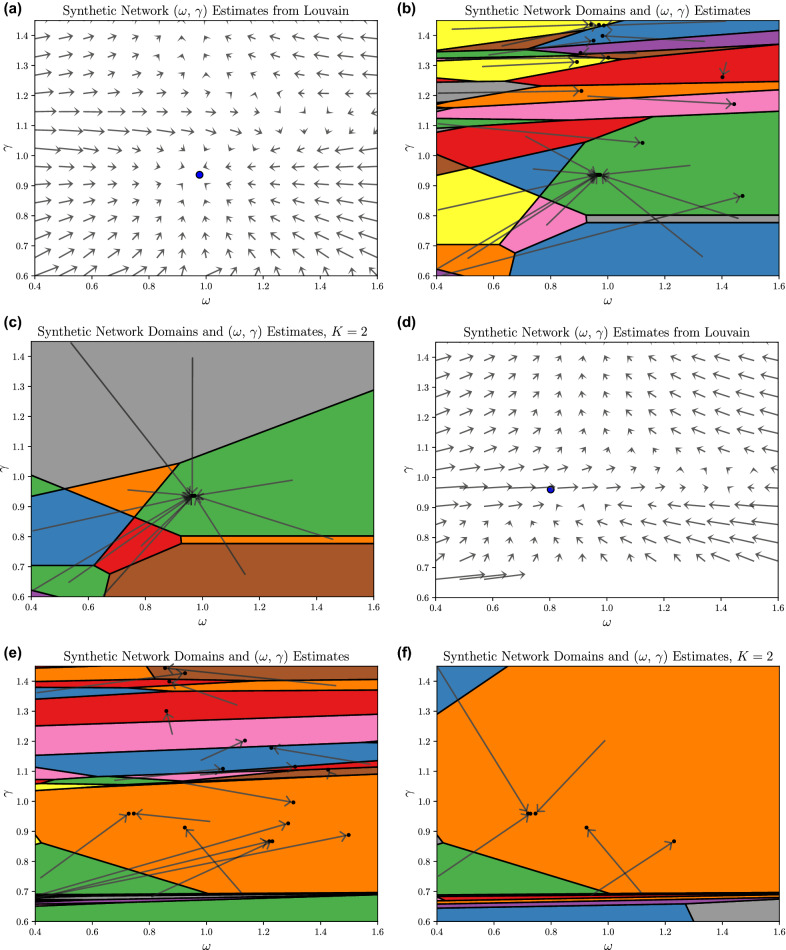
Comparing different iterative procedures on synthetic multilayer networks. Top row: the Pamfil et al.^18^ “easy” regime. (**a**) The Pamfil et al. iterative map visualized on an $(\omega , \gamma )$ grid with arrows (scaled down 10%) indicating the direction of parameter estimates using Louvain at each $(\omega , \gamma )$, averaged over five trials, finding a stable fixed point near the blue point from the ground-truth partition, $(\omega , \gamma ) \approx (0.98, 0.94)$. (**b**) Domains of optimality for the CHAMP set ($\approx$ 25 partitions somewhere optimal in the displayed range), with arrows from the centroid of each domain to its $(\omega , \gamma )$ estimate. (**c**) Domains of optimality and $(\omega , \gamma )$ estimates from CHAMP restricted to $K=2$ communities. Bottom row: the Pamfil et al. “hard” regime: (**d**) The ground-truth $(\omega , \gamma ) \approx (0.80,0.96)$ blue point is unstable under the Pamfil et al. iterative map, whereas the finite state maps on (**e**) the full CHAMP set and (**f**) restricting CHAMP to $K=2$ communities both identify a fixed point near the ground-truth estimates. The absence of arrows for $\gamma \lesssim 0.65$ are from the single-community partition.

The top row of Fig. 4 considers a multilayer network in the “easy” regime of Pamfil et al., with copying probability $\eta = 0.7$, edge probability ratio $\varepsilon = 0.4$, $T = 15$ layers, $K=2$ communities, and 150 nodes per layer. (Note $K=2$ means agreement of labels from one layer to the next actually occurs with probability $\eta + \frac{1}{2}(1-\eta ) = 0.85$.) Pamfil et al.’s iterative procedure on this network, visualized in Fig. 4a converges near to the ground truth parameter estimate for most initial $(\omega , \gamma )$ values with $\gamma \lesssim 1.15$, whereas initializations with $\gamma \gtrsim 1.15$ fail to converge to the ground truth. We ran Louvain 50,625 times on a $225 \times 225$ uniform grid of $\gamma \in [0,2]$, $\omega \in [0,2]$, yielding 27,639 unique partitions with more than one community. CHAMP identifies 91 of these as somewhere optimal, with domains of optimality and associated parameter estimates visualized in Fig. 4b exhibiting the same general behavior as the Pamfil et al. iterative procedure shown in Fig. 4a with many partitions’ estimates close to the ground truth $(\omega , \gamma ) \approx (0.98, 0.94)$. Notably, this 2-community stable partition has very strong alignment with the ground truth (agreeing for ${\sim }99.9\%$ of the node-layers). Also similarly, the partitions optimal at $\gamma \gtrsim 1.2$ do not converge near this ground truth; however, unlike the Pamfil et al. procedure, the iterations here do converge to other stable partitions with many more communities (see SI Section F for details). Given these results, one might naturally focus on the $K=2$ case by restricting CHAMP to only consider those partitions. The Louvain results above included 2,507 unique $K=2$ partitions, 29 of which are somewhere optimal (relative to these $K=2$ partitions), precisely one of which is stable under the parameter estimate map: the same 2-community partition that is stable for unconstrained *K* (Fig. 4c).

We repeat these numerical experiments with $\eta = 0.5$ and $\varepsilon = 0.5$. Figure 4d visualizes this “hard” case from Pamfil et al., similar to their Fig. 3(b). The difficulty with this case comes from the heuristic modularity maximization creating pseudo-random fluctuations that make the fixed point unstable. Indeed, the heuristic sometimes returns partitions at the fixed point with modularity ${\sim }10\%$ lower than for the ground truth. Pamfil et al. circumvent some of these difficulties with an “ad hoc” (their words) reduction of $\gamma$ whenever *K* exceeds an imposed $K_\mathrm {max}$. In contrast, the results across the bottom row of Fig. 4 demonstrate that our finite-state map does not suffer any such problems with this “hard” case, correctly identifying a (stable) fixed point closely matching the planted communities in the generated multilayer network (Fig. 4e). Similar to the “easy” case, we again see even simpler behavior of the iterative map obtained by restricting to $K=2$ partitions (Fig. 4f). We again note that stability of a partition when allowing *K* to vary implies stability under the procedure when restricted to that same *K*.

### Lazega law firm

**Figure 5:**
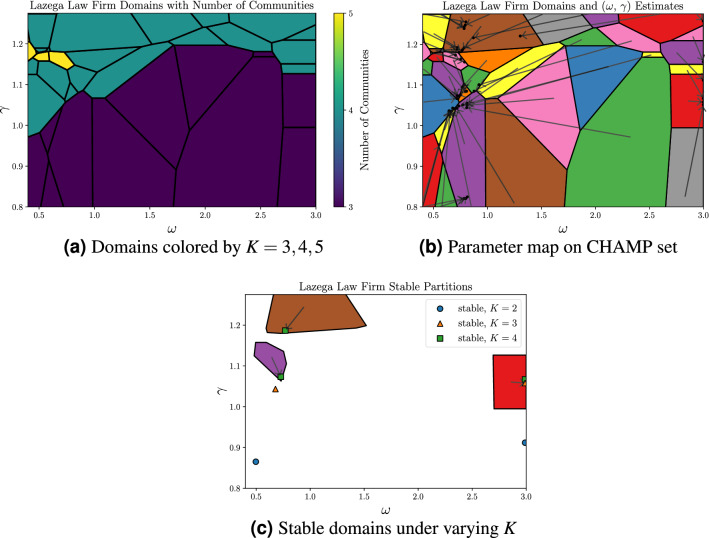
Results for the Lazega law firm multiplex network. (**a**) Domains of optimality for the partitions in CHAMP’s admissible subset. Color indicates number of communities. (**b**) Domains annotated with arrows to indicate each partition’s $(\omega , \gamma )$ parameter estimates. Partitions with identical communities across all layers, yielding an $\omega = \infty$ estimate, are visualized with arrows to $\omega = 3$. (**c**) The domains and parameter estimates for the three stable partitions allowing *K* to vary, with additional points indicating other stable partitions found when separately fixing $K = 2, 3, 4$ prior to running CHAMP.

 Following Pamfil et al.^18^, we now demonstrate our approach on the Lazega Law Firm network^28^, a 3-layer multiplex network that describes the relationships between 71 attorneys who were asked to list members of the firm that they go to for professional advice (“Advice”), closely work with (“Coworker”), and socialize with outside work (“Friend”). In so doing, we note that we utilize Pamfil et al.’s SBM equivalence for directed layers in a multiplex network. We ran 1e6 instances of Louvain over a uniform $2000\times 500$ grid of $\gamma \in [0, 2]$, $\omega \in [0, 3]$, yielding 211,219 unique partitions. When we do not restrict *K*, CHAMP identifies 152 admissible (somewhere optimal) partitions visualized in Fig. 5a, three of which are stable: one with $K=3$ and two with $K=4$. Figure 5c highlights the domains of optimality of these stable partitions, along with parameter estimates of four other partitions that are stable for fixed-*K*-restricted parameter maps with $K=2$, 3, and 4 (another stable partition for $K=2$ has $\gamma < 0.8$ and does not appear in the figure). Comparing with Fig. 4 of Pamfil et al.^18^, their two highlighted groups of partitions that they consensus cluster appear to best correspond to the $K=3$ fixed point with $\omega \approx 0.7$ that we find when restricting to fixed-*K* maps and the stable $K=3$ domain at $\omega =\infty$. Further comparing with the domains and iterative steps in Fig. 5b, one can see the large number of domains mapping into the region that includes both $K=4$ stable points (letting *K* vary) and this $K=3$ point that is stable for fixed-*K* maps, as well as the detail of how close these parameter estimates are to the boundaries of their associated domains. Observing such behavior in practice might lead one to consider adding more runs to obtain additional partitions near this region, to decrease the chance that a somewhere-optimal partition may have been missed. Additionally, one might reasonably choose to directly compare and contrast the relatively few CHAMP partitions obtained near these points, since this region of the parameter space has been effectively highlighted by the map.

### Bounds on $\gamma$ estimates

**Figure 6:**
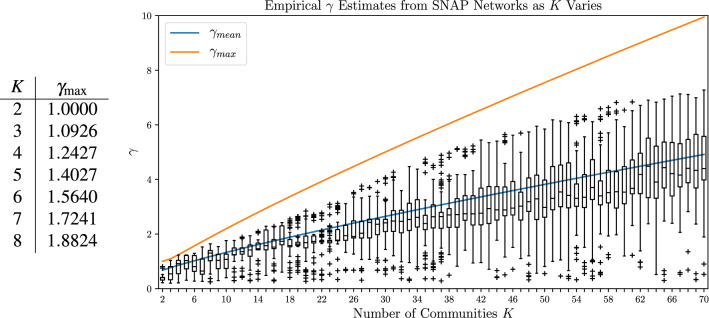
The $\gamma _\text {max}$ bound for SBMs of equal-sized blocks compared with observations on 16 social networks (4k–82k nodes, 17k–948k edges) from the Stanford Large Network Dataset^29^. Box plots collect $\gamma$ estimates for partitions of *K* communities from 1000 Louvain runs on a $\gamma \in [0,10]$ grid on each network. The $\gamma _\text {mean}$ estimate (see SI:J) is also plotted for comparison.

 Throughout the above we have repeatedly started with pre-selected ranges of $\gamma$ and $\omega$. Increasing the interlayer coupling $\omega$ to even modest values such as those in our results forces (nearly) all appearances of a selected node across layers into a single community, so that no further increase changes the partitions. Similarly, there is a maximum meaningful resolution parameter $\gamma$ above which all off-diagonal components of the intralayer modularity matrices are negative, forcing all nodes in a layer into different communities. To better identify useful ranges of $\gamma$, we establish maximum possible $\gamma _\text {max}(K)$ estimates for assortative SBMs of *K* equal-sized blocks (see SI Section J). Figure 6 demonstrates that $\gamma _\text {max}(K)$ empirically bounds the $\gamma$ estimates obtained on a set of real-world networks. Similarly, we note that all $\gamma$ estimates in^17^ are below $\gamma _\text {max}(K)$. Therefore, if a maximum desired number of communities, $K_\text {max}$, is known or can be applied ahead of time, $\gamma _\text {max}(K_\text {max})$ appears to provide an effective bound to further aid in selecting parameter ranges.

## Discussion

We have developed a strategy for pruning sets of partitions obtained by modularity-based community detection algorithms under different parameters and random seeds. We combine the CHAMP post-processing tool of Weir et al.^15^ with iterative procedures based on SBM objective function equivalencies by Newman^17^ for (single-layer) networks and Pamfil et al.^18^ for multilayer networks. By using CHAMP to reduce the number of partitions participating in the iterative procedures for identifying parameters, our strategy transforms the problem into a deterministic map on the finite subset of admissible partitions from CHAMP. The fixed points are then the “stable” partitions that are significant from the perspective of the SBM objective function equivalence. Combining CHAMP with iterative parameter mapping performs better than either method alone, particularly where it greatly reduces the effects of stochasticity due to non-optimal partitions found by the community detection heuristics. Importantly, our combined methodology works for holding the number of communities *K* fixed (as in Newman^17^) or letting *K* vary (as in Pamfil et al.^18^).

One might rightfully be concerned about potentially removing important partitions by using CHAMP, especially given the typically large number of near-optimum partitions^30^ with structures from a seemingly similar template^31^. On the other hand, while one may be understandably tempted to keep a broader collection of partitions obtained by computational heuristics, there is then a natural concern about not knowing the effectively true optimization problem solved there. In contrast, the quality of stable partitions that are fixed points under the iterative procedure are directly related to the likelihood of the underlying planted partition SBM, so we believe that it is reasonable to ignore the nowhere-optimal partitions, at least as a first pass. At the same time, however, because the planted partition equivalent to modularity is highly restrictive, it is of course possible that other partitions that are not fixed points still contain important community structures (see in particular Peel et al.^5^). Users with advanced knowledge will undoubtedly encounter situations where their further exploration yields important observations, but we believe the vast majority of community detection users across different fields of application will benefit from the simplicity of our approach for reconciling multiple partitions obtained across the parameter space.

Moreover, we stress that CHAMP can only post-process partitions that are provided to it as input, so the performance of the full framework is limited by that of whatever community detection heuristics are used to initially find that input set of partitions. That is, if the underlying heuristics fail to find adequately optimum partitions in a modularity sense in the first place, CHAMP cannot improve upon what is in the input set. That said, we note the key role CHAMP plays in the success of our combined framework on the “hard” case synthetic multilayer temporal network (bottom row of Fig. 4): the heuristic used finds partitions in strong agreement with the ground truth elsewhere in the parameter space that CHAMP then identifies as being optimal at the point corresponding to the ground truth, whereas the heuristic run around this point typically returns partitions with modularity $\sim 10\%$ lower than for the ground truth. By pooling and post-processing the full set of input partitions together, CHAMP identifies an appropriate partition near this point even though the heuristic run at that point does not. It is precisely because of this behavior that the iterative map on the CHAMP set yields a fixed point that is inherently stable and in good agreement with the ground truth, emphasizing the value of combining these previously disconnected approaches.

We emphasize that the general methodology of combining CHAMP with iterative parameter maps is very flexible in terms of how the initial set of partitions is obtained and in what order the different concepts are applied. Because of our desire to focus here on the finite-state map on the space of admissible partitions at the end of the process, and the relatively low cost of calling Louvain on the examples considered here, we have opted to first generate a large number of partitions using Louvain at different parameter values in a reasonable range. Indeed, our $\gamma _\text {max}(K)$ bound can be used in practice to help select the range of $\gamma$ considered. Because CHAMP greatly reduces the effect of heuristic-caused stochasticity, we found that we tend to find the stable partitions even when the number of Louvain calls is relatively small. One could combine CHAMP (which is computationally negligible) with iterative maps a la Pamfil et al. without incurring any additional Louvain calls if desired, running the iterations (including stochasticity from the computational heuristic) from different seed points and inputting all partitions obtained into CHAMP to define the map on the admissible subset and find the stable partitions that are its fixed points. Alternatively, since the Qhull^32^ implementation of halfspace intersection supports incrementally added halfspaces, one could update the admissible subset and domains of optimality as each new partition is found.

Future work could further analyze the equivalencies between modularity maximization and SBM inference, especially in terms of applying different information criteria for varying *K*, and extend the implementation to parameter spaces with more than two dimensions, especially for some of the other interlayer couplings considered by Pamfil et al.^18^ where parameters vary across layers. The current implementation of our methodology only considers parameter spaces with one dimension (i.e., $\gamma$) or two dimensions. Typically these are the resolution parameter $\gamma$ and interlayer coupling $\omega$ in our development here, but could alternatively be two multipliers of other parameters varying between layers as in some of the higher-dimensional parameter spaces of Pamfil et al. The use of Qhull in CHAMP restricts the dimensionality of the parameter space in practice, but one might also develop a scheme using pseudo-random results on higher-dimensional parameter spaces to then re-cast to a lower-dimensional space where a map could again be defined on the appropriate admissible subset. Theoretically, the equivalence with SBM inference is only known for unweighted multigraphs; it would be particularly important to explore whether any extended interpretation of the related formulae to weighted graphs may be appropriate or to identify some other equivalence. Finally, while we conjecture that parameter estimation orbits (beyond simple fixed points) cannot occur for assortative partitions in our iterative maps, we have neither a proof nor a counterexample of this property.

## Methods

We aim here to provide the essential, high-level information about each method in a common notation, modifying that of the cited works where needed, to aid the reader’s understanding. Complete details about each method are in the cited works.

### Modularity

 Modularity for undirected networks^7^ after including a resolution parameter^14^ is given by

1 $$\begin{aligned} Q = \frac{1}{2m} \sum _{i, j} \left[ A_{ij} - \gamma \frac{k_i k_j}{2m}\right] \delta (g_i, g_j)\,, \end{aligned}$$

where *A* is the adjacency matrix ($A_{ij} = 1$ when nodes *i* and *j* are connected and $A_{ij} = 0$ otherwise), *m* is the total edge weight, $k_i$ is the weighted degree of node *i* (the total weight of edges connected to *i*), $g_i$ is the community/group label of node *i*, and $\delta$ is the Kronecker delta with $\delta (g_i, g_j) = 1$ when $g_i = g_j$ and 0 otherwise. Newman and Girvan’s original definition, corresponding to $\gamma = 1$, measures the total weight of edges within the communities minus that expected in a random model with the same expected degree sequence. The resolution parameter $\gamma$ introduced^14^ in part to overcome issues resolving communities in large networks^33^ can be used to detect communities at different scales: small $\gamma$ favors partitions with a few large communities; as $\gamma$ increases, one tends to find larger numbers of smaller communities (see also Arenas et al.^34^). While the descriptive nature of modularity has many shortcomings compared to generative models, modularity maximization remains one of the most popular methods for community detection, in part because fast heuristics are readily available across computational environments. The Louvain^10^ algorithm is particularly widely used, while the newer Leiden^11^ algorithm promises greater improvements.

### Multilayer modularity

 Mucha et al.^12^ generalized modularity to (what are now known as) multilayer networks^13^ by leveraging the relationship between modularity and Laplacian dynamics^35,36^. Consider a set of *T* layers of $n \times n$ adjacency matrices ${\mathbf {A}}^t$, $1 \le t \le T$, each representing the same set of *n* nodes. (Different sets of nodes in different layers can also be handled, as demonstrated by the Senate roll call example in Mucha et al.^12^.) The simplest multilayer cases involve a set of interlayer couplings $C^{sr}$, one for each pair of distinct layers $1 \le s, r \le T$ such that node *j* in layer *s* is connected to itself in layer *r* with weight $C_{j}^{sr}$. Then, the goal is to determine group membership per node-layer, i.e. the assignment $g_i^s$ of node *i* in layer *s*, by maximizing the multilayer modularity^12^ (assuming undirected layers here, though other intralayer model contributions may be selected as appropriate)

2 $$\begin{aligned} Q = \frac{1}{2\mu } \sum _{ijsr} \left[ \left( A_{ij}^s - \gamma _s \frac{k_i^s k_j^s}{2m_s}\right) \delta (s,r) + C_j^{sr}\delta (i,j)\right] \delta \left( g_i^s,g_j^r\right) \,, \end{aligned}$$

where $k_i^s$ is the degree of node *i* in layer *s*, $m_s$ is the number of edges in layer *s*, and $2\mu = \sum _{is}\left( k_i^s + \sum _r C^{sr}_i\right)$ is twice the sum of all intralayer and interlayer edge weights. Note that in principle each layer may have a different “intralayer resolution parameter” with the weighting of the null model in layer *s* being controlled by $\gamma _s$, though in many cases in practice one simply selects $\gamma _s=\gamma$ constant across layers. In the simplest settings used in Mucha et al.’s examples, the interlayer coupling $C^{sr}$ elements take values $\{0,\omega \}$ corresponding, respectively, to absence and presence of an interlayer link. This particular choice is known as uniform (interlayer) coupling^37^ since the weights of all of the present interlayer couplings are identical. Multilayer modularity extends naturally to more complicated settings by defining and summing appropriately over the interlayer edges.

### Newman’s equivalence between modularity maximization and SBM inference

The Stochastic Block Model (SBM) approach instead approaches community detection as an inference problem fitting network data to a generative model. Newman^17^ demonstrated that the objective functions for modularity maximization and statistical inference on SBMs become equivalent under certain conditions. Specifically, consider a degree-corrected version of a “planted partition” SBM with expected degree sequence matching the observed sequence, such that node *i* will on average have $k_i$ neighbors, and the number of edges between nodes *i* and *j* are independently Poisson distributed with mean $\frac{k_i k_j}{2m} \theta _{g_i g_j}$ (or half this value when $i=j$), where $2m=\sum _i k_i$ and the $\theta _{\alpha \beta }$ elements take two values: one shared by all diagonal entries, $\theta _\text {in}$, and another shared by all off-diagonal entries, $\theta _\text {out}$, so that all communities have the same in-group and between-group connection propensities. Neglecting constants that do not alter the argmax of the expression, simplification of the log-likelihood (under very specific constraints^38^) yields^17^

3 $$\begin{aligned} \ln P({\mathbf {A}} \mid \theta _\text {in}, \theta _\text {out}, {\mathbf {g}}) = \sum _{ij} \left[ A_{ij} - \frac{k_i k_j}{2m} \cdot \frac{\theta _\text {in} - \theta _\text {out}}{\ln \theta _\text {in} - \ln \theta _\text {out}}\right] \delta (g_i, g_j)\,. \end{aligned}$$

The above expression is recognizably equivalent to (1) when

4 $$\begin{aligned} \gamma = \frac{\theta _\text {in} - \theta _\text {out}}{\ln \theta _\text {in} - \ln \theta _\text {out}}\,. \end{aligned}$$

In this way, this choice of $\gamma$ is the “correct” value of the resolution parameter if one wishes to make modularity maximization equivalent to the maximum likelihood fit of a planted partition, degree-corrected stochastic block model. We will often call this the “$\gamma$ estimate” or “resolution parameter estimate” of a partition. Newman^17^ then gives an iterative procedure to find this correct choice of $\gamma$. First, note that the expected number of within-community edges in this model is

$$\begin{aligned} m_\text {in} = \frac{1}{2} \sum _{ij} \left[ \frac{k_i k_j}{2m} \cdot \theta _\text {in} \cdot \delta (g_i, g_j)\right] = \frac{\theta _\text {in}}{4m} \sum _c \kappa _c^2\,, \end{aligned}$$

where $\kappa _c = \sum _i k_i \delta (g_i,c)$ is the sum of the degrees of all nodes in group *c*. Then, we can estimate

5 $$\begin{aligned} \theta _\text {in} = \frac{2m_\text {in}}{\sum _c \kappa _c^2 / (2m)}, \qquad \theta _\text {out} = \frac{2m_\text {out}}{\sum _{c \ne g} \kappa _c \kappa _g / (2m)} = \frac{2m - 2m_\text {in}}{2m - \sum _c \kappa _c^2 / (2m)}\,. \end{aligned}$$

Thus, with an initial guess for $\gamma$, one can repeatedly maximize modularity (with the number of communities fixed) and compute new estimates for $\theta _\text {in}$ and $\theta _\text {out}$. This gives a new value for $\gamma$ and we repeat until convergence.

### Pamfil et al.’s generalizations for multilayer networks

 Pamfil et al.^18^ generalized Newman’s^17^ equivalence to several variants of multilayer networks. While many different multilayer network settings are possible^13^, Pamfil et al.’s extension focuses on three types: “temporal”, “multilevel”, and “multiplex” networks. Pamfil et al.^18^ show that multilayer modularity maximization with specific resolution and coupling parameters is equivalent to statistical inference on corresponding multilayer stochastic block models. Generalizing Newman’s strategy^17^, they consider the intralayer connections in layer *t* given by ${\mathbf {A}}^t$ to be drawn from a degree-corrected, planted-partition stochastic block model. Temporal networks are those in which each layer encodes interactions during some period or instance of time. The underlying SBM model further assumes that labels are copied between layers with “copying probability” *p*. That is, the ground truth group assignment $g_i^t$ of node *i* in layer *t* is copied from layer $t-1$ with probability *p* and is with probability $1-p$ assigned randomly according to a uniform distribution across the *K* labels. Consider a partition ${\mathbf {g}}$ of the multilayer network where $g_i^t$ is the group membership of node *i* in layer *t*. After substantial simplification, Pamfil et al. reduce $\ln P({\mathbf {g}} \mid {\mathbf {A}}, \theta _\text {in}, \theta _\text {out}, p, K)$ to multilayer modularity (2) with

6 $$\begin{aligned} \gamma = \frac{\theta _\text {in} - \theta _\text {out}}{\ln \theta _\text {in} - \ln \theta _\text {out}} \qquad \text {and}\qquad \omega = \frac{\ln \left( 1 + \frac{p}{1-p}K\right) }{\ln \theta _\text {in} - \ln \theta _\text {out}}\,, \end{aligned}$$

Hence, these $\gamma$ and $\omega$ are the “correct” values of the intralayer resolution and interlayer coupling parameters to make multilayer modularity maximization equivalent to the maximum likelihood fit of the considered temporal network SBM. Like before, we will call the values in Equation 6 the “$\gamma$ estimate” and “$\omega$ estimate” (together, “parameter estimates”) of a partition. The $\theta _\text {in}$ and $\theta _\text {out}$ are estimated in much the same way as the corresponding propensities in the single-layer case, with the added restriction that group memberships are considered per layer rather than in aggregate. The copying probability *p* of labels from one layer to the next is empirically estimated using the observed frequency with which the group membership of node *i* persists across layers — i.e. one estimates *p* by calculating the probability that $g_i^{t-1} = g_i^t$ over all layers $t = 2, \dots , T$ and all nodes $i = 1, \dots , N$. One can then iteratively find “correct” values for $\gamma$ and $\omega$ by maximizing modularity and computing new estimates, repeating until convergence. We specially note that Pamfil et al. consider multiple models beyond those considered in the current implementation of our framework, including different multilayer topologies, parameters that vary across the multilayer network, and multilevel networks; for details, see the SI (Section B) and Pamfil et al.^18^.

### CHAMP

 Weir et al.^15^ developed the Convex Hull of Admissible Partitions (CHAMP) algorithm to post-process sets of network partitions in order to identify regions of modularity optimization. Given an input set of partitions, however obtained (e.g., by different methods, under different parameters, or even through non-algorithmic means), CHAMP identifies domains of the resolution-coupling parameter space for which each partition has the largest (multilayer) modularity relative to the input set of partitions. In practice many partitions are nowhere optimal. The somewhere-optimal partitions are then referred to as the “admissible” or “CHAMP” subset. In particular, because of the form of multilayer modularity, the domain of optimality of each partition is necessarily convex in the parameter space, leading to the (convex) polygonal domains in our Figures. For more details, see the SI (Section C), Weir et al.^15^and the CHAMP package^16^.

### ModularityPruning implementation

 The repository http://github.com/ragibson/ModularityPruning includes our modularitypruning Python library that implements our pruning pipeline. The library is available for installation through the Python Package Installer (pip).

## Supplementary Information

Supplementary Information.

## Data Availability

The repository http://github.com/ragibson/ModularityPruning also includes the code and data used to generate the results presented here and in the Supplementary Information.
