# Supplementary material for: Finite-state parameter space maps for pruning partitions in modularity-based community detection
Source: Sci Rep. 2022 Sep 23;12:15928. doi: 10.1038/s41598-022-20142-6 (PMC9508178; doi:10.1038/s41598-022-20142-6)
Supplement: Supplementary file 1 — Supplementary Information. [file 41598_2022_20142_MOESM1_ESM.pdf]

Supplementary Information for Finite-State Parameter Space Maps for Pruning Partitions in Modularity-Based Community Detection

Ryan A. Gibson[1,2,†] and Peter J. Mucha[1,3,4,*]

[1]Department of Mathematics, University of North Carolina, Chapel Hill, NC 27599-3250, USA
[2]Department of Computer Science, University of North Carolina, Chapel Hill, NC 27599-3175, USA
[3]Department of Applied Physical Sciences, University of North Carolina, Chapel Hill, NC 27599-3050, USA
[4]Department of Mathematics, Dartmouth College, Hanover, NH 03755-3551, USA
[†]ryan.alexander.gibson@gmail.com
[*]peter.j.mucha@dartmouth.edu (corresponding author)

The format of this supplementary information is as follows.

- In section A, we describe Newman's [1] equivalence between modularity maximization and a restricted form of stochastic block model inference. Our discussion includes details not explicitly detailed within the original paper and several interesting consequences.
- In section B, we describe Pamfil et al.'s [2] extension of Newman's equivalence to multilayer settings. We also demonstrate issues that arise from Pamfil et al.'s use of modularity maximization heuristics that do not keep the number of communities fixed.
- In section C, we describe Weir et al.'s CHAMP (Convex Hull of Admissible Partitions) algorithm [3, 4] for identifying regions of modularity optimization and the algorithm's importance in our pruning procedure.
- In section D, we present a few additional results on the Zachary karate club [5]. This briefly expands on the discussion from section B.5.
- In section E, we test our method on a synthetic hierarchical block model containing planted community structures at two different scales.
- In section F, we test our method on synthetic networks from Pamfil et al. [2]. We show that our method recovers the ground truth community structure in their "easy case" as well as the "hard case" where their method fails to converge. Moreover, we show that our method continues to recover this community structure, even as the number of input partitions is reduced to 25 (approximately the number of partitions gathered in a single run of Pamfil et al.'s iterative method).
- In section G, we test our method on the Lazega Law Firm network [6] and compare our results to that of Pamfil et al. [2]. Without fixing the number of communities, our method identifies stable partitions that agree with the common convergence points of Pamfil et al.'s iterative algorithm, potentially with less computational cost. Then, by fixing the number of communities, we find multiple additional stable partitions and show that these are strongly aligned with the network's ground truth metadata.
- In section H, we explicitly construct a simple network model in which two ground truth partitions, one with 2 communities and one with 3 communities, are simultaneously stable. This further demonstrates our method is able to capture meaningful community structure at multiple different scales.
- In section I, we show that our method yields reasonable results on synthetic networks where the assumptions of the equivalence are not satisfied, namely, LFR networks whose degree and community size sequences follow power laws.
- In section J, we derive upper bounds on the resolution parameter appropriate for fitting to the SBM from Newman's equivalence, potentially providing a guide to where in the parameter space modularity maximization heuristics should be run to obtain a specified number of communities. We demonstrate that these bounds hold in practice by computing γ estimates on various "real-world" networks.
- In section K, we show that our method performs quickly compared to Louvain and that practical convergence issues arising from the possibility of periodic orbits (beyond fixed points) in the iterative procedure appear unlikely to exist.

In order to provide a more complete narrative, some elements of the main text and methods sections are repeated in our expanded discussion within the present supplementary information document.

```
import igraph as ig
from modularitypruning import prune_to_stable_partitions
from modularitypruning.louvain_utilities import repeated_louvain_from_gammas
import numpy as np

# get Karate Club graph in igraph
G = ig.Graph.Famous("Zachary")

# run louvain 1000 times on this graph from gamma=0 to gamma=2
partitions = repeated_louvain_from_gammas(G, np.linspace(0, 2, 1000))

# prune to the stable partitions from gamma=0 to gamma=2
stable_partitions = prune_to_stable_partitions(G, partitions, 0, 2)
print(stable_partitions)
```

Figure S1. Example usage of our modularitypruning Python package. This code repeatedly runs the Louvain modularity maximization heuristic on the Karate Club network of Zachary [5] and prints the stable partition with four communities returned by our pruning method.

The source code for the experiments discussed here is available at `https://github.com/ragibson/ModularityPruning`. This repository also includes the `modularitypruning` Python library that implements our pruning pipeline, also available for installation through the Python Package Installer (pip). This package primarily interfaces with the popular network analysis library igraph [7], CHAMP [4], and louvain-igraph [8] through their respective Python packages. We direct the interested reader to the software package itself, but provide a simple code example in Figure S1 so as to indicate its relative ease of use.

A Details and Consequences of Newman's Equivalence

This section focuses on Newman's equivalence between modularity maximization and a special case of stochastic block model inference presented in [1], with extra elucidation intended to help with its use in our present work. Afterwards, starting in section A.5, we discuss a few interesting consequences of the equivalence.

A.1 Review of Modularity Maximization

One of the most popular methods for community detection is to heuristically maximize a quantity known as modularity, which for unweighted networks and the standard Newman-Girvan [9] null model is given by

$$Q = \frac{1}{2m}\sum_{i,j}\left[A_{ij} - \gamma\frac{k_i k_j}{2m}\right]\delta(c_i, c_j), \tag{S1}$$

where A is the adjacency matrix of the network ($A_{ij} = 1$ when nodes i and j are connected and $A_{ij} = 0$ otherwise), m is the number of edges in the network, k_i is the degree of node i (the number of edges connected to node i), g_i is the community/group label of node i, and δ is the Kronecker delta function so that $\delta(g_i, g_j) = 1$ when nodes i and j are in the same community (i.e. $g_i = g_j$) and is 0 otherwise. (For weighted networks, all terms are to be interpreted through their weights, not counts of edges.)

In Newman and Girvan's original definition, $\gamma = 1$ so that Q gives a measure of how many more edges are observed in the network's communities than would be expected in a random graph with expected degree sequence equal to the observed sequence. That is, if the communities in a partition are much more densely connected than one would expect by random chance alone, the partition has a "high" value of Q. The "resolution parameter" γ was added by Reichardt and Bornholdt [10] to overcome resolution issues in large networks (see also the approach proposed by Arenas et al. [11]). In particular, when the network is very large in comparison to its communities, modularity with $\gamma = 1$ can fail to detect some community structure (in such cases, merging two communities may increase Q even when the connections between them are very weak [12]). The γ prefactor helps resolves this issue by allowing one to change the relative penalty paid (here, the $\frac{k_i k_j}{2m}$ term) for putting nodes into the same community, thus allowing for detecting communities at different scales — small γ tends to lead to a few large communities, and increasing γ tends to find a larger number of smaller communities. This can be particularly useful since the null model penalty used in modularity does not describe the network well when the community sizes vary drastically.

Exact optimization of modularity is NP-Hard [13]; specifically, for any constant $\rho > 0$, it is NP-Hard to find a partition of a network with modularity at least $\rho \cdot Q_{\text{opt}}$ where Q_{opt} is the optimal modularity over all possible partitions [14, 15]. Regardless, fast heuristics exist for its maximization (perhaps most notably the Louvain [16] and Leiden [17] algorithms), undoubtedly contributing to the fact that modularity maximization remains one of the most popular methods for detecting communities in real-world networks.

A.2 Stochastic Block Model Inference

Another popular method for detecting communities is to fit a generative model known as a "stochastic block model" to the network of interest. Importantly, this method is statistically principled rather than being ad hoc or motivated through heuristics alone. In general, one divides a set of n nodes into K groups and denotes the group membership of node i by g_i. Additionally, a matrix Ω is specified whose elements determine the connection strengths between the various communities; the higher the value for $(\Omega)_{rs} = \omega_{rs}$, the greater the number of edges between groups r and s.

One of the simplest versions of these models considers every possible edge between nodes in groups r and s to exist with probability ω_{rs} (and thus, no edge to exist with probability $1-\omega_{rs}$). In this way, the diagonal of the Ω matrix determines the internal connection densities of the K communities and the off-diagonal terms specify the density of connections between communities. (To make the model even simpler, one might set all of the diagonal elements of Ω to one value and all its off-diagonal elements to another value.)

Unsurprisingly, this simple model does not fit real-world networks well — as Newman notes in [1], "there are no good fits when the model you are fitting is simply wrong" — and as is common across most community detection methods, many different variants exist. For the remainder of this section, we will direct our focus on the "degree-corrected stochastic block model", which can fit networks with arbitrary degree distributions. Much of the discussion here is adapted from [1, 18], but we've altered the notation to more closely match other parts of this document. In addition to the group assignments and Ω matrix, we assign an expected degree to each node of the network such that node i will on average have k_i neighbors. Then, the number of edges between nodes i and j are independently Poisson distributed with mean $\frac{k_i k_j}{2m}\omega_{g_i g_j}$ or half this value when $i = j$.

Naturally, when fitting such a model to a network, one chooses the observed degree sequence for the k_i's and the observed number of edges for m. With this information, we can determine the probability that a partition of a network with adjacency matrix $\mathbf{A}$ and group assignments $\mathbf{g}$ was drawn from this stochastic block model by simply considering the existence of each possible edge one-by-one:

$$
\begin{aligned}
P(\mathbf{A} \mid \Omega, \mathbf{g}) &= \left[\prod_i \frac{\left(\frac{1}{4m}k_i^2\omega_{g_i g_i}\right)^{A_{ii}/2}}{(A_{ii}/2)!} e^{-\frac{1}{4m}k_i^2\omega_{g_i g_i}}\right] \cdot \left[\prod_{i<j} \frac{\left(\frac{1}{2m}k_i k_j \omega_{g_i g_j}\right)^{A_{ij}}}{A_{ij}!} e^{-\frac{1}{2m}k_i k_j \omega_{g_i g_j}}\right] \\
\ln P(\mathbf{A} \mid \Omega, \mathbf{g}) &= \sum_i \left\{\frac{1}{2}A_{ii}\ln\left(\frac{1}{4m}k_i^2\omega_{g_i g_i}\right) - \ln\left[(A_{ii}/2)!\right] - \frac{1}{4m}k_i^2\omega_{g_i g_i}\right\} \\
&\quad + \sum_{i<j}\left[A_{ij}\ln\left(\frac{1}{2m}k_i k_j \omega_{g_i g_j}\right) - \ln(A_{ij}!) - \frac{1}{2m}k_i k_j \omega_{g_i g_j}\right].
\end{aligned}
$$

For the purposes of optimization, we may neglect constants that do not alter the argmax of this expression and simplify this log-likelihood to

$$
\begin{aligned}
\ln P(\mathbf{A} \mid \Omega, \mathbf{g}) &= \sum_i \left\{\frac{1}{2}A_{ii}\ln\left(\frac{1}{4m}k_i^2\omega_{g_i g_i}\right) - \cancel{\ln\left[(A_{ii}/2)!\right]} - \frac{1}{4m}k_i^2\omega_{g_i g_i}\right\} \\
&\quad + \sum_{i<j}\left[A_{ij}\ln\left(\frac{1}{2m}k_i k_j \omega_{g_i g_j}\right) - \cancel{\ln(A_{ij}!)} - \frac{1}{2m}k_i k_j \omega_{g_i g_j}\right] \\
&= \sum_{i,j}\left[A_{ij}\ln\omega_{g_i g_j} - \frac{1}{2m}k_i k_j \omega_{g_i g_j}\right] + \cancel{\sum_{i,j}\left[A_{ij}\ln\left(\frac{1}{2m}k_i k_j\right)\right]} \\
\ln P(\mathbf{A} \mid \Omega, \mathbf{g}) &= \sum_{i,j}\left(A_{ij}\ln\omega_{g_i g_j} - \frac{k_i k_j}{2m}\omega_{g_i g_j}\right). \qquad \text{(S2)}
\end{aligned}
$$

Essentially, partitions of a network whose groups exhibit strong community structure consistent with Ω will have "large" log-likelihoods in this equation. Hence, it is possible to heuristically maximize this quantity with respect to Ω and $\mathbf{g}$ to find the most likely set of group assignments under this generative model.

A.3 Newman's Equivalence Between Modularity Maximization and Maximum Likelihood of a Stochastic Block Model

Newman demonstrated that these two schemes of community detection, modularity maximization and statistical inference based on stochastic block models, become equivalent under certain conditions (see [19] for extensive discussion of the underlying constraints). In this section, we briefly review his primary results from [1].

Consider a restricted version of the degree-corrected stochastic block model discussed in section A.2 where the Ω matrix only takes on two values: one shared by all diagonal entries and another shared by all off-diagonal entries. That is,

$$\omega_{rs} = \begin{cases} \omega_{\text{in}}, & \text{if } r = s, \\ \omega_{\text{out}}, & \text{if } r \neq s \end{cases}$$

so that all communities have the same within-group and between-group connection propensities. This particular case is called a "planted partition model". (More commonly, "planted partition" refers to the case in which nodes are connected with probabilities p_{in} and p_{out}, depending on community membership, but we are considering a degree-corrected version here.) Following Newman, note that this allows us to write

$$\begin{aligned} \omega_{rs} &= (\omega_{\text{in}} - \omega_{\text{out}})\delta(r,s) + \omega_{\text{out}} \\ \ln \omega_{rs} &= \ln\left(\frac{\omega_{\text{in}}}{\omega_{\text{out}}}\right)\delta(r,s) + \ln \omega_{\text{out}} \\ &= (\ln \omega_{\text{in}} - \ln \omega_{\text{out}})\,\delta(r,s) + \ln \omega_{\text{out}}\,. \end{aligned}$$

Using these equations one can rewrite the objective function from Equation S2 for optimizing the log-likelihood that an observed graph fits a degree-corrected SBM,

$$\begin{aligned} \ln P(\mathbf{A} \mid \Omega, \mathbf{g}) &= \sum_{i,j}\left(A_{ij} \ln \omega_{g_i g_j} - \frac{k_i k_j}{2m}\omega_{g_i g_j}\right) \\ &= \sum_{i,j}\left\{A_{ij}\left[\ln\left(\frac{\omega_{\text{in}}}{\omega_{\text{out}}}\right)\delta(g_i,g_j) + \ln \omega_{\text{out}}\right] - \frac{k_i k_j}{2m}\left[(\omega_{\text{in}} - \omega_{\text{out}})\delta(g_i,g_j) + \omega_{\text{out}}\right]\right\} \\ &= \ln\left(\frac{\omega_{\text{in}}}{\omega_{\text{out}}}\right)\sum_{i,j}\left[A_{ij} - \frac{k_i k_j}{2m}\cdot\frac{\omega_{\text{in}} - \omega_{\text{out}}}{\ln \omega_{\text{in}} - \ln \omega_{\text{out}}}\right]\delta(g_i,g_j) \\ &\quad + \sum_{i,j}\left[A_{ij} \ln \omega_{\text{out}} - \frac{k_i k_j}{2m}\omega_{\text{out}}\right]. \end{aligned}$$

Once again, we may ignore constants that do not affect optimization to obtain

$$\begin{aligned} \ln P(\mathbf{A} \mid \Omega, \mathbf{g}) &= \cancel{\ln\left(\frac{\omega_{\text{in}}}{\omega_{\text{out}}}\right)}\sum_{i,j}\left[A_{ij} - \frac{k_i k_j}{2m}\cdot\frac{\omega_{\text{in}} - \omega_{\text{out}}}{\ln \omega_{\text{in}} - \ln \omega_{\text{out}}}\right]\delta(g_i,g_j) \\ &\quad + \sum_{i,j}\left[\cancel{A_{ij} \ln \omega_{\text{out}}} - \cancel{\frac{k_i k_j}{2m}\omega_{\text{out}}}\right] \\ &= \sum_{i,j}\left[A_{ij} - \frac{k_i k_j}{2m}\cdot\frac{\omega_{\text{in}} - \omega_{\text{out}}}{\ln \omega_{\text{in}} - \ln \omega_{\text{out}}}\right]\delta(g_i,g_j)\,, \end{aligned}$$

Note the striking resemblance to modularity; in particular, maximizing this expression is exactly the same as maximizing

$$Q = \frac{1}{2m}\sum_{i,j}\left[A_{ij} - \gamma\frac{k_i k_j}{2m}\right]\delta(c_i,c_j)$$

when

$$\gamma = \frac{\omega_{\text{in}} - \omega_{\text{out}}}{\ln \omega_{\text{in}} - \ln \omega_{\text{out}}}\,. \tag{S3}$$

In this way, this choice of γ is the "correct value" of the resolution parameter if we wish to make modularity maximization equivalent to the maximum likelihood fit of a planted partition, degree-corrected stochastic block model. When ω_{in} and ω_{out} are empirical estimates from a partition, we will often call this the "γ estimate" or "resolution parameter estimate" of the partition.

Additionally, Newman gives an iterative procedure to find a partition of nodes into communities self-consistent with this correct choice of γ. First, note that the expected number of within-community edges in this model is

$$m_{\text{in}} = \frac{1}{2}\sum_{i,j}\left[\frac{k_i k_j}{2m}\cdot\omega_{\text{in}}\cdot\delta(g_i,g_j)\right] = \frac{\omega_{\text{in}}}{4m}\sum_r \kappa_r^2\,,$$

where $\kappa_r = \sum_i k_i \delta(g_i, r)$ is the sum of the degrees of all nodes in group r, sometimes called the "strength" of the group/community. Then, we can empirically estimate

$$\omega_{\text{in}} = \frac{2m_{\text{in}}}{\sum_r \kappa_r^2/(2m)}, \qquad \omega_{\text{out}} = \frac{2m_{\text{out}}}{\sum_{r \neq s} \kappa_r \kappa_s/(2m)} = \frac{2m - 2m_{\text{in}}}{2m - \sum_r \kappa_r^2/(2m)}. \tag{S4}$$

Thus, with an initial guess for γ, we can repeatedly maximize modularity *with the number of communities fixed* and compute new estimates for ω_{in} and ω_{out}. This gives a new value for γ and we repeat until convergence. Moreover, when considering networks drawn from a planted partition model, this scheme is guaranteed to converge in the limit of large node degrees (subject to being able to perform the optimizations correctly). For real-world networks — which are not typically drawn from such planted partitions — no such guarantee can be made, but the procedure still appears to be efficient in practice.

Strictly speaking, this equivalence only holds in its fullest sense if the number of communities is fixed during the maximization of modularity. Otherwise, maximizing modularity is akin to (but not exactly the same as) the simultaneous maximum likelihood fit between many different SBMs, each with a different number of blocks K and potentially different parameters ω_{in} and ω_{out}, but we defer a more in-depth discussion to section A.8. Most importantly, since the planted partition only has two propensity parameters, independent of the number of blocks K, the effect of the additional freedom provided by increasing the number of available community labels is not as great as for general SBMs (where the number of propensity parameters increases quadratically with K). That said, it is perhaps unsurprisingly that this method of statistical inference among many different SBMs is not common in the literature (though some recent work has focused on choosing priors that allow for comparison between different models [20]) and is one of the reasons that modularity maximization is not used to infer the number of communities in a network in Newman's approach. Indeed, in discussing the equivalence, Newman notes that

> "Maximization of modularity with [the number of communities] allowed to vary does not, in general, give good estimates of the number of communities in a network, and it is certainly possible that we get different and incorrect numbers of communities were [the number of communities] allowed to vary." [1]

Unfortunately, in practice the number of communities in a network is not known a priori, even more so in community detection in multilayer networks. Indeed, one of the goals of community detection is to find statistically significant clusters of nodes without considering how many exist in the system that the network is drawn from. As such, the most widely used heuristics (e.g. the Louvain algorithm [16]) do not keep the number of communities fixed. We will return to this issue in section B.5 and section A.8.

A.4 Equivalence to Modularity Maximization Holds Only When $\omega_{\text{in}} > \omega_{\text{out}}$

Recall from section A.2 and section A.3 that finding the maximum likelihood fit of a planted partition, degree-corrected SBM amounts to maximizing

$$\ln P(\mathbf{A} \mid \Omega, \mathbf{g}) = \frac{1}{2} \ln\left(\frac{\omega_{\text{in}}}{\omega_{\text{out}}}\right) \sum_{i,j} \left[A_{ij} - \frac{k_i k_j}{2m} \cdot \frac{\omega_{\text{in}} - \omega_{\text{out}}}{\ln \omega_{\text{in}} - \ln \omega_{\text{out}}}\right] \delta(g_i, g_j) + m(\ln \omega_{\text{out}} - \omega_{\text{out}}) \tag{S5}$$

and this becomes equivalent to the optimization of (single-layer) modularity

$$Q = \frac{1}{2m} \sum_{i,j} \left[A_{i,j} - \gamma \frac{k_i k_j}{2m}\right] \delta(c_i, c_j) \tag{S6}$$

when the resolution parameter takes value

$$\gamma = \frac{\omega_{\text{in}} - \omega_{\text{out}}}{\ln \omega_{\text{in}} - \ln \omega_{\text{out}}}. \tag{S7}$$

This equivalence is to modularity *maximization* only when $\omega_{\text{in}} > \omega_{\text{out}}$ so that the underlying SBM exhibits assortative community structure (i.e. its communities are more densely connected internally than they are to the rest of the network). Note that earlier we had ignored the leading $\ln(\omega_{\text{in}}/\omega_{\text{out}})$ term in Equation S5, claiming that it does not affect optimization. However, consider the case in which the SBM has disassortative structure with $\omega_{\text{out}} > \omega_{\text{in}}$ so that nodes are more preferentially connected to nodes in other groups than nodes in their own group. In this case, the multiplicative prefactor leading in front of the summation becomes negative. Indeed, when $\omega_{\text{out}} > \omega_{\text{in}}$, the maximization of the likelihood is actually equivalent to the *minimization* of modularity. When we refer to "community detection" in the context of modularity, however, one is almost always referring to the assortative case, so we had ignored this situation in the discussion earlier. Note however that disassortative cases are considered in general in stochastic block modeling.

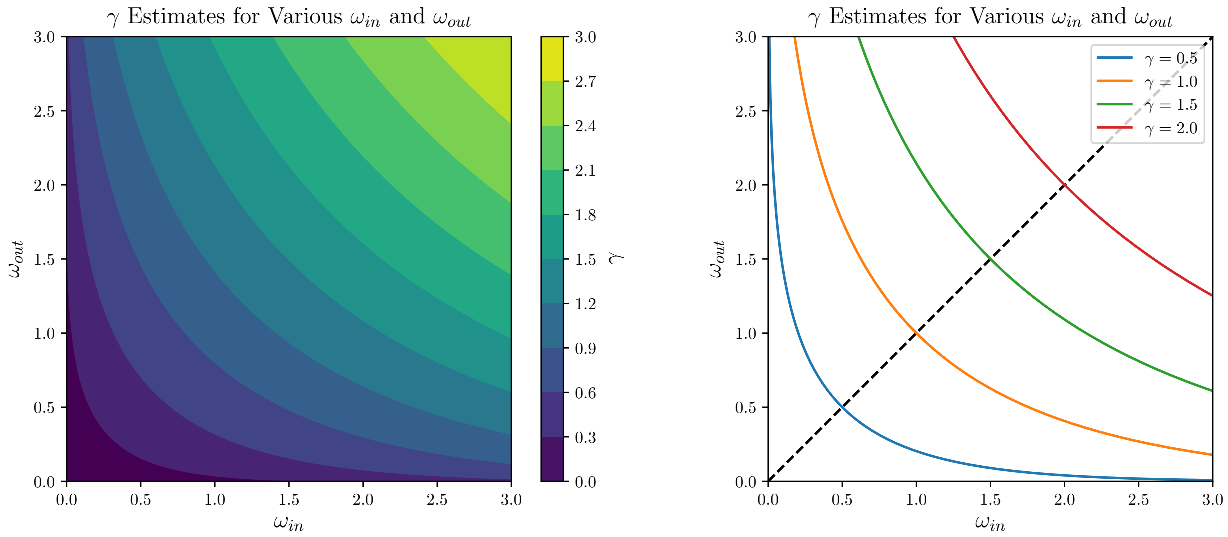

Figure S2. Left: The value of the γ estimate as ω_{in} and ω_{out} vary. Right: The possible $(\omega_{\text{in}}, \omega_{\text{out}})$ pairs associated with various choices of γ.

A.5 "Assumptions of Modularity"

Some variations on the stochastic block model include tunable parameters that allow for controlling the sizes of the ground truth groups [21]. However, as Newman notes, "the version [...] to which modularity maximization is equivalent, includes no such parameters, [...] which in effect means that a priori the sizes of all groups are the same and hence that modularity maximization implicitly prefers groups of uniform size" [1]. Moreover, when the number of communities is fixed and $\gamma > 0$, modularity maximization is always equivalent to the maximum likelihood fit of some degree-corrected planted partition SBM — for arbitrary $\gamma' > 0$ there are an infinite number of choices for ω_{in} and ω_{out} that will make the estimated γ equal to γ' in Equation S7 (see section A.6). Summarizing:

- Modularity maximization "assumes" that all communities in a network are "statistically similar" (in the equivalence to a planted partition, all communities share the same intra-group and inter-group connection propensities ω_{in} and ω_{out}).

- Modularity maximization "assumes" that all communities in a network are the same size (since the SBM of interest includes no parameters controlling group size). This means that a change in the number of communities can make a large difference in the "assumed group size" (and thus, estimates for ω_{in} and ω_{out}).

- Modularity maximization "assumes" the expected degree sequence of the network is identical to that observed empirically in the given network (since the equivalence is to a degree-corrected SBM).

A.6 Mapping Between γ and Ω in the Equivalence

Unsurprisingly since the resolution parameter estimation

$$\gamma = \frac{\omega_{\text{in}} - \omega_{\text{out}}}{\ln \omega_{\text{in}} - \ln \omega_{\text{out}}}$$

maps a two-dimensional space of $(\omega_{\text{in}}, \omega_{\text{out}})$ to the one dimensional space of γ in a smooth way — ignoring the singularities when $\omega_{\text{in}} = \omega_{\text{out}}$, this function is infinitely differentiable when $\omega_{\text{in}}, \omega_{\text{out}} > 0$ — this map is not one-to-one. Indeed, for any choice of γ', there are an infinite number of $(\omega_{\text{in}}, \omega_{\text{out}})$ pairs that satisfy $\gamma = \gamma'$ in the above equation, as shown in Figure S2. This means that the equivalence between modularity maximization and SBM inference actually holds for many possible pairs of SBM parameters ω_{in} and ω_{out}. Indeed, given any desired choice of γ and one of the two parameters ω_{in} or ω_{out}, we can

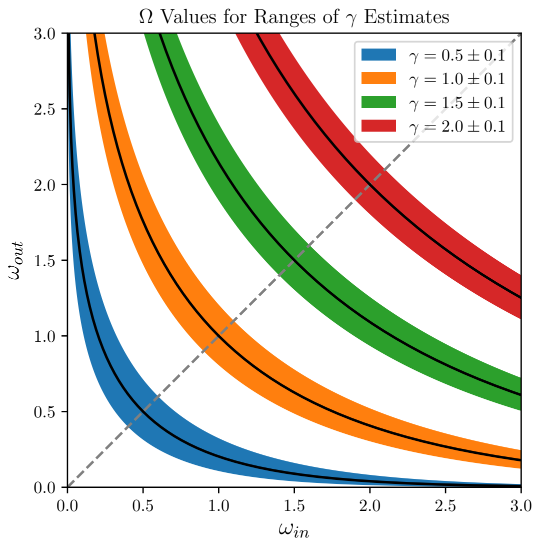

Figure S3. Regions of possible ω_{in}, ω_{out} values for various ranges of γ estimates.

compute the other parameter:

$$\omega_{\text{in}}(\gamma, \omega_{\text{out}}) = \begin{cases} -\gamma \cdot W_{-1}\left(\frac{-\omega_{\text{out}} \cdot e^{-\omega_{\text{out}}/\gamma}}{\gamma}\right), & \text{if } \omega_{\text{out}} < \gamma \\ -\gamma \cdot W_0\left(\frac{-\omega_{\text{out}} \cdot e^{-\omega_{\text{out}}/\gamma}}{\gamma}\right), & \text{if } \omega_{\text{out}} > \gamma \end{cases}$$

$$\omega_{\text{out}}(\gamma, \omega_{\text{in}}) = \begin{cases} -\gamma \cdot W_{-1}\left(\frac{-\omega_{\text{in}} \cdot e^{-\omega_{\text{in}}/\gamma}}{\gamma}\right), & \text{if } \omega_{\text{in}} < \gamma \\ -\gamma \cdot W_0\left(\frac{-\omega_{\text{in}} \cdot e^{-\omega_{\text{in}}/\gamma}}{\gamma}\right), & \text{if } \omega_{\text{in}} > \gamma \end{cases},$$

where W is the "Lambert W function", the function satisfying $W_0(xe^x) = x$ for $x \geq -1$ and $W_{-1}(xe^x) = x$ for $x \leq -1$.

Note that these ω_{in} and ω_{out} functions are continuous as seen in Figure S2 – the "principal branch" W_0 meets the "lower branch" W_{-1} when the argument is $-e^{-1}$, in which case $\omega_{\text{in}} = \omega_{\text{out}} = \gamma$ (though of course, the γ estimate is actually undefined here, but this matches the limiting behavior as $\omega_{\text{in}} \to \gamma$ and $\omega_{\text{out}} \to \gamma$).

A.7 Mapping Ranges of γ to Possible Ω Parameters of Planted Partition SBMs

This mapping between γ and possible $(\omega_{\text{in}}, \omega_{\text{out}})$ values also allows one to map entire domains of dominance ("optimality") in the space of γ values to the corresponding region in the $(\omega_{\text{in}}, \omega_{\text{out}})$ plane. We show this in Figure S3. Indeed, when a partition has a domain of optimality given by $[\gamma_{\text{center}} - r, \gamma_{\text{center}} + r]$, it appears to be the case that

$$\begin{aligned} \text{area in "}\Omega\text{ space"} &\approx 13.1594725 \cdot r \cdot \gamma_{\text{center}} \\ &\propto (\text{area in "}\gamma\text{ space"}) \cdot \gamma_{\text{center}}. \end{aligned} \tag{S8}$$

That is, if a partition has a domain of optimality given by $\gamma_{\text{center}} \pm r$, the area of the corresponding region in the $\omega_{\text{in}}, \omega_{\text{out}}$ "planted partition parameters plane" (i.e. the area of the region for which the partition has a greater likelihood fit to an underlying SBM than other partitions of the same number of communities) scales in a manner proportional to $r \cdot \gamma_{\text{center}}$.

In this way, one could potentially set priorities for giving attention to different partitions by first rescaling domains of optimality (in γ) to represent the area in Ω space that the domain represents after passing through the equivalence, though it's unclear if this would actually be useful in practice. One could also correct for the possible Ω values as K varies (see Figure S30 in section J). Interestingly, this gives an alternate explanation for why the domains of optimality tend to get smaller and the stochasticity of modularity maximization procedures tends to increase as γ grows larger. As this occurs, the same area of possible $\omega_{\text{in}}, \omega_{\text{out}}$ SBM parameters takes up an ever-smaller range of the γ space.

A.8 The "Equivalence" When K is Not Fixed

As we've seen in section A.3, the equivalence between modularity optimization and SBM inference only holds fully when the number of blocks K is kept fixed. However, Pamfil et al. [2] used the equivalence (and its extensions) without fixing the

number of communities and, in line with their results, we have found that when the community structure is strong enough we can often detect stability under the resolution parameter estimation map even as K varies (see section C.3.4 and the examples in section F/section G).

To help explain this, consider comparing the full log-likelihood SBM fit to modularity (for simplicity, ignoring the self-loop terms),

$$\begin{aligned}
\ln P(\mathbf{A} \mid \Omega, \mathbf{g}) &= \frac{1}{2} \ln \left(\frac{\omega_{\text{in}}}{\omega_{\text{out}}}\right) \sum_{i,j} \left[A_{ij} - \frac{k_i k_j}{2m} \cdot \frac{\omega_{\text{in}} - \omega_{\text{out}}}{\ln \omega_{\text{in}} - \ln \omega_{\text{out}}}\right] \delta(g_i, g_j) \\
&\quad + m(\ln \omega_{\text{out}} - \omega_{\text{out}}) \\
\frac{1}{m} \cdot \ln P(\mathbf{A} \mid \Omega, \mathbf{g}) &= \ln \left(\frac{\omega_{\text{in}}}{\omega_{\text{out}}}\right) \cdot \frac{1}{2m} \sum_{i,j} \left[A_{ij} - \frac{k_i k_j}{2m} \cdot \frac{\omega_{\text{in}} - \omega_{\text{out}}}{\ln \omega_{\text{in}} - \ln \omega_{\text{out}}}\right] \delta(g_i, g_j) \\
&\quad + (\ln \omega_{\text{out}} - \omega_{\text{out}}) \,.
\end{aligned}$$

Then, when we take the resolution parameter to have the value from the equivalence $\gamma = (\omega_{\text{in}} - \omega_{\text{out}})/(\ln \omega_{\text{in}} - \ln \omega_{\text{out}})$, we have that

$$Q = \frac{\ln P(\mathbf{A} \mid \Omega, \mathbf{g}) + m\,(\omega_{\text{out}} - \ln \omega_{\text{out}})}{m \ln (\omega_{\text{in}}/\omega_{\text{out}})}, \tag{S9}$$

where we've rewritten $-(\ln \omega_{\text{out}} - \omega_{\text{out}})$ as $(\omega_{\text{out}} - \ln \omega_{\text{out}})$ to emphasize that this quantity is positive (in fact, $(x - \ln x) \geq 1$ for all positive x).

Of course, when K is held constant, we can repeatedly estimate ω_{in} and ω_{out} to determine γ and iterate until convergence. Indeed, recall that when a network is actually drawn from a planted partition of K blocks that this iteration should converge to the correct community structure in the limit of large node degrees. But when we allow the number of communities K to vary in modularity maximization, we similarly allow for ω_{in}, ω_{out} to vary in such a way that we can possibly converge to multiple correct ground truth values for ω_{in} and ω_{out}, each corresponding to an SBM with a different number of blocks and potentially different parameters. (We explicitly construct examples of such networks in section H.) If we have partitions with varying numbers of communities in which our estimates for ω_{in} and ω_{out} do not cause the $(\omega_{\text{out}} - \ln \omega_{\text{out}})$ and $\ln (\omega_{\text{in}}/\omega_{\text{out}})$ terms in Equation S9 to vary much, the equivalence still approximately holds. This can occur, for example, if the community structure for a certain number of blocks is so strong that the log-likelihood dominates the expression. However, if the estimates for ω_{in} and ω_{out} differ greatly as K varies (e.g. if number of communities changes rapidly as we will see in section F), this equivalence can easily break down as the optimization of modularity is biased towards manipulating the $(\omega_{\text{out}} - \ln \omega_{\text{out}})$ and $\ln (\omega_{\text{in}}/\omega_{\text{out}})$ terms more than the likelihood of the SBM fit.

Unfortunately, the precise relationship between our γ, ω_{in}, and ω_{out} estimates as K increases depends heavily on the topology of the network. Hence, it is difficult to make any truly general statements about the relationship between modularity optimization and SBM inference here.

B The Use of Newman's Parameter Estimation in Pamfil et al.

Pamfil et al. [2] generalized Newman's [1] original equivalence to several variants of "multilayer networks" in which a collection of interrelated networks are treated as individual "layers" in a larger, connected network data structure. In this section, we briefly describe this extension of Newman's duality to multilayer settings. We also demonstrate issues arising from Pamfil et al.'s use of modularity maximization heuristics that do not keep the number of communities fixed.

As usual, there are many different formulations of multilayer networks, but Pamfil et al.'s extension focuses on three particular types: "temporal", "multilevel", and "multiplex" networks. Under certain choices of resolution parameters, they show that a multilayer extension of modularity maximization is equivalent to a form statistical inference using multilayer stochastic block models.

B.1 Multilayer Modularity

Consider a set of T layers of $n \times n$ adjacency matrices $\mathbf{A}^t$, $1 \leq t \leq T$, each representing the same set of n nodes. Further introduce a set of interlayer couplings C^{st}, one for each pair of distinct layers $1 \leq s,t \leq T$ such that node j in layer s is connected to itself in layer r with weight C_j^{sr}. The goal of community detection in this setting is to determine group membership per node and per layer, i.e. we must find the group assignment g_i^s of node i in layer s. (In the original paper [22], all variables are indexed with subscripts, so the group assignment of node i in layer s is denoted g_{is}, but we choose to use the notation g_i^s of

Pamfil et al. [2] here to match the discussion in later sections.) Under this framework, Mucha et al. [22] derived an extension of modularity to multilayer settings:

$$Q = \frac{1}{2\mu} \sum_{ijsr} \left[\left(A^s_{ij} - \gamma_s \frac{k^s_i k^s_j}{2m_s} \right) \delta(s,r) + C^{sr}_j \delta(i,j) \right] \delta\left(g^s_i, g^r_j\right) , \tag{S10}$$

where k^s_i is the degree of node i in layer s, m_s is the number of edges in layer s, and $2\mu = \sum_{is}\left(k^s_i + \sum_r C^{sr}_i\right)$ is twice the sum of all the network's edge weights. (Again, in weighted networks, all notions of degree or counts are naturally replaced with strength and total weights.) Note that in this formulation, each layer has a different "intralayer resolution parameter" with the weighting of the null model in layer s being controlled by γ_s.

Mucha et al. [22] also introduced a parameter ω to control the importance of the interlayer couplings in Equation S10. Similar to the usage of γ_s, one could include this as a multiplicative factor on the C^{st}_j term, but the original formulation takes the equivalent approach of absorbing ω into the definition of the weights of C^{st}_j. For simplicity, the interlayer couplings in [22] were taken to be binary so that this resolution parameter ω appears as

$$C^{st}_j = \begin{cases} \omega, & \text{if node } j \text{ is connected between layers } s \text{ and } t \\ 0, & \text{otherwise} \end{cases} \tag{S11}$$

This particular choice is known as uniform (interslice) coupling [23] since the weights of the interlayer couplings are identical across all layers. Note also that while [22] only discusses the specific cases of what were referred to therein as "multislice" networks, the general methodology of combining the contributions from within layers with an additional interlayer contribution carries over seamlessly to the more general class of (what are since termed) multilayer networks.

B.2 Temporal Networks

Temporal networks are those in which each layer encodes interactions during some period or instance of time. For instantaneously labeled continuous time temporal network data, we assume that some temporal discretization is then performed to group the temporal events into a finite number of layers. Hence, the layers can be placed in chronological order and a larger graph can be created by connecting each node to its copy in the preceding and subsequent layers ("ordinal coupling" between layers). Other interlayer connection strategies exist, but when the literature refers to "temporal networks", it is often the case that interlayer connections are strictly ordinal [24].

B.2.1 Uniform Coupling

Consider a network with T layers where layer t, $1 \leq t \leq T$ has adjacency matrix $\mathbf{A}^t$. Then, where the interlayer edges between layers $t-1$ and t all have weight ω_t, this network has a "supra-adjacency matrix" given by

$$\mathbf{A} = \begin{bmatrix} \mathbf{A}^1 & \omega_2 \mathbf{I} & \mathbf{0} & \dots & \mathbf{0} \\ \mathbf{0} & \mathbf{A}^2 & \omega_3 \mathbf{I} & \dots & \mathbf{0} \\ \vdots & \vdots & \vdots & \ddots & \mathbf{0} \\ \mathbf{0} & \mathbf{0} & \mathbf{0} & \dots & \omega_t \mathbf{I} \\ \mathbf{0} & \mathbf{0} & \mathbf{0} & \dots & \mathbf{A}^t \end{bmatrix}$$

so that each block on the diagonal represents intralayer connections and each nonzero off-diagonal block represents connections between adjacent layers. (In this particular formulation the interlayer edges are directed so as to represent the flow of information forward in time, though for the purposes of modularity-based community detection this is equivalent to having half the weight in both directions since only the symmetric part of the supramodularity matrix contributes to the quality objective function). In this way, node i in layer t is represented in the supra-adjacency matrix by node-layer index $n(t-1)+i$.

We will initially restrict our focus to the uniform interlayer coupling case in [2] where $\omega_t = \omega$ for all layers t. Then, the setup of the underlying multilayer SBM is a fairly straightforward extension of Newman's strategy [1] for the duality in the monolayer case. As before, consider the intralayer connections in layer t given by $\mathbf{A}^t$ to be drawn from a degree-corrected, planted partition stochastic block model with K blocks. In this SBM, the within-community edge propensities are given by θ_{in} and between-community edge propensities are given by θ_{out}. Note that these edge propensities θ_{in} and θ_{out} are directly analogous to the values of ω_{in} and ω_{out} from our discussion of the monolayer duality in section A.3. However, ω is already commonly used to represent the interlayer coupling parameter in Equation S10 and Equation S11, so to avoid confusion in the multilayer case we adopt this $\theta_{\text{in}}, \theta_{\text{out}}$ notation from Pamfil et al. [2].

The underlying SBM model further assumes that labels are copied between layers with "copying probability" p. That is, the ground truth group assignment g^t_i of node i in layer t is copied from layer $t-1$ with probability p and is assigned randomly

according to a null distribution $\mathbb{P}_0$ with probability $1-p$. For the purposes of the equivalence here, this null distribution $\mathbb{P}_0$ is taken to be uniform across all possible community labels $1,\ldots,K$.

Under this model, consider a partition $\mathbf{g}$ of the multilayer network where g_i^t is the group membership of node i in layer t. Then, neglecting constants that do not affect optimization, and utilizing some simplifications required here that are particularly nontrivial (we direct the interested reader to the original description in [2] for the complete derivation), we can write out the posterior probability of $\mathbf{g}$ in the model as

$$\ln P(\mathbf{g} \mid \mathbf{A}, \theta_{\text{in}}, \theta_{\text{out}}, p, K) = \sum_{t=1}^{T}\sum_{i,j=1}^{N}\left(A_{ij}^t - \frac{\theta_{\text{in}}-\theta_{\text{out}}}{\ln\theta_{\text{in}}-\ln\theta_{\text{out}}}\cdot\frac{k_i^t k_j^t}{2m_t}\right)\delta\left(g_i^t, g_j^t\right) + \sum_{t=2}^{T}\sum_{i=1}^{N}\frac{\ln\left(1+\frac{p}{1-p}K\right)}{\ln\theta_{\text{in}}-\ln\theta_{\text{out}}}\delta\left(g_i^{t-1}, g_i^t\right),$$

where as before, k_i^t is the degree of node i in layer t and m_t is the number of edges in layer t. Note the resemblance to multilayer modularity — maximizing this expression is exactly the same as maximizing multilayer modularity

$$Q = \frac{1}{2\mu}\left[\sum_{t=1}^{T}\sum_{i,j=1}^{N}\left(A_{ij}^t - \gamma\frac{k_i^t k_j^t}{2m_t}\right)\delta\left(g_i^t, g_j^t\right) + \sum_{t=2}^{T}\sum_{i=1}^{N}\omega\delta\left(g_i^{t-1}, g_i^t\right)\right] \tag{S12}$$

when

$$\gamma = \frac{\theta_{\text{in}}-\theta_{\text{out}}}{\ln\theta_{\text{in}}-\ln\theta_{\text{out}}} \qquad \text{and} \qquad \omega = \frac{\ln\left(1+\frac{p}{1-p}K\right)}{\ln\theta_{\text{in}}-\ln\theta_{\text{out}}}, \tag{S13}$$

where we have rewritten Q from Equation S10 with uniform interlayer coupling and intralayer resolution parameters to make the equivalence more obvious. Hence, these γ and ω are the "correct values" of the intralayer resolution and interlayer coupling parameters (hereafter often just called "the resolution parameters") if we want multilayer modularity maximization to be equivalent to the maximum likelihood fit of the SBM considered here. As before, we will call the values in Equation S13 the "γ estimate" and "ω estimate" (together, "resolution parameter estimates") of a partition.

The values of θ_{in} and θ_{out} are estimated in much the same way as we estimated ω_{in} and ω_{out} in the monolayer case, with the added restriction that group memberships are considered per layer rather than in aggregate. That is, the community strengths in layer t are computed as $\kappa_r^t = \sum_i k_i^t \delta\left(g_i^t, r\right)$ and then normalized using the number of edges per layer in Equation S4 with $\sum_r (\kappa_r^t)^2/(2m_t)$. The copying probability p of labels from one layer to the next is similarly estimated using the observed frequency with which the group membership of node i persists across layers — i.e. we can estimate p by calculating the probability that $g_i^{t-1} = g_i^t$ over all layers $t = 2,\ldots,T$ and all nodes $i = 1,\ldots,N$. Then as in the monolayer case in section A.3, we can iteratively find "correct" values for (γ, ω) by maximizing modularity with the number of communities fixed, computing new estimates for γ and ω, and then repeating until convergence.

B.2.2 Non-Uniform Coupling

A similar result can be derived when considering non-uniform coupling where the intralayer SBM parameters θ_{in}^t, θ_{out}^t, number of communities per layer K_t, intralayer resolution parameters γ_t, interlayer resolution parameters ω_t, and copying probabilities p_t vary over the layers t. We defer that discussion to the original paper in [2], but for the sake of completeness, the equivalence in this case is to multilayer modularity with added intralayer weights β_t (cf. Equation S12)

$$Q = \frac{1}{2\mu}\left[\sum_{t=1}^{T}\beta_t\sum_{i,j=1}^{N}\left(A_{ij}^t - \gamma_t\cdot\frac{k_i^t k_j^t}{2m_t}\right)\delta\left(g_i^t, g_j^t\right) + \sum_{t=2}^{T}\sum_{i=1}^{N}\omega_t\cdot\delta\left(g_i^{t-1}, g_i^t\right)\right]$$

and the correct choices of γ_t, ω_t, and β_t are given by

$$\gamma_t = \frac{\theta_{\text{in}}^t-\theta_{\text{out}}^t}{\ln\theta_{\text{in}}^t-\ln\theta_{\text{out}}^t}, \qquad \omega_t = \frac{\ln\left(1+\frac{p_t}{1-p_t}K_t\right)}{\langle\ln\theta_{\text{in}}^t-\ln\theta_{\text{out}}^t\rangle_t}, \qquad \beta_t = \frac{\ln\theta_{\text{in}}^t-\ln\theta_{\text{out}}^t}{\langle\ln\theta_{\text{in}}^t-\ln\theta_{\text{out}}^t\rangle_t} \tag{S14}$$

where $\langle\cdot\rangle_t$ denotes a mean across all layers.

B.3 Multilevel Networks

A multilevel network is an extension of temporal networks in which the interactions between layers encode a hierarchy of relationships [25]. For example, individuals may work in a department of a company in a certain sector of the economy and this inclusion could be modeled as interlayer edges between four different layers (individual, department, company, and sector of economy). In contrast to temporal networks, these interlayer relationships need not represent an ordinal flow and nodes connected across layers do not necessarily represent the same entity. Importantly, the hierarchy of relationships admits a natural ordering of layers and [2] show that the copying probability model from temporal networks in section B.2 can be adapted for use here. In particular, the group memberships need to be copied from parent to child across layers and we need to ensure that at most a single connection exists to each node as the labels are copied from layer to layer. Other than this tweak to copying probabilities, the derivation is very similar to temporal networks. Indeed, the correct choices for resolution parameters match exactly with the temporal case! For uniform coupling, the "correct γ and ω estimates" are given in Equation S13 and for non-uniform coupling, the "correct parameters" are as in Equation S14.

B.4 Multiplex Networks

Multiplex networks are those in which the edges between nodes are categorized by type. Then, the edges of each type are embedded into layers of a larger network (that is, a given layer represents the edges of a single type) [24]. Here, the interlayer interactions are often taken to be fully connected. The precise details of the derivations for the multiplex case in [2] are complicated and we will only briefly summarize the methods and results here. Once again, we direct the interested reader to the full description in the original paper [2].

Unfortunately, because these networks do not admit any natural ordering of layers, the copying probability approach from the temporal and multilevel network models does not directly apply here. Instead, the approach taken in Pamfil et al. [2] is essentially to consider label copying over all possible orderings of the layers. Indeed when the network has T layers, the derived expression for the optimal interlayer coupling ω_{st} between layers s and t requires a sum over the symmetric group S_{T-1} in which each summand individually involves a product over all of the other $T-1$ layers. Needless to say, this sum has $(T-1)!$ terms and is only tractable for networks with an extremely small number of layers, though it is not uncommon to have only a few layers in some real-world settings. However, simplifications can be made when the copying probabilities p_{st} between all pairs of layers $s,t \in \{1,\ldots,T\}$ are taken to have the same value $p_{st} = p$ and all possible orderings of the layers are sampled with equal probability $1/(T!)$. In this case, the problematic sum over S_{T-1} can be approximated to obtain

$$\omega = \frac{\ln\left(1 + \frac{p}{1-p}K\right)}{T\langle \ln\theta_{\text{in}} - \ln\theta_{\text{out}}\rangle_t}$$

as the optimal value for the interlayer resolution parameter in uniform multiplex networks. Note that this estimate is equivalent to dividing the ω estimate from temporal and multilevel models by the number of layers T (cf. Equation S14). Informally, this scaling by a factor of T corrects for the T-fold increase in the number of interlayer edges compared to temporal networks. That is, due to the ordinal coupling in temporal networks with T layers and N nodes per layer, such networks have $N(T-1)$ interlayer edges whereas fully interlayer connected multiplex networks have $NT(T-1)$ directed interlayer edges.

Even so, it is not straightforward to estimate p in the multiplex case since one must consider all permutations of the layers individually. The estimate of persistence of group labels must be considered across all pairs of layers

$$P(g_i^s = g_i^t) \approx \frac{1}{NT(T-1)} \sum_{i=1}^{N} \sum_{t=1}^{T} \sum_{s \neq t} \delta\left(g_i^s, g_i^t\right) \tag{S15}$$

and it can be shown that the expected probability of $g_i^s = g_i^t$ under all possible permutations of layers is given by

$$P(g_i^s = g_i^t) = \frac{2\left(1 - \frac{1}{K}\right)}{T(T-1)} \sum_{n=1}^{T-1} p^n (T-n) + \frac{1}{K}. \tag{S16}$$

Hence, we can estimate p here by equating the right-hand sides of Equation S15 and Equation S16 and solving the resulting polynomial root-finding problem. This calculation only works when all copying probabilities take the same value $p_{st} = p$ as before. A tractable method for accurately approximating p_{st} when this is not the case remains an open problem.

B.5 Differences with Newman's Scheme for Determining the "Correct Values" of Resolution Parameters

Importantly in Newman's equivalence [1] and the extensions described in this section, the duality between modularity maximization and maximum likelihood to a stochastic block model holds fully when the number of communities, K, is fixed. In iterating until the resolution parameter estimates converge, Pamfil et al. [2] maximizes modularity using the GenLouvain

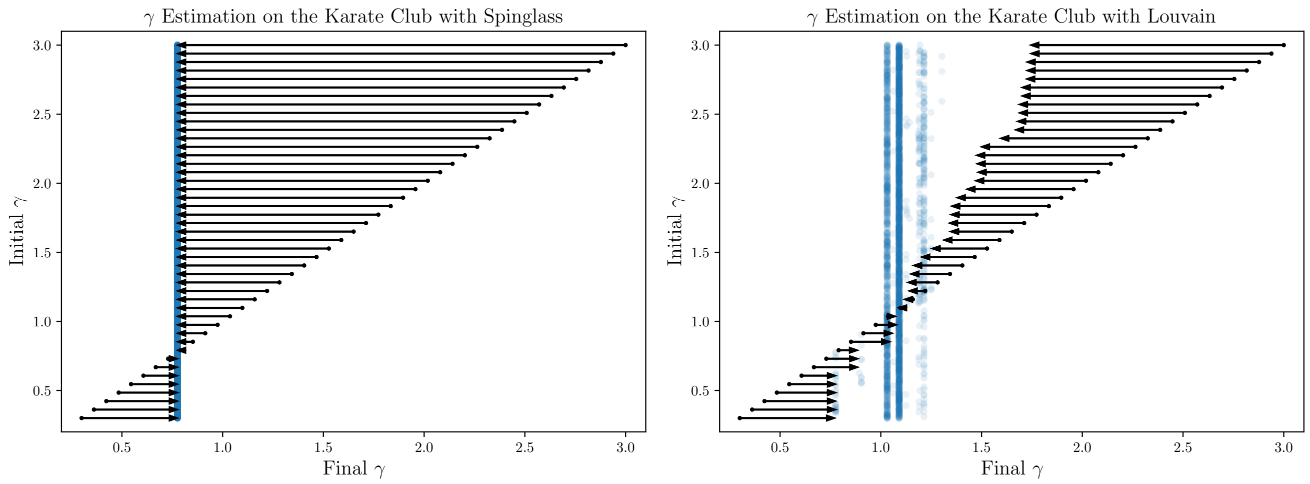

Figure S4. Behavior of the iteration to determine "correct" values for the resolution parameter γ. Arrows show the average movement (over 100 trials) induced in γ space where the base of the tail lies at the initialization γ and the head of the tail lies at the resulting γ estimate after one iteration. Pairs of (final converged γ, initialization γ) obtained across many runs as shown in blue. Left: Behavior of the scheme when using the spin glass algorithm [10] restricted to finding $K = 2$ communities. Right: Behavior of the scheme when using the Louvain algorithm [16], which does not fix K.

algorithm [26]. Notably, this algorithm is based on the Louvain procedure of [16] and thus does not keep K fixed. Moreover, this behavior might be particularly preferred in multilayer settings where it is much less common to know *a priori* how many communities is appropriate to the data set. However, this flexibility in letting K vary poses three primary issues in practice.

1. Due to a significantly larger search space, modularity maximization algorithms that allow K to vary tend to be much more stochastic than those that fix K. This can cause problems with the convergence of the iterative resolution parameter estimation.

2. When not fixing K, we will discover multiple "correct" values for the resolution parameters far more frequently than when K is fixed, depending on the initialization values (e.g. when two fixed points of the iterative scheme correspond to different K). Indeed, one of the nice guarantees of Newman's original procedure was that it would eventually "converge to the correct value of γ (and the correct community structure) for networks that are actually generated from a planted partition model (in the limit of large node degrees)" [1].

3. In regions where modularity maximization heuristics do not consistently return partitions with the same number of communities, the resulting resolution parameter estimates vary wildly and can lead to convergence problems. In fact, this can "hide" significant partitions when heuristics do not effectively estimate K. One such example is discussed in section F.2 where Louvain fails to find the desired number of communities near the ground truth resolution parameter estimate.

Pamfil et al. demonstrates that allowing K to remain unspecified with their iterations (i.e. determining K by the community detection heuristics at each point in the parameter space) appears to work well in some situations. Depending on the precise details of the implementation, however, this additional flexibility interacts with the possible stochasticity arising from the optimization heuristics, as becomes apparent even on what is perhaps the most overstudied community detection example: Zachary's karate club [5]. This network describes the social relationships between individuals in a university karate club shortly before a disagreement between an administrator and the instructor split the group in two. First, we analyze the results of the iterative scheme to find γ estimates when using two different modularity maximization algorithms: the Louvain algorithm [16] (which does not restrict the number of communities K), as implemented by Vincent Traag [8], and the spin glass algorithm of Reichardt and Bornholdt [10] as implemented in igraph [7] (where we restrict $K = 2$ as in Newman's paper [1]). The behavior of γ estimation under these two algorithms is shown in Figure S4.

First, note that the stochasticity is greatly increased when using Louvain to maximize modularity. Newman [1] found that his iterative scheme consistently converged to finding an optimal estimate $\gamma \approx 0.78$ where a 2-community partition has highest modularity. This matches the behavior we see when using the spin glass algorithm where convergence to $\gamma \approx 0.78$ occurs after the first iteration of the scheme, regardless of initialization γ. However, when using Louvain, the scheme

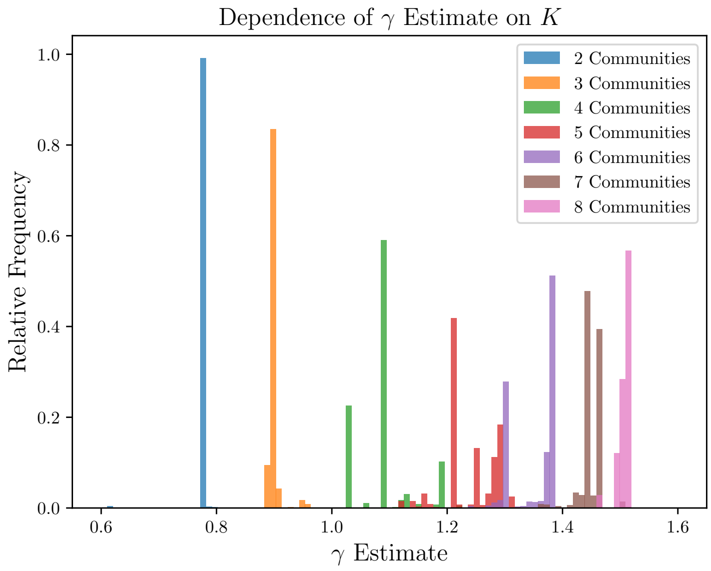

Figure S5. Frequency of γ estimates on the Karate Club from 1,000,000 runs of Louvain across a uniform grid of $\gamma \in [0.0, 2.0]$. Relative frequencies are given in terms of all observed partitions with the same number of communities K.

most frequently converges to an estimate of $1.0 \leq \gamma \leq 1.1$, where a 4-community partition has highest modularity. Indeed, convergence to $\gamma \approx 0.78$ only occurs when the iterative procedure is initialized with a very small γ value. Even then, the algorithm most commonly returns an estimate of $1.0 \leq \gamma \leq 1.1$. Second, while the procedure using the spin glass algorithm always returns the same final resolution parameter estimate, the procedure using Louvain finds multiple "correct values". Most frequently, a 4-community partition with $1.0 \leq \gamma \leq 1.1$ is returned, but the 2-community partition with $\gamma \approx 0.78$ from Newman's experiments [1] and a 3-community partition with $\gamma \approx 0.9$ are occasionally found as well.

Indeed, there is a strong dependence of a partition's γ estimate and its number of communities, as we show on the karate club in Figure S5. This dependence contributes to the pseudo-random inconsistency in outcomes when using Louvain here in that a change in the number of communities found in a partition typically leads to a large change in the corresponding γ estimate. For example, if Louvain "chooses" at random between a 2-community or 3-community partition, the iterative scheme will randomly continue with $\gamma \approx 0.78$ or $\gamma \approx 0.9$, respectively.

In hindsight, this behavior is unsurprising — the duality between modularity optimization and stochastic block model inference depends on estimates of the SBM parameters, which may differ greatly when the number of blocks is changed. Hence, in general, the results of resolution parameter estimation will strongly depend on the number of communities returned by the modularity maximization heuristic of choice. We discuss the duality's dependence on K more in section A.3 and section A.8. We will analyze models in which multiple meaningful ground-truth partitions exist with different number of blocks K in section H. We additionally consider SBMs with a hierarchy of latent community structure and LFR networks whose degree and community size sequences follow power laws in section I.

C Summary of CHAMP and its Use in Our Method

In [3], Weir et al. proposed the CHAMP (Convex Hull of Admissible Partitions) algorithm to post-process sets of network partitions in order to identify regions of modularity optimization. Given an input set, CHAMP identifies domains of the resolution parameter space for which each partition has the largest modularity relative to the input set. In this section, we describe the CHAMP algorithm and its importance in our pruning procedure.

C.1 Terminology

Consider a network and a set of partitions $\Sigma = \{\sigma_1, \ldots, \sigma_s\}$ of this network. When modularity has resolution parameters $\gamma_1, \ldots, \gamma_k$, we say

- A partition $\sigma \in \Sigma$ is "dominant" or "optimal" at $(\gamma_1, \ldots, \gamma_k)$ if $Q_\sigma(\gamma_1, \ldots, \gamma_k) \geq Q_{\sigma'}(\gamma_1, \ldots, \gamma_k)$ for all $\sigma' \in \Sigma$.
- A partition $\sigma \in \Sigma$ is "somewhere dominant" or "somewhere optimal" if it is optimal for some choice of $(\gamma_1, \ldots, \gamma_k)$. Otherwise, we say σ is "nowhere optimal". In the original paper [3], Weir et al. describe the "admissible" subset of

partitions as the subset of those that are somewhere optimal.

- The "domain of optimality" or "domain of dominance" of $\sigma \in \Sigma$ is the convex polytope of values $(\gamma_1, \ldots, \gamma_k)$ for which σ is dominant. This domain is nonempty if and only if σ is somewhere optimal.

For example, the domain of optimality of a single-layer partition is a (potentially empty) contiguous range of γ values. Similarly, the domain of optimality of a multilayer partition is a convex polygon in the (γ, ω) plane.

C.2 The CHAMP Algorithm

When maximizing modularity, it is usually not obvious which values of the resolution parameter γ should be used. As such, one typically runs modularity maximization heuristics multiple times at several values of γ and then searches for community structure in the returned partitions that is meaningful, robust to multiple runs and parameter values, and/or significant in some sense. To aid in this search, Weir et al. [3] developed the CHAMP (Convex Hull of Admissible Partitions) algorithm which takes a set of partitions and identifies those that have a modularity score higher than all the other partitions for some nonempty range of parameter values.

First, note that for a fixed partition σ, the modularity is linear in γ. Ignoring the leading constant of $1/(2m)$, we can rewrite modularity as

$$Q_\sigma(\gamma) = \sum_{i,j} [A_{ij} - \gamma P_{ij}]\, \delta(g_{i\sigma}, g_{j\sigma})$$
$$Q_\sigma(\gamma) = \left[\sum_{i,j} A_{ij} \delta(g_{i\sigma}, g_{j\sigma})\right] - \gamma \left[\sum_{i,j} P_{ij} \delta(g_{i\sigma}, g_{j\sigma})\right]$$
$$Q_\sigma(\gamma) = \hat{A}_\sigma - \gamma \hat{P}_\sigma$$

where the "null model" for an undirected network is typically taken to be the Newman-Girvan null model, $P_{ij} = \frac{k_i k_j}{2m}$, and we have made the dependence on the partition σ explicit in the subscripts, i.e., $g_{i\sigma}$. In this way, each partition of a network corresponds to a line in (γ, Q) space. Then, we can obtain the set of somewhere dominant partitions and their domains of optimality by finding the border of the region that lies above all partition lines in the (γ, Q) space. This is known as the halfspace intersection problem and there are efficient solutions from the field of computational geometry for computing this region. In particular, CHAMP's implementation at [4] uses the software package Qhull which "may be used for 2-d up to 8-d", according to the authors on qhull.org [27]. Crucially, for single-layer and the simplest multilayer network analyses (where only 2 and 3 dimensions are needed, respectively), the worst case running time of Qhull is $O(n \log n)$ for n input partitions. This method is very quick for practical resolution parameter spaces where the dimensionality is small. Indeed, taking the intersection of millions of random halfspaces in $\mathbb{R}^3$ takes only a few seconds. We visualize the CHAMP procedure in Figure S6.

A similar result holds for multilayer networks where we introduce an additional interlayer connections (supra-) matrix C and interlayer resolution/coupling parameter ω. In this case, ignoring multiplicative constants modularity can be written in simplified notation with i and j indexing node-layers of the appropriate (supra-) matrices as

$$\begin{aligned} Q_\sigma(\gamma, \omega) &= \sum_{i,j} [A_{ij} - \gamma P_{ij} + \omega C_{ij}]\, \delta(g_{i\sigma}, g_{j\sigma}) \\ &= \hat{A}_\sigma - \gamma \hat{P}_\sigma + \omega \hat{C}_\sigma\,, \end{aligned}$$

where $\hat{A}_\sigma$, $\hat{P}_\sigma$, and $\hat{C}_\sigma$ are the within-community sums of A_{ij}, P_{ij} and C_{ij} respectively. Importantly, this is linear in both resolution parameters γ and ω.

In other words, each partition of a single-layer network is represented by a line in (γ, Q) space and each partition of a multilayer network is represented by a plane in (γ, ω, Q) space. In this way, we can consider the quality of a partition across many different resolution parameter values.

This generalizes in a straightforward way, though at the cost of higher dimensionality, to cases in which we have k resolution/coupling parameters. If we have a form of modularity written as

$$\begin{aligned} Q_\sigma(\gamma_1, \ldots, \gamma_k) &= \sum_{i,j} \left[A_{ij} - \gamma_1 P_{1,i,j} - \gamma_2 P_{2,i,j} - \cdots - \gamma_k P_{k,i,j}\right] \delta(c_{i,\sigma}, c_{j,\sigma}) \\ &= \hat{A}_\sigma - \gamma_1 \hat{P}_{1,\sigma} - \gamma_2 \hat{P}_{2,\sigma} - \cdots - \gamma_k \hat{P}_{k,\sigma}\,, \end{aligned}$$

then each partition is represented by a hyperplane in $(\gamma_1, \ldots, \gamma_k, Q)$ space.

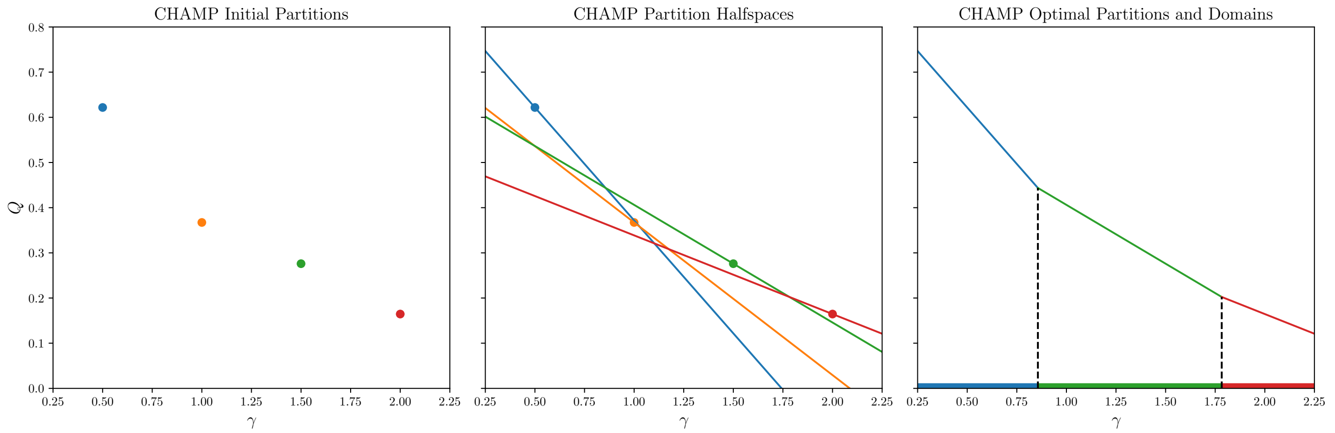

Figure S6. Summary of the CHAMP algorithm for single-layer networks with one resolution parameter γ. Left: Partitions are sampled in (γ, Q) space, using a modularity maximization heuristic. Center: The partitions are considered as lines or, equivalently, halfspaces in (γ, Q) space. Right: The intersection of the halfspaces is obtained and the resulting facets are projected into the parameter space to obtain the set of somewhere dominant partitions and their domains of optimality.

Primarily, CHAMP provides a method for pruning a large number of partitions of a network (potentially taken from a vast range of the resolution parameter space and from various computational heuristics) into the small subset of "admissible" partitions that are somewhere dominant. Importantly, each partition is treated as a hyperplane and not just as a single point in the resolution parameter space to take full advantage of the input partitions. In particular, if a partition were returned by a computational heuristic running at resolution parameter γ, it is possible for that partition to be dominant for many values $\gamma' \neq \gamma$. Moreover, we have repeatedly found cases in our examples where the most common partitions returned by Louvain at a point in the parameter space are less optimal than a different partition obtained at a different but nearby point. In the original CHAMP paper, the pruned subsets were often observed to be several orders of magnitude smaller than the full set of partitions — the original paper focused on examples where "pruned subsets of admissible partitions [were] 20-to-1785 times smaller than the sets of unique partitions [...] input into CHAMP" [3] — though this obviously depends on the size and variance in quality of the set of partitions to be pruned.

C.3 Benefits of Using CHAMP in Our Method

Note that by passing through Newman's duality from [1], restricting focus to somewhere dominant partitions is equivalent to only considering those partitions that have a maximum likelihood fit with respect to the input partitions to a (planted partition, degree corrected) stochastic block model as the parameters vary. In general, there may be some concerns about potentially removing important partitions through pruning with CHAMP. However, the quality of partitions considered for the iterative resolution parameter estimation are directly related to the likelihood of the underlying fit to an SBM and thus, the quality of the resulting resolution parameter estimates. Hence, we believe that it is reasonable to ignore the nowhere dominant partitions here. Crucially, this gives us (possibly empty) domains for each partition, making the resolution parameter estimation completely deterministic and discrete after selection of the partitions to be input into CHAMP. Then, a partition whose resolution parameter estimate lies within its own domain of optimality is analogous to a fixed point of the iterative estimation procedures from Newman [1] and Pamfil et al. [2]. In this case, we will say that the partition is "stable under the resolution parameter estimation map" with respect to the pruned subset of partitions or (for the purposes of brevity) that the partition is "stable". This is the way in which stable partitions are "significant" from the perspective of stochastic block model inference. Such a partition has greater quality than all other partitions of interest at the value of the resolution parameter where modularity maximization becomes equivalent to (planted partition, degree corrected) stochastic block model inference.

We close this section with discussion of the multiple benefits of using CHAMP here in this manner.

C.3.1 Suppression of Stochasticity in Parameter Estimation

First, by suppressing the stochasticity of modularity maximization heuristics during iterative resolution parameter estimation, we no longer have to worry about issues of randomness while determining stability or fixed points. Of course, we have to pay for this by actually finding a set of partitions to prune in the first place, but testing a partition for stability now becomes as easy as finding self loops in a graph.

C.3.2 Statistically Principled When Restricting the Number of Communities

Second, we may easily choose between keeping the number of communities K fixed or letting it vary. Recall that the full statistical grounding of Newman's [1] scheme relies on K being held fixed whereas Pamfil et al. [2] allow K to vary. Prior to the start of the CHAMP step here, it is a trivial substep to restrict our focus to only those partitions with a specified number of communities. Of course, we obviously cannot guarantee that the results will be exactly the same as with heuristics that keep the number of communities fixed within a maximization algorithm, since we're ultimately at the mercy of our community detection algorithm of choice. Then again, this approach allows one to use effectively any algorithm of choice and then restrict K at the start of the CHAMP step. However, if the input set of partitions contains enough reasonably high quality K-community partitions, then the behavior of our scheme should not differ greatly from Newman's original proposal. Indeed, in practice when networks have strong K-block structure, we have found that the Louvain algorithm detects partitions with K communities for fairly large ranges of the resolution parameters. That said, however, given the tendency of modularity to favor well-balanced partitions, it is certainly possible that heuristics allowing K to vary can miss smaller dense subgraphs that might have been uncovered by a fixed-K heuristic run at values of γ high enough where the varying-K heuristics only return partitions with larger numbers of communities. Ultimately, the key limitation of CHAMP here is that it does not detect new partitions; rather, it only prunes the set of partitions that it receives as input.

C.3.3 Handling Networks with Multiple "Correct" Resolution Parameter Values

Third, the reduction to a deterministic map allows us to more easily handle networks in which there are multiple "correct" values for the resolution parameter. For example, a network with significant community structure at multiple different scales may have one "correct" value of γ that corresponds to a strong split into communities at one scale and a higher value of γ that corresponds to densely connected subcommunities within the larger ones. We have seen this occur in practice and have explicitly constructed models (see section H) in which multiple partitions of a network are simultaneously stable under the parameter estimation map.

C.3.4 When Communities are Strong Enough, K Does Not Need to be Fixed

Fourth, strong community structure may be stable under the map even when the number of communities is not fixed (as in Pamfil et al.'s proposal [2]) and, importantly, this notion of stability as a fixed point under the map with varying K is strictly stronger than that for the corresponding maps with fixed values of K. That is, by fixing the number of communities being considered and thus decreasing the number of partitions of interest, we only increase the sizes of the domains of optimality. Hence, if a partition is stable when considering any number of communities, it will also be stable when only considering those partitions that share its number of communities. In particular, stability of a partition σ with respect to a full set of partitions Σ necessarily implies stability of σ with respect to any subset of Σ that includes σ.

D More Results on Zachary's Karate Club

In section B.5, we discussed some peculiar behavior arising from Pamfil et al.'s choice to allow the number of communities to remain unspecified during modularity maximization. In that discussion, we used the karate club network of Zachary [5] as a simple example. Recall that this network describes the social relationships in a university karate club shortly before a disagreement split the club in half. Here, we present some additional results on that same network.

We obtained 10,000,000 partitions of this network by running the Louvain algorithm (as implemented by Vincent Traag in [8]) across a uniformly spaced grid for $\gamma \in [0.0, 2.0]$ and then used CHAMP [3] to prune this to the subset of partitions that are somewhere dominant. Among these partitions were 539 unique partitions (including the single-community partition which is optimal for small enough values of γ) and the pruned subset from CHAMP has only 9 partitions. Some details of these partitions are shown in Table S1 and their domains of optimality and γ estimates are shown in Figure S7.

We stress that we are running the Louvain algorithm an extremely large number of times only for the purposes of being exhaustive, though 10 million runs on this small of a network takes fewer than 5 minutes on the desktop computer we used. The results for this network are qualitatively similar when the number of Louvain runs are as low as 100 to 1000, with the stable partitions of interest in Figure S8 generally appearing with as few as a dozen input partitions. In practice, you would run community detection heuristics for only as long as your situation allows, and of course the computational abilities differ greatly between those using a laptop and those with access to large compute clusters or AWS instances. Fortunately, the number of partitions in CHAMP's pruned subset appears to converge very rapidly in practice, so while the quality of our method's results may improve as the number of input partitions increases, it is in no way mandatory to run modularity maximization heuristics for an inordinate amount of time. Indeed, CHAMP will prune a set of partitions, regardless of how small or large the set may be.

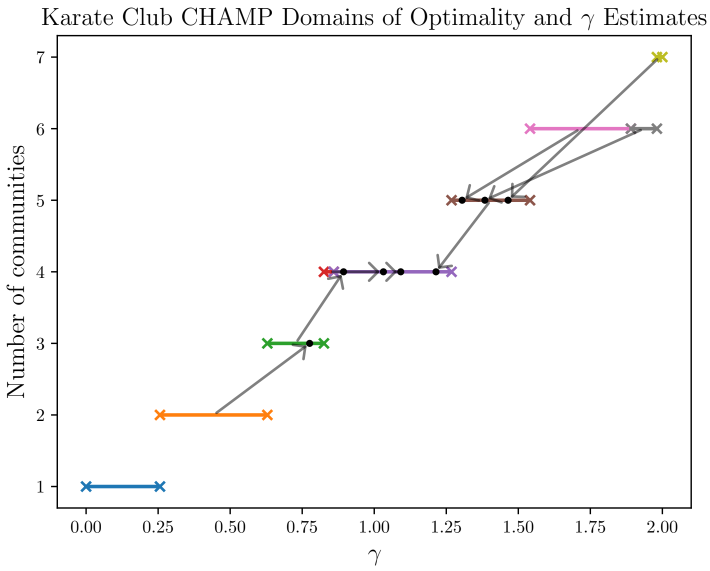

Figure S7. The domains of optimality and associated γ estimates for the 9 partitions of the karate club in the pruned subset from CHAMP.

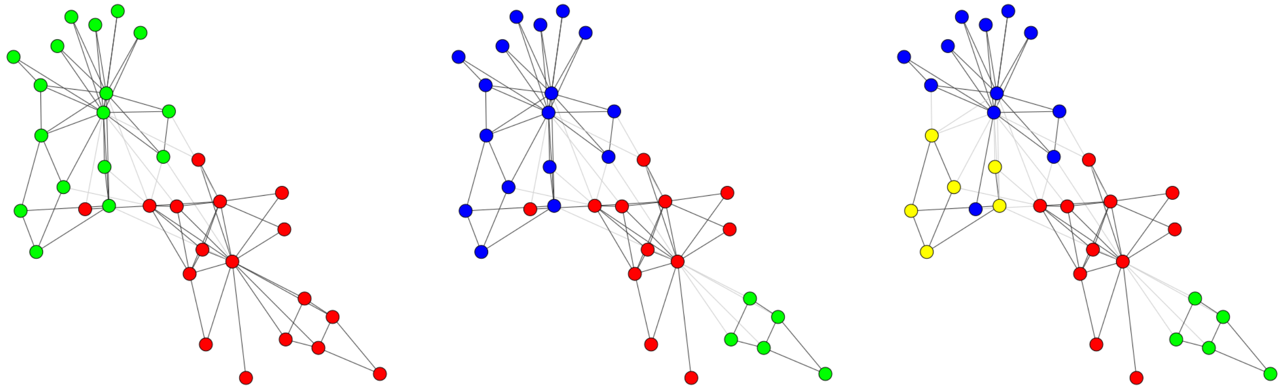

Figure S8. Force-directed layouts of Zachary's karate club network generated in igraph [7] with node colors for the stable partitions from the CHAMP subsets when only considering partitions of 2, 3, and 4 communities, respectively (left to right).

Number of communities K	2	3	4	5	6	7	8
Number of unique partitions	10	23	109	184	156	49	7
Number of unique partitions in CHAMP's pruned subset	1	1	2	1	2	1	0
Number of unique partitions in CHAMP's pruned subset when only considering partitions with K communities	2	3	3	4	4	3	2

Table S1. A description of the unique partitions returned by running the Louvain heuristic 10 million times on Zachary's karate club network and the pruned subsets from running CHAMP on these partitions.

Note that when the number of communities is left unrestricted, there is exactly one stable partition in CHAMP's pruned subset whose γ estimate lies within its domain of optimality: namely, one of the two 4-community partitions in Figure S7, which was also the partition that Pamfil et al.'s iterative procedure most frequently converged to in Figure S4.

When the number of communities K is restricted prior to post-processing with CHAMP, we find exactly one stable partition per choice of $K = 2, 3, \ldots, 8$. The stable 2-, 3-, and 4-community partitions are visualized in Figure S8. The 2-community stable partition closely matches the true splitting of the karate club and the 4-community stable partition is the same as the one obtained allowing K to vary. (Recall from section C.3.4 that a stable partition σ with respect to a set of partitions Σ will also be

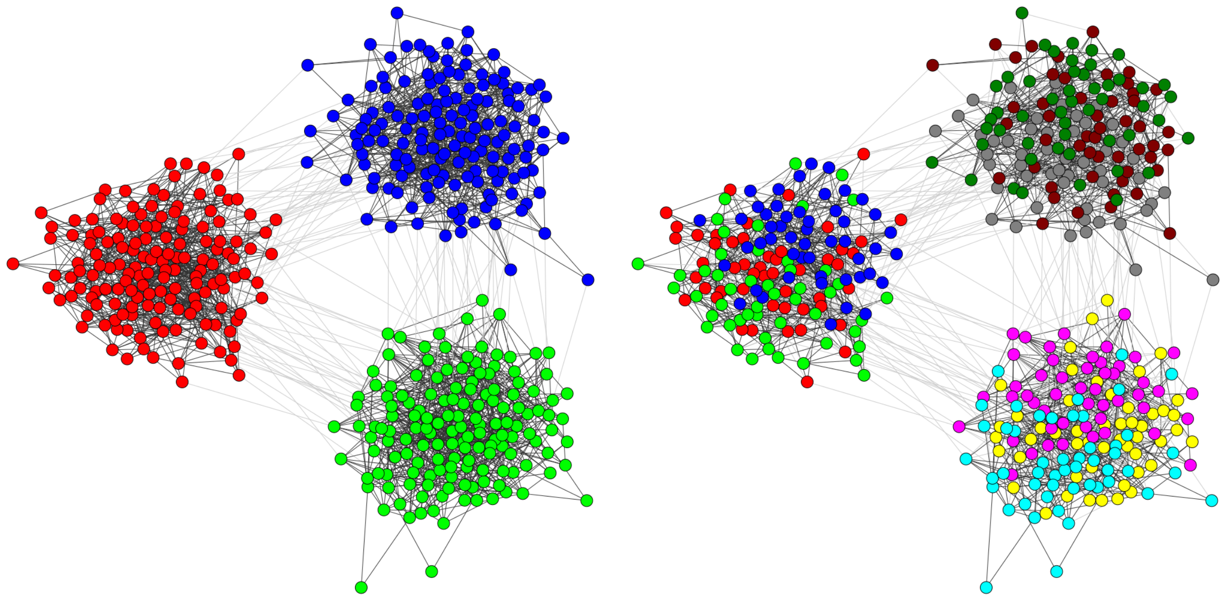

Figure S9. Force-directed layout of an example realization of the hierarchical stochastic block model considered here. Both panels here are of the same layout, generated by `layout_fruchterman_reingold` in igraph [7], with node colors indicating the ground truth partitions into (Left) 3 communities and (Right) 9 communities. We particularly note that the split here into 9 communities at these parameters is visually less obvious (except by the node colors in the right panel) compared to that into 3 communities.

stable with respect to any subset of Σ that includes σ. Hence, since this 4-community partition was stable in Figure S7, it is also stable when we restrict consideration to partitions with exactly 4 communities.)

E Hierarchical Stochastic Block Models

With this synthetic model we further evaluate some of the consequences of running our pruning strategy with the number of communities allowed to vary during modularity maximization. In particular, we run our pruning method on realizations drawn from a stochastic block model with a latent hierarchy of community structure. We are interested in determining whether the method will detect both of the ground truth partitions when the number of communities is not fixed in modularity maximization. Specifically, we generate graphs from an SBM with three large communities and nine small communities where each large community is made out of three smaller ones. We use a simple SBM with the matrix of block connection probabilities given by

$$\left[\begin{array}{ccc|ccc|ccc} p_1 & p_2 & p_2 & p_3 & p_3 & p_3 & p_3 & p_3 & p_3 \\ p_2 & p_1 & p_2 & p_3 & p_3 & p_3 & p_3 & p_3 & p_3 \\ p_2 & p_2 & p_1 & p_3 & p_3 & p_3 & p_3 & p_3 & p_3 \\ \hline p_3 & p_3 & p_3 & p_1 & p_2 & p_2 & p_3 & p_3 & p_3 \\ p_3 & p_3 & p_3 & p_2 & p_1 & p_2 & p_3 & p_3 & p_3 \\ p_3 & p_3 & p_3 & p_2 & p_2 & p_1 & p_3 & p_3 & p_3 \\ \hline p_3 & p_3 & p_3 & p_3 & p_3 & p_3 & p_1 & p_2 & p_2 \\ p_3 & p_3 & p_3 & p_3 & p_3 & p_3 & p_2 & p_1 & p_2 \\ p_3 & p_3 & p_3 & p_3 & p_3 & p_3 & p_2 & p_2 & p_1 \end{array}\right], \quad p_1 = \frac{6}{N/9}, \quad p_2 = \frac{3}{2N/9}, \quad p_3 = \frac{0.5}{2N/3} \tag{S17}$$

so that each node is expected to have approximately 0.5 edges connected outside of its large community, 3 edges connected within its large community but outside its small subcommunity, and an additional 6 edges connected within its small subcommunity. In our experiments here, we choose $N = 450$. An example graph realization is visualized in Figure S9.

We ran our pruning technique on realizations drawn from this stochastic block model by running Louvain 1000 times on each realization on a uniform grid of $\gamma \in [0,3]$. We recorded the number of communities in the stable partitions returned, collecting the results in Figure S10. Importantly, in these experiments we always found a 3-community stable partition and very frequently (~90% of realizations) found a stable 9-community partition. In all cases, the stable partitions had high alignment with the ground truth of the corresponding SBMs, especially in the 3-community and 9-community cases. This further confirms

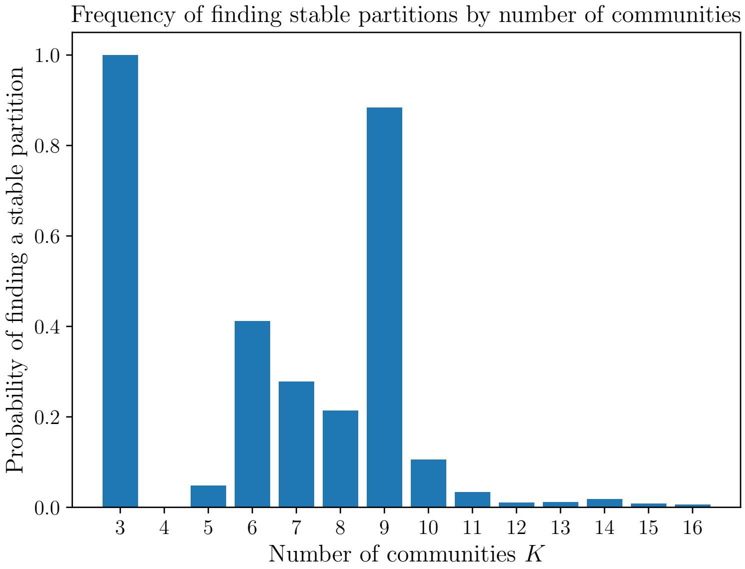

Figure S10. Observed frequency of finding stable partitions of K communities when running our pruning method on 500 realizations of the hierarchical SBM described in section E. Each run uses 1000 partitions obtained by running the Louvain heuristic on a uniform grid of $\gamma \in [0,3]$.

that our pruning procedure for finding stable partitions can work well even with modularity maximization heuristics allowing the number of communities to vary.

Interestingly, we also found stable partitions with 6 communities somewhat frequently; we visualize an example in Figure S11. Note that the 6-community stable partition here essentially corresponds to merging two of the small subcommunities in each of the larger communities in our SBM. In this case, the discovered community structure is still significant in the sense that it strongly adheres to boundaries between the smaller ground truth communities. We believe that the 6-community partitions have a higher probability of stability compared to some other choices of K due to modularity's affinity for communities of roughly equal size (compared to other $K \neq 9$ options). However, we note that it is also likely that we have randomly generated some graphs with strong 6-community splits by chance, though this effect would presumably diminish as N increases.

F More Results on the Synthetic Multilayer Temporal Network

Recall from section B.2 that a temporal network represents interactions that occurred at several different instances of time. In the multilayer network representation of such data, each layer describes a period of time and the simplest interlayer coupling is with nodes in each layer connected to the instances of the same node (that is, connecting through identity) in the previous and subsequent layers encoding the chronological sequence of layers. In this section, we focus on a synthetic test used in Pamfil et al. [2] to generate temporal networks.

The generative model, first used by Ghasemian et al. [28], is as follows. First, we generate a ground-truth community membership in the first layer by splitting the nodes evenly between K available community labels. Then, for each subsequent layer, the community label is copied from the previous layer with probability η and randomly assigned from all K possible labels with probability $1-\eta$. Using this ground-truth community assignment, edges are independently placed between pairs of nodes in each layer with probability p_{in} if the nodes are in the same ground-truth community and with probability p_{out} otherwise. A model parameter $\varepsilon = p_{\text{out}}/p_{\text{in}}$ is used to control the strength of the community structure in these layers (so smaller values of ε yield communities with denser internal connections).

F.1 Pamfil et al.'s "Easy Regime"

We first start with an "easy case" from Pamfil et al. [2] where modularity maximization correctly recovers the planted partition at the optimal parameter values. We generated multilayer networks with "copying probability" $\eta = 0.7$, edge probability ratio $\varepsilon = 0.4$, number of layers $T = 15$, number of communities $K = 2$ and 150 nodes per layer. Note that this choice of $K = 2$ means the copying of labels from one layer to the next actually occurs with probability $\eta + \frac{1}{2}(1-\eta) = 0.85$. This choice is used in both [2] and [28], so we have also chosen to use only two ground truth communities.

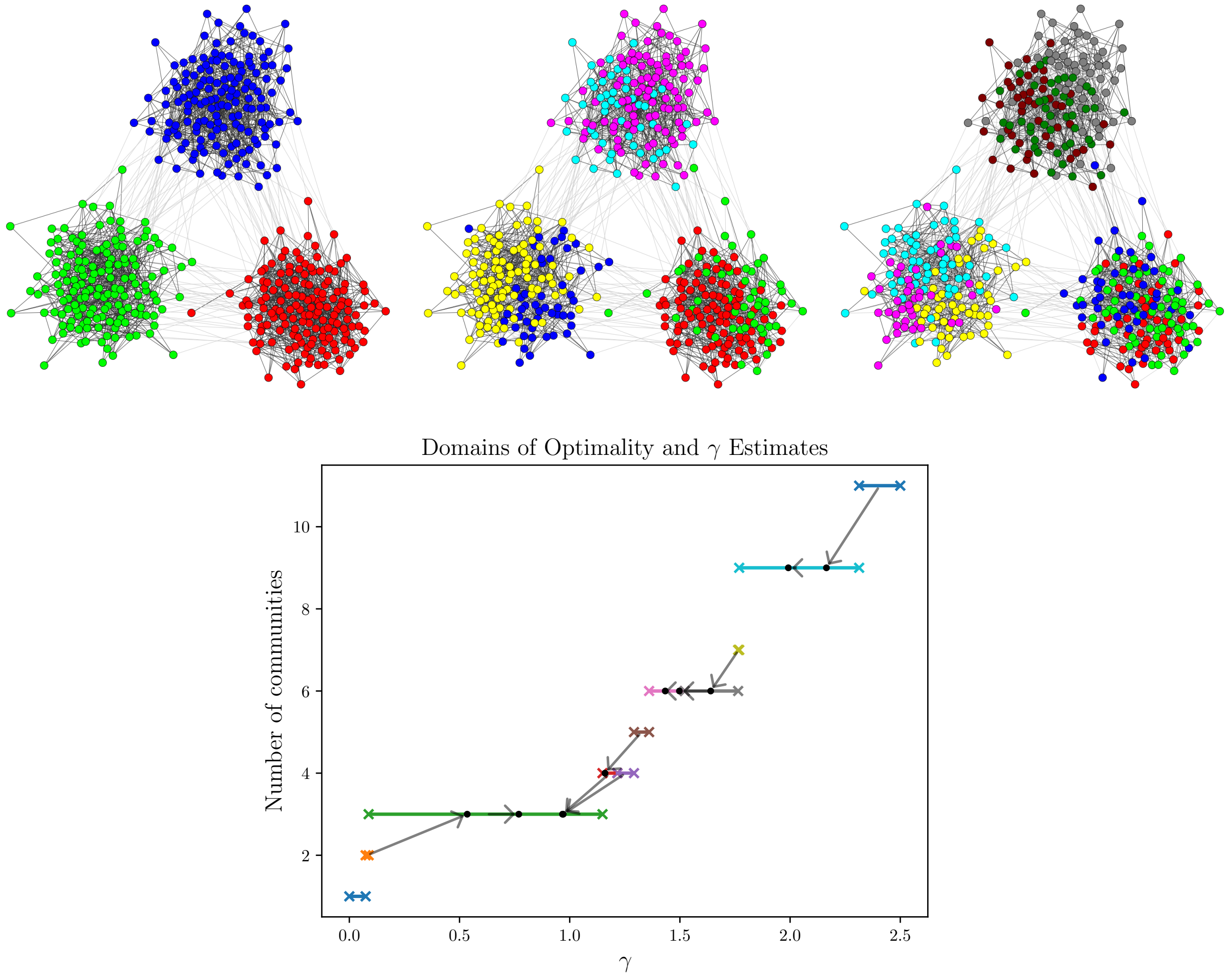

Figure S11. An example from our hierarchical SBM test in which our pruning framework found stable partitions with 3, 6, and 9 communities. Top: Force-directed layouts generated in igraph [7] of the stable partitions. Bottom: Domains of optimality and their corresponding γ estimates.

The behavior of Pamfil et al.'s iterative procedure on this network is shown in the left side of Figure S12. This scheme converges close to the ground truth resolution parameter estimates for much of the (ω, γ) plane, but notably there are regions for which the scheme diverges away from the ground truth values (e.g. most initializations with $\gamma > 1.1$ fail to converge to the ground truth).

We now compare these results to our method, which in part will explain why the above scheme can diverge in this way. After generating a network using the above model, we obtained 50,625 partitions by running the Louvain algorithm in a 225×225 uniform grid of $\gamma \in [0,2]$, $\omega \in [0,2]$. Of these partitions, 27,639 were unique with more than one community. We start pruning by inputting these unique partitions into with CHAMP *with the number of communities left unconstrained*. Across the resolution parameter area $\gamma \in [0,2]$, $\omega \in [0,2]$, our admissible subset from CHAMP has 91 partitions with more than one community. The domains of optimality and associated resolution parameter estimates are shown in the right side of Figure S12.

Qualitatively, these domains exhibit the same behavior as Pamfil et al.'s iterative procedure with many partitions' estimates lying close to the ground truth resolution parameter values of $(\omega, \gamma) \approx (0.98, 0.94)$. Once again, the partitions dominant in the $\gamma \gtrsim 1.1$ region do not converge near this ground truth. To see why this is the case, we plot the number of communities in these partitions and the values of their adjusted mutual information (AMI) with the ground truth 2-community partition in Figure S13. AMI is a measure of how closely two partitions agree where values closer to 1 indicate stronger alignment and 0 indicates no alignment; in contrast to normalized mutual information (NMI), AMI is adjusted for chance so that random clustering labels have an expected AMI of 0. Here, we see the issue is precisely the one discussed in section B.5 where the resolution parameter estimates depend heavily on the number of communities in the partition. The transition around $\gamma \approx 1.1$ is

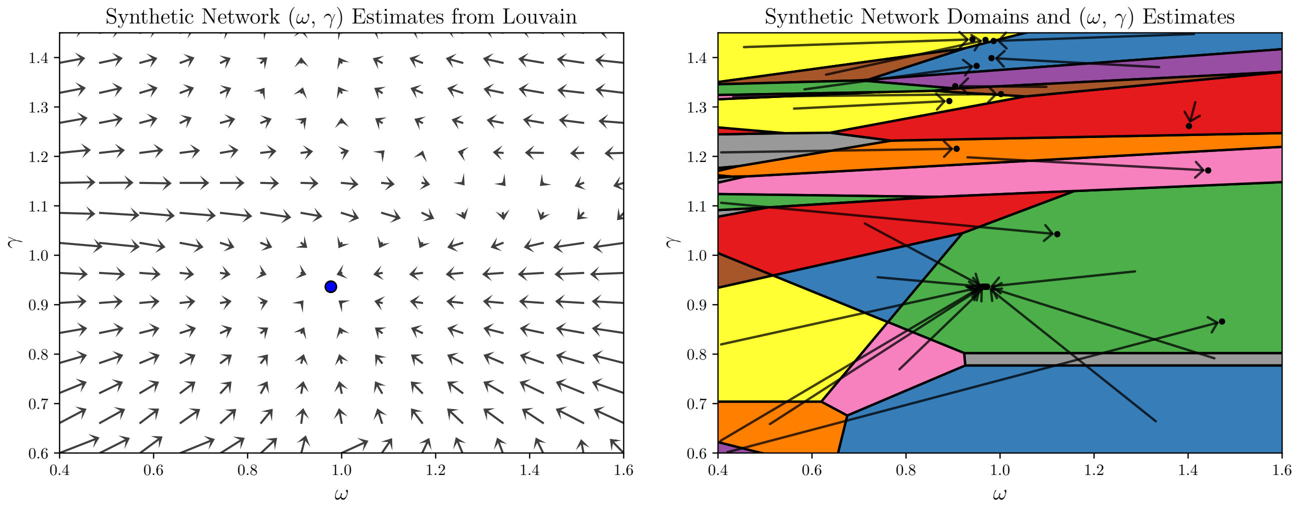

Figure S12. Left: The behavior of the iterative procedure introduced in Pamfil et al. [2] on our synthetic network. The parameter values for the ground truth community are shown as a blue point near $(\omega, \gamma) \approx (0.98, 0.94)$. Over a grid of the (ω, γ) plane, arrows indicate the direction of the updated resolution parameter estimates after maximizing modularity with the Louvain algorithm for this choice of (ω, γ), averaged over five trials. As in [2], arrow sizes are scaled down for clarity (here, shown as 10% their actual update movement). Right: Domains of optimality for the partitions in CHAMP's pruned subset (approximately 25 partitions are somewhere dominant in the region of the (ω, γ) plane shown). For each partition, an arrow is drawn from the centroid of the partition's domain of optimality to its resolution parameter estimate (ω, γ).

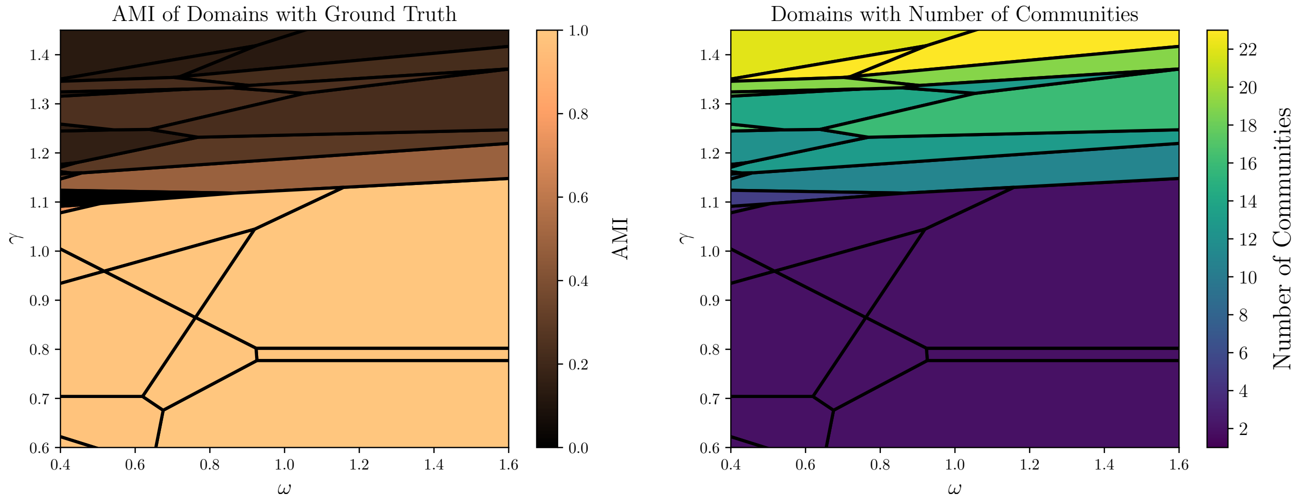

Figure S13. Left: Domains of optimality from CHAMP's pruned subset, colored by AMI with the ground truth partition. Right: Domains of optimality from CHAMP's pruned subset, colored by number of communities.

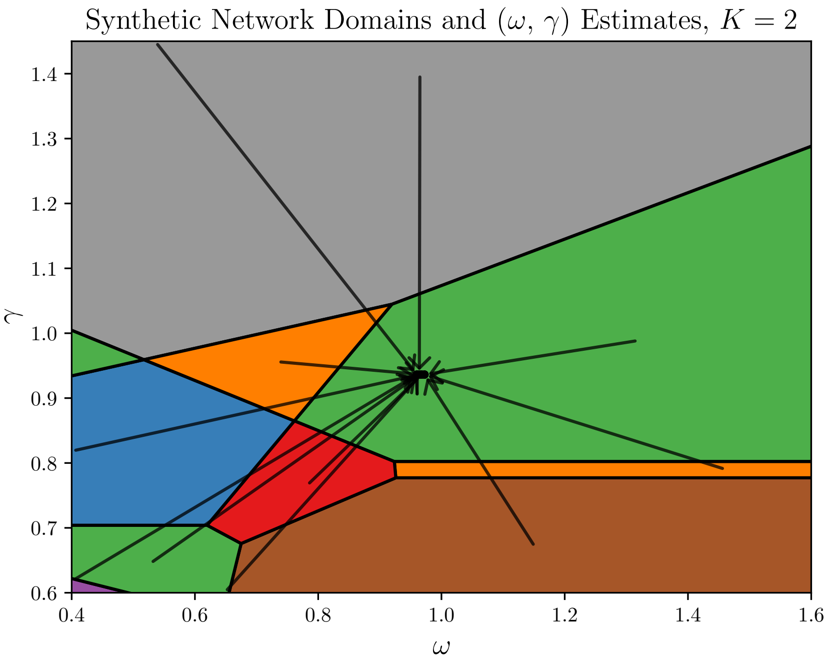

Figure S14. Domains of optimality for the partitions in CHAMP's pruned subset when we restrict $K = 2$. For each partition, an arrow is drawn from the centroid of the partition's domain of optimality to its resolution parameter estimate (ω, γ).

marked by a rapid increase in the number of communities from $K = 2$ to $K \approx 15$. Unsurprisingly, this transition also coincides with a sharp decrease in alignment with the ground truth partition.

Number of communities K	AMI with ground truth	(ω, γ) estimate
2	0.99	(0.97, 0.94)
14	0.24	(0.89, 1.31)
16	0.23	(1.40, 1.26)
22	0.13	(0.94, 1.44)
23	0.13	(0.99, 1.43)
26	0.20	(0.95, 1.51)

Table S2. Details of the stable partitions from CHAMP's pruned subset on this synthetic network. Recall that the ground truth resolution parameter estimates here are $(\omega, \gamma) \approx (0.98, 0.94)$.

Now, we analyze the stable partitions from CHAMP's pruned subset (once again, with the number of communities unconstrained): 6 out of the 91 partitions with more than one community are (stable) fixed points. The details of these partitions are given in Table S2. Notably, the 2-community stable partition has very strong alignment with the ground truth (agreeing for ~99.9% of the network's nodes). Indeed, it has the highest AMI value with the ground truth group membership among any of the partitions in CHAMP's pruned subset. The domains of all other stable partitions lie beyond the transition where the number of communities increases at $\gamma \approx 1.1$ and do not closely match the ground truth.

Finally, we reproduce Figure S12 when CHAMP's pruned subset only considers partitions with $K = 2$, constituting 2,507 unique partitions from the original runs of the Louvain algorithm. We show the domains of optimality and (ω, γ) estimates in Figure S14. In this case, CHAMP's pruned subset has 29 partitions that are somewhere dominant, exactly one of which is stable (that is, a fixed point). This is the same high-AMI, stable 2-community partition discovered when K was left unconstrained. (As we've seen before, stability of a partition persists when restricting focus to a smaller subset of partitions.) Moreover, the diverging behavior that we saw for $\gamma \gtrsim 1.1$ does not exist when fixing $K = 2$.

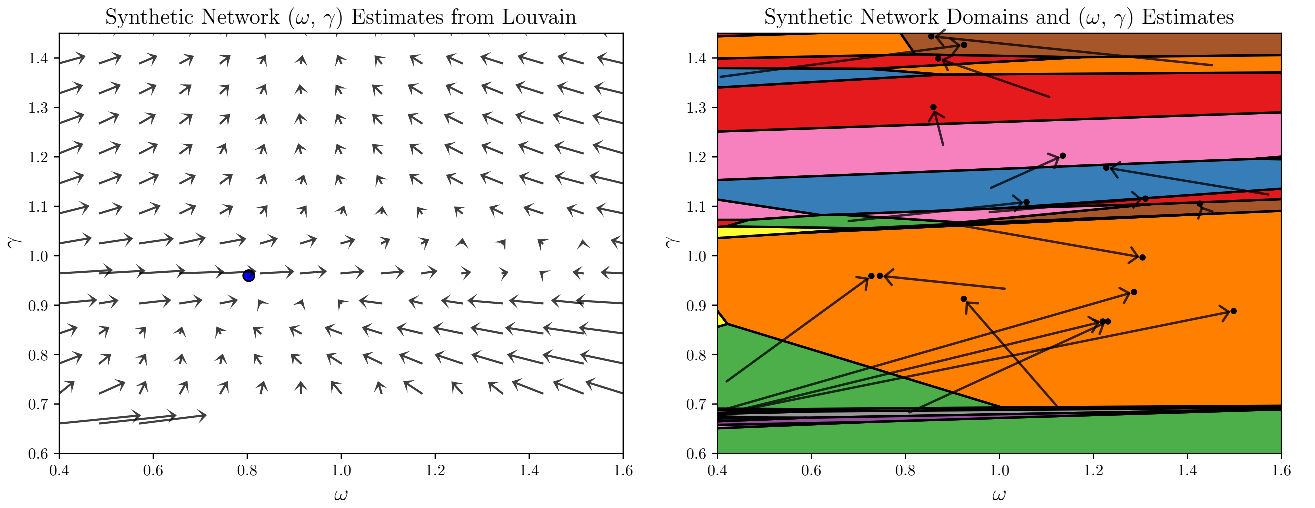

Figure S15. Left: The behavior of the iterative procedure introduced in Pamfil et al. [2] on our "hard regime" synthetic network. The parameter values for the ground truth community are shown as a blue point near $(\omega, \gamma) \approx (0.80, 0.96)$. Over a grid of the (ω, γ) plane, arrows indicate the direction of the updated resolution parameter estimates after maximizing modularity with the Louvain algorithm for this choice of (ω, γ), averaged over five trials (when this value exists, i.e. Louvain returns a partition of more than one community). As in [2], arrow sizes are scaled down for clarity (here, shown as 10% their actual update movement). Right: Domains of optimality for the partitions in CHAMP's pruned subset. For each partition, an arrow is drawn from the centroid of the partition's domain of optimality to its resolution parameter estimate (ω, γ).

F.2 Pamfil et al.'s "Hard Regime"

We also test a "hard case" from Pamfil et al.'s paper in which their iterations fail to converge to the ground truth planted partition. We show that our method continues to recover the ground truth community structure even in this case.

As before, we generated multilayer networks with $T = 15$ layers, $K = 2$ ground truth communities, and 150 nodes per layer. To match [2], we also choose $\eta = 0.5$, $\varepsilon = 0.5$. Once again, we show the behavior of Pamfil et al.'s iterative procedure on this network in the left side of Figure S15. We again ran the Louvain algorithm on a 225×225 uniform grid of $\gamma \in [0, 2]$, $\omega \in [0, 2]$ and show the resulting CHAMP domains of optimality and resolution parameter estimates in the right side of Figure S15. Here, the pruning with CHAMP keeps the number of communities unconstrained.

Importantly, the iterative scheme fails to converge near the ground truth resolution parameter estimates, regardless of the initialization values for (ω, γ) while our method successfully finds a stable $K = 2$ partition with resolution parameter estimates near the ground truth (even without fixing $K = 2$). Pamfil et al. cleverly address this problem by backtracking along trajectories, noting:

> "However, by returning the largest-modularity partition that it encounters during the iterative process, the algorithm identifies a solution that is close to the planted structure. (The NMI is about 0.86.)" [2]

But this technique is certainly not guaranteed to work in general and the direct comparison of modularity across different choices of resolution parameters and number of communities is difficult. Moreover, we find that their largest-modularity partition does not significantly improve upon the underlying heuristic (Louvain) in regions where it frequently returns partitions with two communities. Across 1000 trials each on our network realization,

- Running Louvain at $(\omega, \gamma) = (0.25, 0.70)$ yields an NMI ≥ 0.86 approximately 25% of the time (with median $\approx$0.582)
- Running Louvain at $(\omega, \gamma) = (0.50, 0.80)$ yields an NMI ≥ 0.86 approximately 33% of the time (with median $\approx$0.845)
- Running Louvain at $(\omega, \gamma) = (1.00, 0.75)$ yields an NMI ≥ 0.86 approximately 59% of the time (with median $\approx$0.866)
- Running Louvain at $(\omega, \gamma) = (1.50, 0.80)$ yields an NMI ≥ 0.86 approximately 29% of the time (with median $\approx$0.844)

On the other hand, our method yields an NMI of $\approx$0.909 between the ground truth partition and the stable $K = 2$ partition. Intuitively, our use of CHAMP prunes lower-quality results from Louvain across the entire resolution parameter plane, so our method can be more robust to the heuristic's inability to find the "correct" number of communities near the ground truth parameter estimates. In fact, you can see in Figure S15 that the domain of optimality for the stable $K = 2$ partition extends into the region where Pamfil et al.'s iterative procedure diverges away from the ground truth (ω, γ). This is primarily due to Louvain

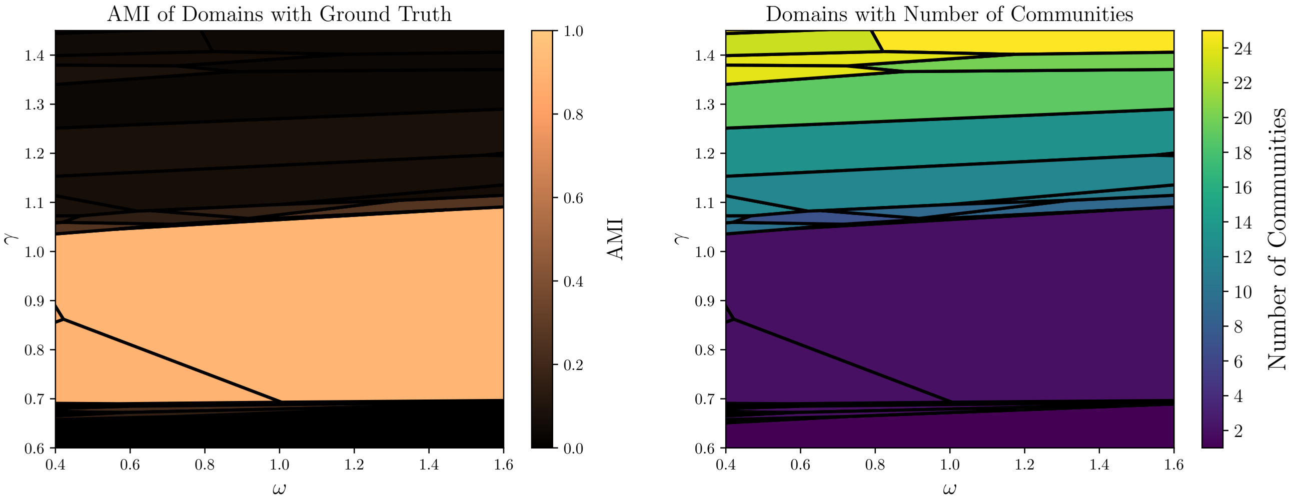

Figure S16. Left: Domains of optimality from CHAMP's pruned subset, colored by AMI with the ground truth partition. Right: Domains of optimality from CHAMP's pruned subset, colored by number of communities.

returning partitions of $K > 2$ in regions where this is not optimal from the perspective of modularity. In contrast, our method's use of CHAMP prunes away such $K > 2$ partitions for (ω, γ) choices in this region where a $K = 2$ partition is indeed optimal.

This is also evident when we plot the CHAMP set, colored by number of communities and their AMIs with the ground 2-community partition. This is shown in Figure S16. We can see that the region of divergence for Pamfil et al.'s scheme matches the rapid increase of the optimal number of communities from the perspective of modularity (and thus, a sharp decrease in alignment with the ground truth partition). Apparently, Louvain fails to find this transition accurately and starts returning partitions of $K > 2$ "too early" as γ increases, so our post-processing with CHAMP helps find the stable partition.

Moreover, we again note that this issue disappears when we restrict attention to $K = 2$ in our method. We plot the domains of optimality and (ω, γ) estimates in Figure S17 and find that any potential diverging behavior ceases to exist.

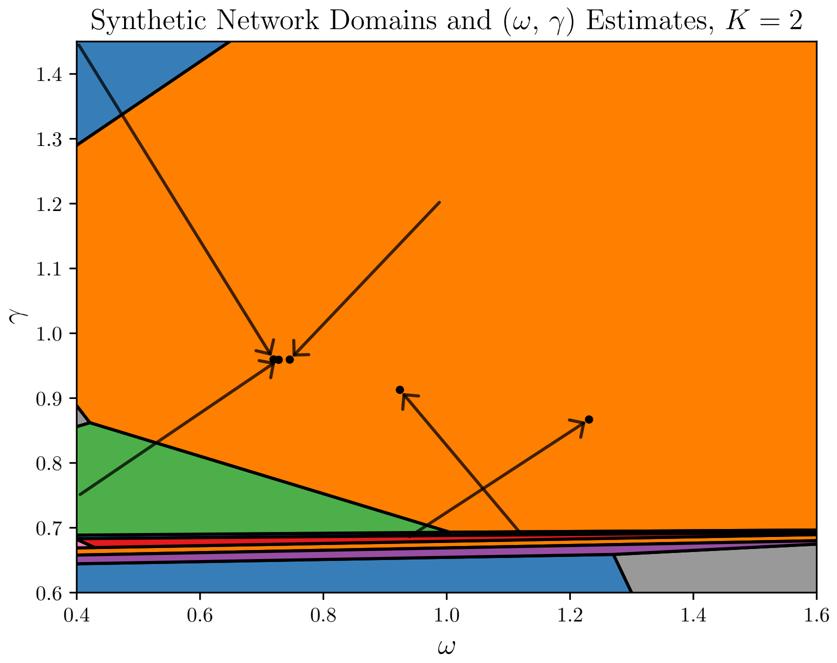

Figure S17. Domains of optimality for the partitions in CHAMP's admissible subset when we restrict $K = 2$. For each partition, an arrow is drawn from the centroid of the partition's domain of optimality to its resolution parameter estimate (ω, γ).

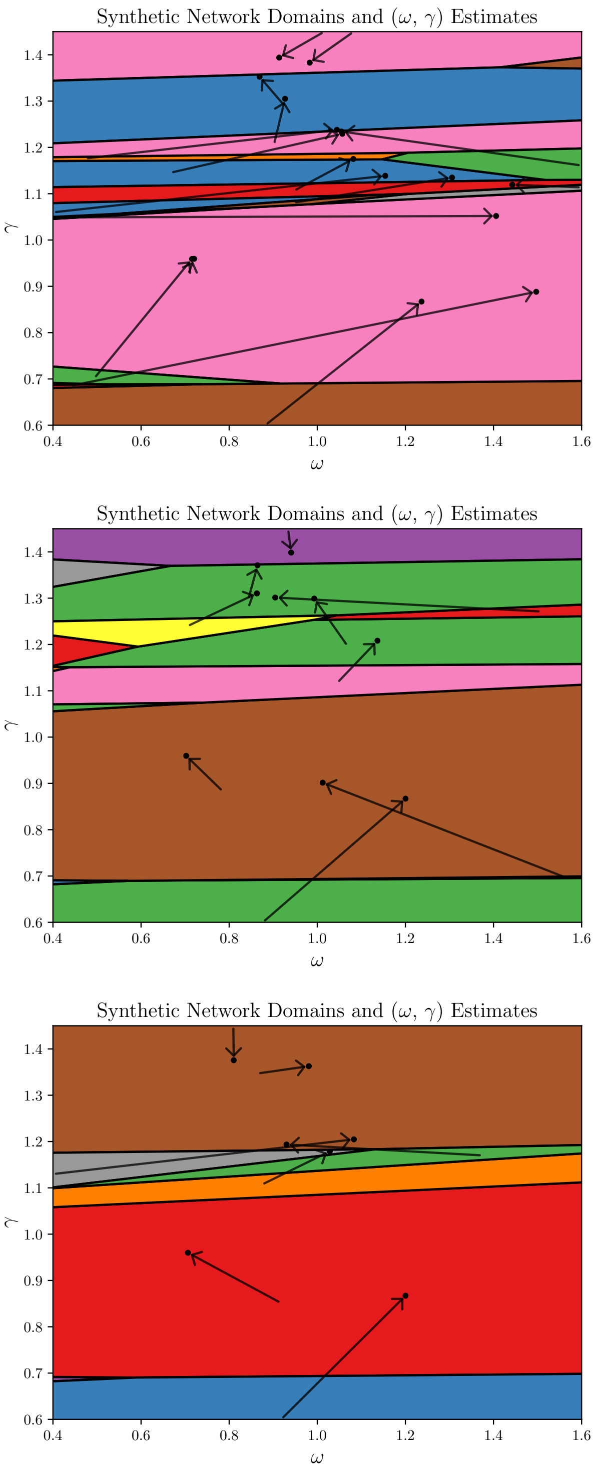

Figure S18. Domains of optimality for the partitions in CHAMP's admissible subset when running on a "hard regime" synthetic network with a small number of input partitions. For each partition, an arrow is drawn from the centroid of the partition's domain of optimality to its resolution parameter estimate (ω, γ). Top: Results from method 1 with 1024 input partitions. Middle: Results from method 2 with 100 input partitions. Bottom: Results from method 3 with 25 input partitions.

F.3 Pamfil et al.'s "Hard Regime" with a Small Number of Input Partitions

Above, we used a 255×255 uniform grid of $\gamma \in [0,2]$, $\omega \in [0,2]$ to run Louvain, providing our method with 50,625 input partitions. We have noted before (e.g. in section D) that we are intentionally being exhaustive here and the results are qualitatively similar with significantly fewer partitions. Now, we show that this is indeed the case on this example as well. Specifically, we consider the CHAMP domains of optimality and resolution parameter estimates from our method on the "hard regime" network from the previous subsection when using a small number of input partitions. Here, we use the range of resolution parameters from [2] ($0.7 \le \gamma \le 1.2$ and $0.5 \le \omega \le 1.5$) and the following methods for obtaining input partitions:

1. Using 1024 input partitions from a 32×32 uniform grid of Louvain runs.
2. Using 100 input partitions from a 10×10 uniform grid of Louvain runs.
3. Using 25 input partitions from a 5×5 uniform grid of Louvain runs.

We show the CHAMP domains and resolution parameter estimates from these experiments in Figure S18.

In all cases, the domain containing the ground truth parameter estimates $(\omega, \gamma) \approx (0.80, 0.96)$ is a stable partition with $K = 2$. As such, even with a small number of input partitions, our method continues to recover a stable partition with high alignment to the ground truth planted partition.

Notably, Pamfil et al.'s iterative scheme (as implemented in [29]) runs Louvain 20 times before claiming a failure to converge, so our third method with 25 input partitions takes approximately the same amount of computing power as a single attempt of their iterative algorithm. Moreover, we can obtain our input partitions in parallel (i.e. on multiple processors simultaneously). As such, we believe that our method can be very performant in practice.

G More Results on the Lazega Law Network

Following Pamfil et al. [2], we now demonstrate our approach on the Lazega Law Firm network [6]. This is a 3-layer multiplex network that describes the relationships between 71 attorneys. In particular, the individuals were asked to list

1. The members of the firm that they go to for basic professional advice.
2. The members of the firm that they closely work with.
3. The members of the firm that they socialize with outside of work.

These directed associations were then used to form three layers of a network, referred to as the "Advice", "Coworker", and "Friend" layers, respectively. Each node (representing an individual) is connected by the notion of identity to its copy in all other layers to form the complete multiplex network with 213 node-layers (71 in each of the 3 layers). As a multilayer network, of course, the interlayer identity connections are handled differently from the intralayer edges of the three types of relationships. This network is also annotated with various pieces of metadata, which we will use to analyze our results. Here, in order to compare with [2], we will use the following pieces of metadata: status ("partner" or "associate"), gender, office (3 possibilities), seniority (years with the firm, grouped into 5-year bins), age (grouped into 5-year bins), practice ("litigation" or "corporate"), and law school (4 options, one of which is "other").

We ran 1,000,000 instances of the Louvain algorithm on a uniform 2000×500 grid of $\gamma \in [0,2]$, $\omega \in [0,3]$, which identified 211,219 unique partitions with more than one community. (We again note that this large number of iterations is excessive, but we choose to err on the side of being exhaustive here. We will return to assessing the performance of our scheme for fewer runs on this network at the end of this section.) The admissible subset from CHAMP (where we do not yet restrict the number of communities K) has 152 unique partitions with more than one community. Details of these partitions are given in Table S3 and their domains of optimality are shown in Figure S19 and Figure S20.

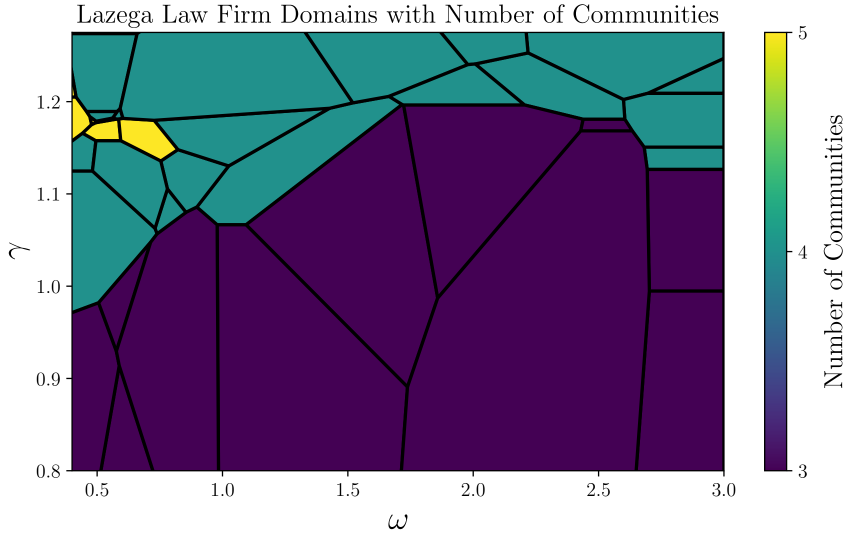

Figure S19. Domains of optimality for the partitions in CHAMP's admissible subset, colored by number of communities.

Number of communities K	2	3	4	5	6	7	≥ 8
Number of unique partitions	2.6K	30.4K	34.1K	22.4K	19.5K	18.2K	83.9K
Number of unique partitions in CHAMP's pruned subset	8	19	23	22	15	13	52
Number of unique partitions in CHAMP's pruned subset when only considering partitions with K communities	38	45	52	71	55	63	-
Number of stable partitions in CHAMP's pruned subset when only considering partitions with K communities	3	2	3	3	2	2	-

Table S3. A breakdown of the unique partitions returned by the running the Louvain algorithm 1 million times on the Lazega Law Firm network and the resulting pruned subsets from CHAMP.

As visualized in Figure S20, there are three fixed points of the map on the CHAMP set when allowing K to vary (one with $K = 3$ and two with $K = 4$). We additionally consider restricting $K = 2$, 3, and 4 prior to post-processing with CHAMP, which reveals five more stable partitions for fixed K. These domains are shown in Figure S21 and the associated community memberships are visualized in Figure S22. Comparing with Figure 4 of [2], their two highlighted groups of partitions that they consensus cluster appear to best correspond to the $K = 3$ fixed point with $\omega \approx 0.7$ that we find when restricting to fixed-K maps and the stable $K = 3$ domain at $\omega = \infty$.

As one might expect, the community labels per individual match closely between the layers that represent their close coworkers and the members of the firm that they go to for advice. However, we often find individuals that are placed in a community in the "Friend" layer that differs from their community in the "Advice" or "Coworker" layers.

We now compute the alignment of our stable partitions with the metadata and compare our results to those of Pamfil et al. [2]. In short, Pamfil et al. ran their iterative procedure 100 times, identified two primary clusters of common convergence points (ω, γ), and used consensus clustering to convert these clusters into three partitions, each with 3 communities. As in [2], we will refer to these as "cluster 1" and "cluster 2". These results are shown in Table S4.

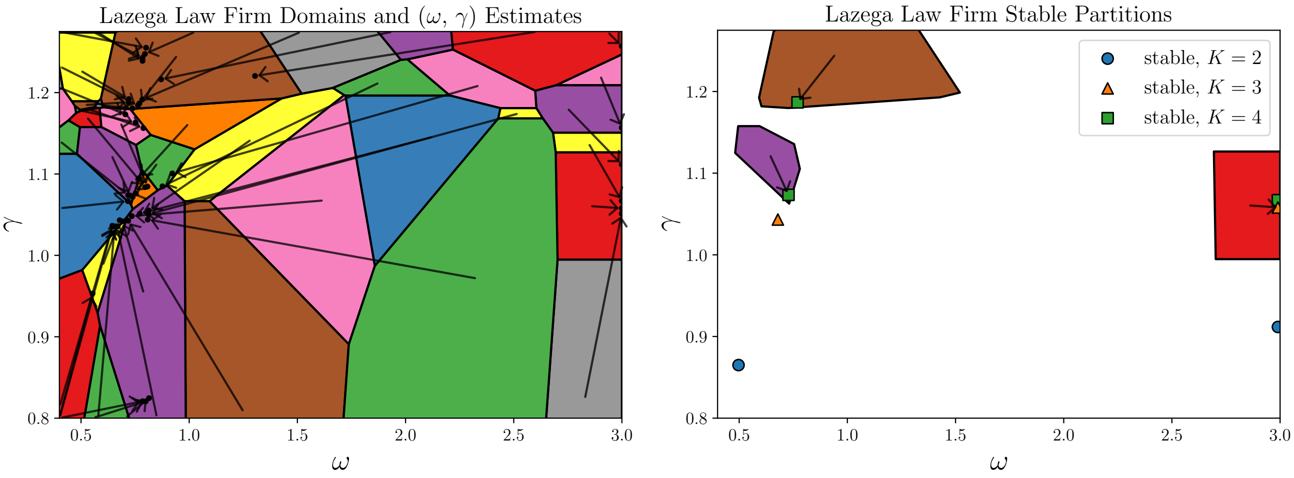

Figure S20. Domains of optimality for the partitions in CHAMP's pruned subset with K left unconstrained. Left: Domains are annotated with arrows that indicate their partition's resolution parameter estimates (ω, γ). Partitions with communities that are the same across all layers have an estimate of $\omega = \infty$, so we have truncated to $\omega = 3$ for plotting purposes. Right: Domains and resolution parameter estimates for the three stable partitions. Additional points indicate the resolution parameter estimates for the stable partitions found by separately fixing $K = 2,3,4$ prior to running CHAMP (stable domains for each choice of K are shown in Figure S21).

	Approximate (ω, γ)	Office	Practice	Age	Seniority	Status	Gender	Law School
Stable Partition 1, $K = 2$	$(0.5, 0.9)$	0.192	0.163	0.033	0.024	0.002	0.000	0.006
Stable Partition 2, $K = 2$	$(\infty, 0.8)$	0.854	0.000	0.072	0.031	0.037	0.016	0.007
Stable Partition 3, $K = 2$	$(\infty, 0.9)$	0.268	0.507	0.041	0.027	0.025	0.001	0.000
Stable Partition 4, $K = 3$	$(\infty, 1.1)$	0.610	0.469	0.105	0.052	0.040	0.026	0.007
Stable Partition 5, $K = 3$	$(0.7, 1.0)$	0.599	0.193	0.114	0.071	0.079	0.027	0.017
Stable Partition 6, $K = 4^{\dagger}$	$(\infty, 1.1)$	0.595	0.455	0.118	0.061	0.049	0.031	0.022
Stable Partition 7, $K = 4$	$(0.8, 1.2)$	0.525	0.212	0.159	0.117	0.150	0.091	0.026
Stable Partition 8, $K = 4^{\dagger}$	$(0.7, 1.1)$	0.563	0.196	0.116	0.090	0.077	0.035	0.020
Cluster 1, $K = 3$	$(0.4, 1.0)$	0.587	0.334	0.146	0.147	0.150	0.035	0.024
Cluster 2, $K = 3$	$(\infty, 1.0)$	0.610	0.469	0.098	0.052	0.040	0.026	0.007
Average over 100 runs	-	0.577	0.406	0.125	0.106	0.093	0.037	0.022

†Here, the fourth communities are extremely small (only one node in the case of Stable Partition 6), so one could also group these with $K = 3$.

Table S4. Normalized Mutual Information (NMI) scores of the eight stable partitions under fixed-K iterative maps with $K = 2,3,4$ and the two consensus clusterings from Pamfil et al. [2].

Our stable partitions 5 and 8 roughly match cluster 1 from [2] and our stable partitions 4 and 6 roughly match cluster 2 from [2]. Among our other identified partitions, we note that stable partition 2 has the strongest alignment with the office metadata and stable partition 3 has the strongest alignment with the practice metadata.

One might reasonably question whether we recover more information about the network simply because we have run modularity maximization heuristics an extremely large number of times here. However, when we use only 25 runs of Louvain on a 5×5 uniform grid of the more reasonable range $\gamma \in [0.5, 1.5]$, $\omega \in [0, 3]$, we almost always find the same convergence points (ω, γ) from Table S4. While Pamfil et al. uses 100 runs of their iterative procedure to analyze this network, each of which requires optimizing modularity multiple times, we have found that we are able to perform $\sim$2000 runs of the Louvain algorithm in the same amount of time, especially since the computations required for finding (ω, γ) estimates on multiplex networks are particularly complicated (see section B.4). We in no way suggest that the Pamfil et al.'s strategy *needs* to be run 100 times, nor that the implementation is particularly optimized, but believe this indicates our procedure can be performant in practice.

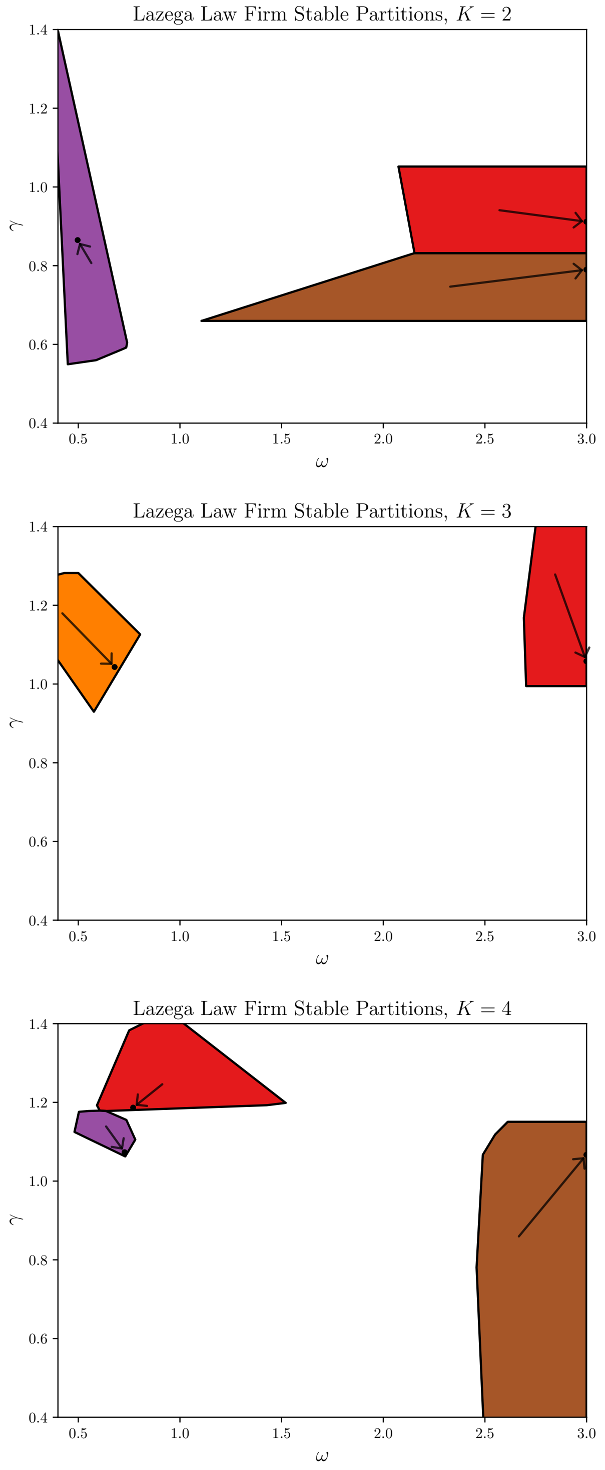

Figure S21. Domains of optimality and resolutions parameter estimates for the stable partitions when we separately fix $K = 2, 3, 4$ (shown top-to-bottom) prior to pruning with CHAMP.

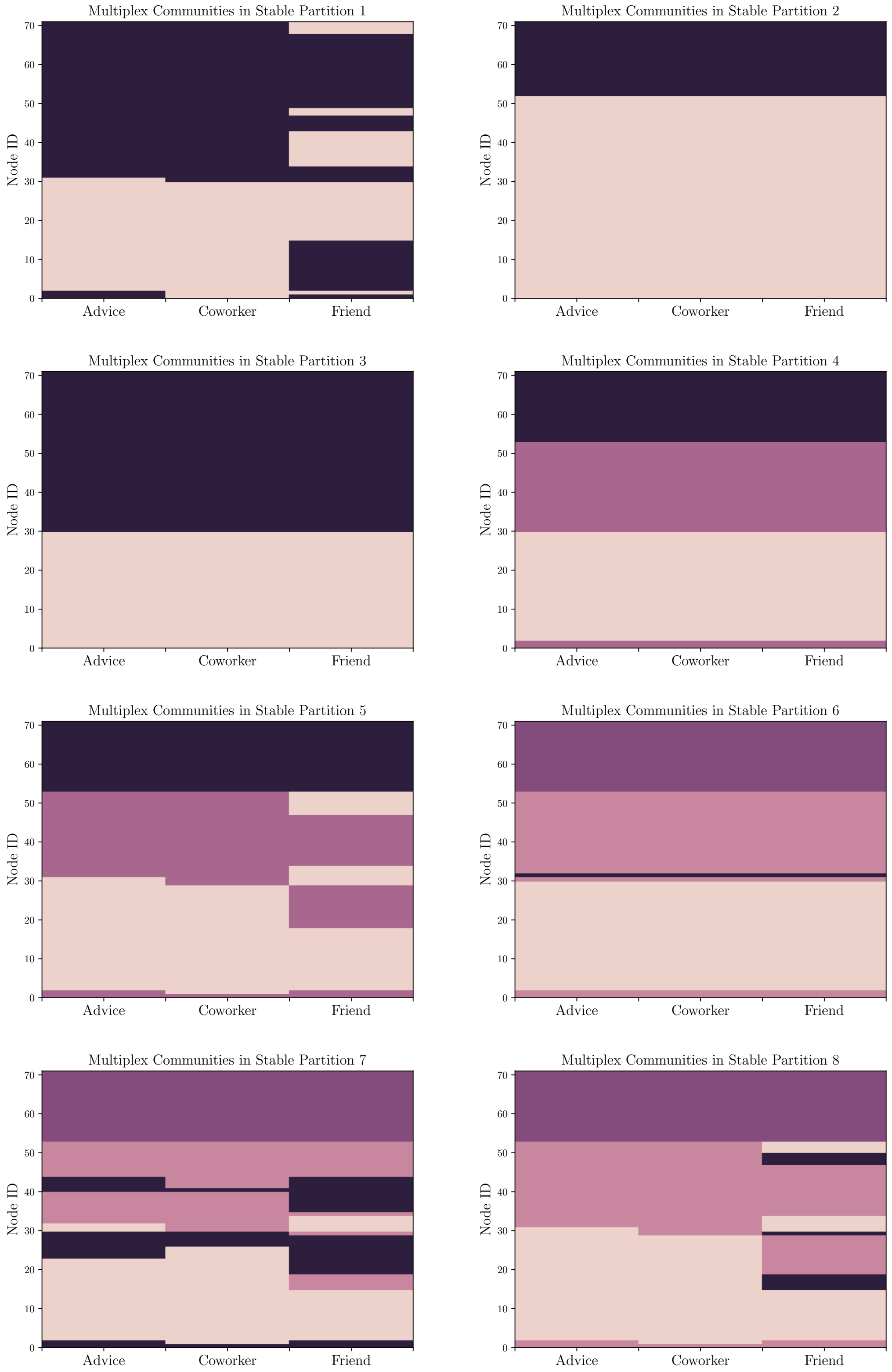

Figure S22. Visualizations of the stable partitions in the Lazega Law Firm network with $K = 2, 3, 4$. In each plot, the nodes are colored based on their community label and all plots show the same ordering of nodes. Note that stable partition 5 is very similar to stable partition 8. Also, stable partitions 4 and 6 are virtually identical.

H Explicit Construction of a "Bistable" SBM

In general, it is possible for a network to have meaningful community structure at many different scales (for example, by considering a hierarchy of communities that are each made up of smaller communities). As such, it is natural to ask whether or not a network may have multiple meaningful community structures that are simultaneously stable under our resolution parameter estimation when we allow the number of blocks K to vary. Here, we explicitly construct such a network model in which two ground truth partitions, one with 2 communities and one with 3 communities, are simultaneously stable. Hence, we refer to such a model as being "bistable".

Consider an equal-block-size SBM (without degree correction) with 3 communities in which pairs of nodes are connected between communities according to the matrix of probabilities (chosen somewhat arbitrarily)

$$\mathbf{P} = \begin{bmatrix} P_{11} & P_{12} & P_{13} \\ & P_{22} & P_{23} \\ & & P_{33} \end{bmatrix} = \begin{bmatrix} 10/99 & 1/160 & 1/160 \\ & 5/66 & \delta \\ & & 5/66 \end{bmatrix} \approx \begin{bmatrix} 0.1010 & 0.0063 & 0.0063 \\ & 0.0758 & \delta \\ & & 0.0758 \end{bmatrix}, \tag{S18}$$

where δ is a tunable parameter. When δ is small, the SBM exhibits strong 3-block structure, but as δ increases towards $5/66$ the SBM exhibits strong 2-block structure. For some intermediate values of δ, however, we could say that a 3-community ground truth partition coincides with the "2-community ground truth" that merges blocks 2 and 3. We make the simplifying assumption that self loops do not exist.

Using an approach similar to what we will do below in section J, we can identify the expected values associated with the resolution parameter estimates in the modularity-SBM equivalence, obtaining

$$\begin{aligned}
m_{\text{in}}^{\text{3 community ground truth}} &= \binom{B}{2} \cdot \sum_i P_{ii} \\
m_{\text{out}}^{\text{3 community ground truth}} &= B^2 \cdot \sum_{i<j} P_{ij} \\
m_{\text{in}}^{\text{2 community ground truth}} &= \binom{B}{2} \cdot \sum_i P_{ii} + B^2 \cdot P_{23} \\
m_{\text{out}}^{\text{2 community ground truth}} &= B^2 \cdot \sum_{i<j} P_{ij} - B^2 \cdot P_{23} \\
\kappa_1^{\text{3 community ground truth}} &= B(B-1)P_{11} + B^2[P_{12} + P_{13}] \\
\kappa_2^{\text{3 community ground truth}} &= B(B-1)P_{22} + B^2[P_{12} + P_{23}] \\
\kappa_3^{\text{3 community ground truth}} &= B(B-1)P_{33} + B^2[P_{13} + P_{23}] \\
\kappa_1^{\text{2 community ground truth}} &= B(B-1)P_{11} + B^2[P_{12} + P_{13}] \\
\kappa_2^{\text{2 community ground truth}} &= B(B-1)[P_{22} + P_{33}] + B^2[P_{12} + P_{13} + 2P_{23}],
\end{aligned} \tag{S19}$$

where $\kappa_r = \sum_i k_i \cdot \delta(g_i, r)$ is the sum of degrees in group r. Now, we rewrite modularity as

$$Q(\gamma) = \frac{1}{2m} \sum_{i,j} \left(A_{ij} - \gamma \frac{k_j k_j}{2m} \right) \delta_{g_i g_j} = \frac{m_{\text{in}}}{m} - \gamma \frac{\sum_r \kappa_r^2}{4m^2}, \tag{S20}$$

which allows us to, in principle, calculate the domains of optimality for the 2-community and 3-community ground truth partitions by calculating the γ value for which their modularities become equal.

We now return to the preference matrix in Equation S18 where we explicitly fixed all values except for $P_{23} = \delta$. Then, with

N nodes and block size $B = N/3$, plugging into Equation S19 and Equation S20 yields

$$\begin{aligned}
m_{\text{in}}^{3 \text{ community ground truth}} &= \binom{B}{2} \cdot \frac{25}{99} \\
m_{\text{out}}^{3 \text{ community ground truth}} &= B^2 \cdot \left(\frac{2}{160} + \delta\right) \\
m_{\text{in}}^{2 \text{ community ground truth}} &= \binom{B}{2} \cdot \frac{25}{99} + B^2 \cdot \delta \\
m_{\text{out}}^{2 \text{ community ground truth}} &= B^2 \cdot \frac{2}{160} \\
\kappa_1^{3 \text{ community ground truth}} = \kappa_1^{2 \text{ community ground truth}} &= B(B-1) \cdot \frac{10}{99} + B^2 \cdot \frac{2}{160} \\
\kappa_2^{3 \text{ community ground truth}} = \kappa_3^{3 \text{ community ground truth}} &= B(B-1) \cdot \frac{5}{66} + B^2 \cdot \left(\frac{1}{160} + \delta\right) \\
\kappa_2^{2 \text{ community ground truth}} &= B(B-1) \cdot \frac{10}{66} + B^2 \cdot \left(\frac{2}{160} + 2\delta\right),
\end{aligned} \tag{S21}$$

and

$$\begin{aligned}
Q^{3 \text{ community ground truth}}(\gamma) &= \frac{1000(B-1)}{B(7920\delta + 1099) - 1000} - \gamma \cdot \frac{2\left(B^2\left(\delta + \frac{433}{5280}\right) - \frac{5B}{66}\right)^2 + \left(\frac{B^2}{80} + \frac{10}{99}(B-1)B\right)^2}{4\left(B^2\left(\delta + \frac{1}{80}\right) + \frac{25}{198}(B-1)B\right)^2} \\
Q^{2 \text{ community ground truth}}(\gamma) &= \frac{99B}{-7920B\delta - 1099B + 1000} + 1 - \gamma \cdot \frac{\left(B^2\left(2\delta + \frac{1}{80}\right) + \frac{5}{33}(B-1)B\right)^2 + \left(\frac{B^2}{80} + \frac{10}{99}(B-1)B\right)^2}{4\left(B^2\left(\delta + \frac{1099}{7920}\right) - \frac{25B}{198}\right)^2}.
\end{aligned} \tag{S22}$$

By equating these two modularity functions, we find that the γ value for which the 3-community ground truth partition becomes dominant over the 2-community partition is given by

$$\begin{aligned}
\gamma_{\text{cross}} &= \frac{7040B\delta(B(7920\delta + 1099) - 1000)}{(B(5280\delta + 433) - 400)^2} \\
\lim_{N \to \infty} \gamma_{\text{cross}} &= \frac{7040\delta(1099 + 7920\delta)}{(433 + 5280\delta)^2}.
\end{aligned} \tag{S23}$$

Finally, we can compute expected ground truth estimates ω_{in}, ω_{out} and γ,

$$\begin{aligned}
\omega_{\text{in}}^{3 \text{ community ground truth}} &= \frac{80(80\delta + 11)}{320\delta(80\delta + 13) + 331} \\
\omega_{\text{out}}^{3 \text{ community ground truth}} &= \frac{8(80\delta + 1)(80\delta + 11)}{(160\delta + 13)(160\delta + 49)} \\
\omega_{\text{in}}^{2 \text{ community ground truth}} &= \frac{256\delta + 19}{32\delta(80\delta + 13) + 25} + 1 \\
\omega_{\text{out}}^{2 \text{ community ground truth}} &= \frac{80(80\delta + 11)}{320\delta(80\delta + 13) + 331} \\
\gamma^{3 \text{ community ground truth}} &= \frac{8(80\delta + 11)^2(320\delta(80\delta - 7) - 549)}{(160\delta + 13)(160\delta + 49)(320\delta(80\delta + 13) + 331)\left(\log\left(\frac{8(80\delta+1)(80\delta+11)}{(160\delta+13)(160\delta+49)}\right) - \log\left(\frac{80(80\delta+11)}{320\delta(80\delta+13)+331}\right)\right)} \\
\gamma^{2 \text{ community ground truth}} &= \frac{2(80\delta + 11)^2(256\delta + 19)}{9(160\delta + 13)\left(2560\delta^2 + 416\delta + 25\right)\left(\log\left(\frac{4(8\delta+1)(80\delta+11)}{2560\delta^2+416\delta+25}\right) - \log\left(\frac{160\delta+22}{1440\delta+117}\right)\right)}.
\end{aligned} \tag{S24}$$

Note that all the quantities in Equation S24 do not depend on the number of nodes N or block size B since they're ultimately descriptions of the underlying SBM (whose expected values should not depend on the network size).

These results are plotted in Figure S23, demonstrating that there is a large range of choices for δ that result in the stability of both the 2-community and 3-community ground truths simultaneously. We also drew networks from this SBM in simulation to demonstrate that our analytic results appear in practice. This is shown in Figure S24. We note that in this case, the expected mean degree per node increases with N, but this is not the cause of the bistability. Indeed, a similar result can be found when

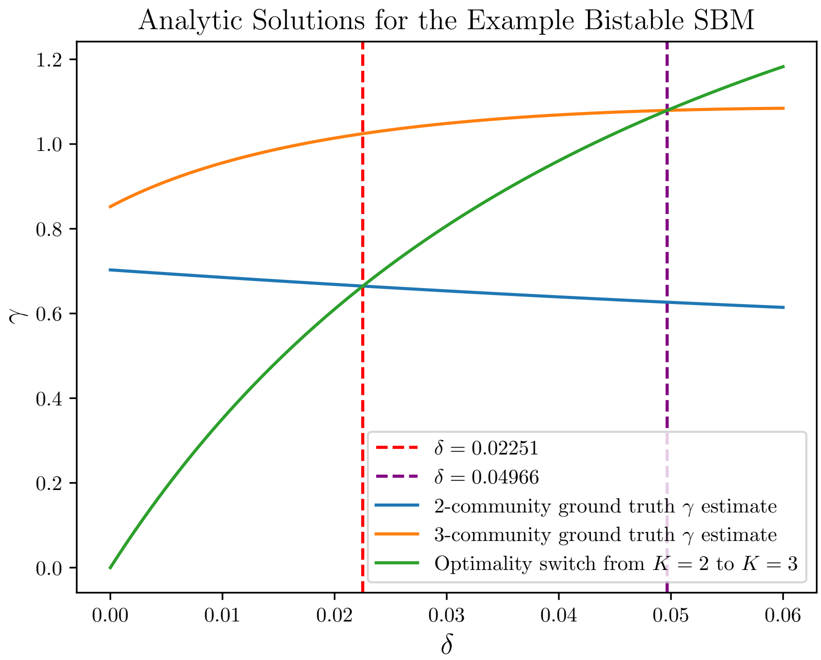

Figure S23. Ground truth γ estimates for the 2-community and 3-community ground truth partitions in our bistable SBM as $N \to \infty$. The range of choices for δ which give expected bistability is indicated between the dashed vertical lines.

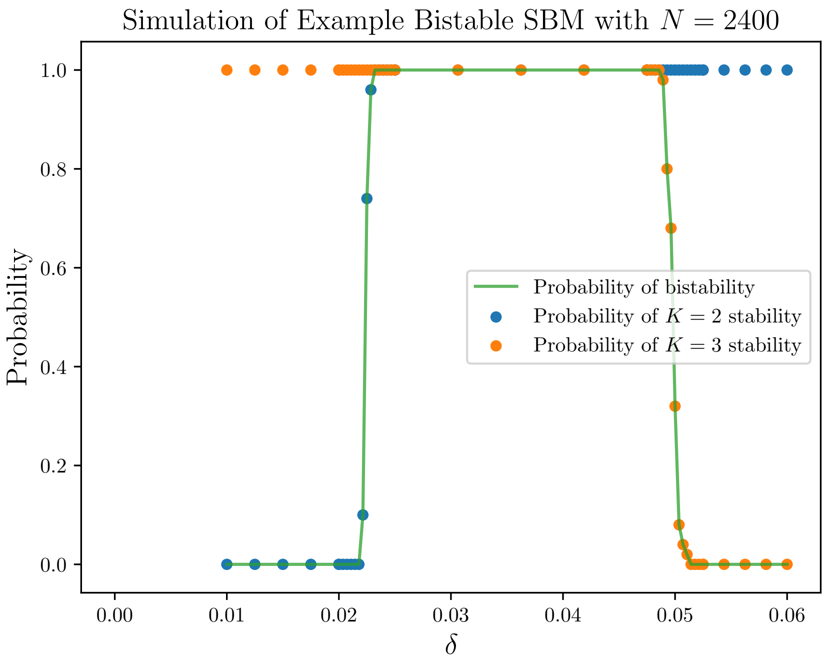

Figure S24. Empirical probability of stability for the 2-community and 3-community partitions returned by the Louvain algorithm on realizations of our example bistable SBM with $N = 2400$ (taken over 100 trials for each choice of δ).

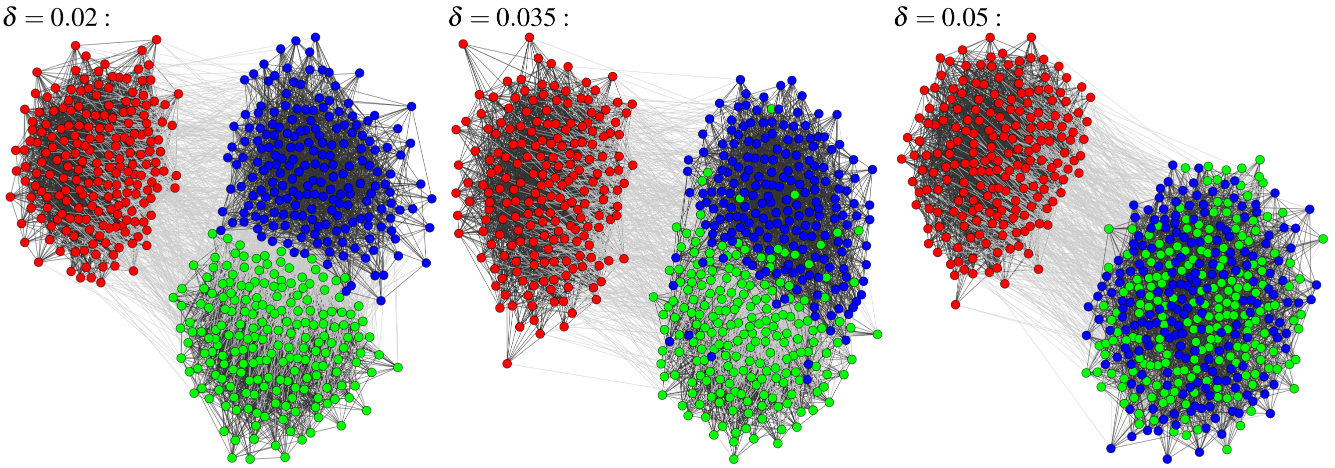

Figure S25. Force-directed layouts from igraph [7] of realizations from our bistable SBM with $N = 600$ for different values of the parameter δ (see Equation S18). Node colors indicate the 3-community ground truth (red, green, blue). Note that $\delta = 0.02$ is just below the bistable region for δ indicated in Figure S23. For comparison, $\delta = 0.035$ is in the middle of the predicted bistable region, and $\delta = 0.05$ is just above the predicted bistable region.

we scale the probabilities P_{ij} down as N increases so that the expected mean degree is fixed, provided that the P_{ij} are chosen so that the ground truth partitions are detectable.

Finally, we plot layouts of some realizations from this example bistable SBM in Figure S25. Here, the existence of "meaningful" 2-community and 3-community partitions (or lack thereof) can be visually verified as δ varies. We suspect that one could derive a completely general expression here for an arbitrary number of blocks K and preference matrix P, but considering the relative complexity of Equation S22, Equation S23, and Equation S24, such a result might not be particularly illuminating. The main purpose of the present example was to demonstrate that bistable situations like this do exist.

I Results on Lancichinetti–Fortunato–Radicchi Benchmark Networks

In this section, we evaluate the results of our pruning strategy when the assumptions of the equivalence described in section A.3 do not hold. Specifically, the two assumptions from the equivalence that we focus on are

1. The number of communities is fixed during modularity maximization, and
2. The network of interest has a latent structure that contains communities of roughly equal size.

We note again that for arbitrary choice of resolution parameter γ, modularity maximization is *always* equivalent to the stochastic block model inference from this equivalence and thus may be expected to suffer from violation of these same assumptions. To explore the effects of violating these assumptions, we here consider the performance of the pruning strategy on the LFR benchmark networks of Lancichinetti et al. [30] which generate graphs with communities of nonuniform size, using the implementation of the benchmark in the NetworkX graph library [31].

I.1 Review of the LFR Algorithm

The LFR benchmark algorithm [30] generates networks in which the distribution of community sizes and node degrees both follow power laws. This is motivated by the observation that real networks have heavy-tailed degree distributions, often containing a small number of "hubs" (nodes with very high degree relative to the rest of the graph). Similarly, the sizes of communities might vary wildly in real networks. The LFR generation is parameterized by five values (we use the notation of NetworkX [31] here to avoid using γ in two different contexts):

- N, the desired number of nodes in the network
- τ_1, the exponent of the power law distribution used to generate node degrees
- τ_2, the exponent of the power law distribution used to generate community sizes
- $\langle k \rangle$, the average node degree
- μ, a mixing parameter. The network will be generated such that each node is expected to share a fraction $1-\mu$ of its links with nodes in its own community and μ with nodes of other communities.

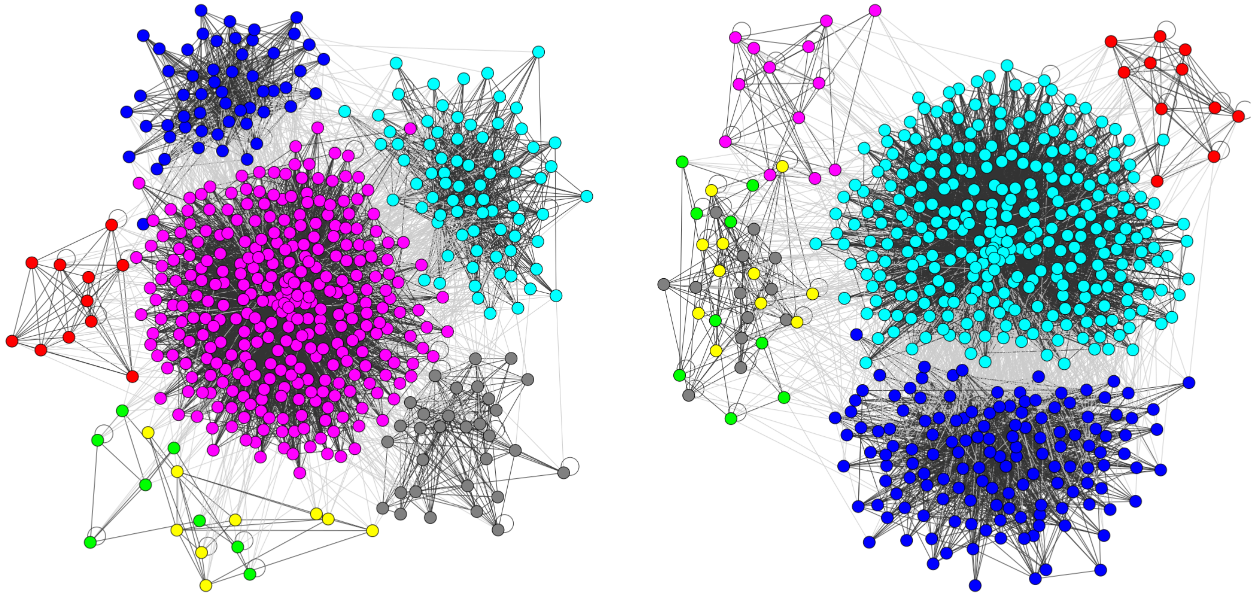

Figure S26. Force-directed layouts generated in igraph [7] of two realizations of the LFR algorithm with $N = 500$, $\tau_1 = 2.5$, $\tau_2 = 1.5$, $\mu = 0.1$ and $\langle k \rangle = 20$. These parameter choices closely match those used by Lancichinetti et al. and are believed to generate realistic networks with a heterogenous distribution of node degrees and community sizes.

We note that Lancichinetti et al. [30] claim that real networks typically have $2 \leq \tau_1 \leq 3$ and $1 \leq \tau_2 \leq 2$; as such, they recommend these ranges to generate realistic graphs. The algorithm proceeds in four main steps:

1. Each node is assigned a degree drawn from the power law distribution with exponent τ_1. This distribution is truncated to the range $[k_{\min}, k_{\max}]$ where $k_{\min}$ and $k_{\max}$ are chosen such that the average degree is $\langle k \rangle$. Edges are then created via the stub-matching approximation of the configuration model. That is, the degree of each vertex is represented by "half edges" or "edge stubs" and the stubs of the network are connected uniformly at random.

2. The community sizes are drawn from a power law distribution with exponent τ_2 such that the sum of community sizes equals N, the number of nodes in the network. This distribution is truncated to $[s_{\min}, s_{\max}]$ with $k_{\min} < s_{\min} < k_{\max} < s_{\max}$, which allows each node (of any degree) to be included in at least one community.

3. Nodes are assigned to communities, starting from no assignments and proceeding to assign nodes to communities one-by-one. When assigning a node to a community, the assignment is successful only if the community is larger than $(1-\mu)$ times the degree of the node (otherwise, it is impossible to satisfy the mixing parameter condition). If unsuccessful, a randomly selected node is removed from this community. This procedure repeats until all nodes are assigned a community label.

4. The network is repeatedly rewired so that the per-node fraction of edges connected to other communities is approximately equal to μ. Importantly, this rewiring keeps the degree and community size sequences of the network fixed.

If this procedure converges, the network satisfies the conditions imposed by the chosen parameters. In Figure S26, we show an example of two such networks colored by their ground truth communities.

I.2 Our Pruning Method Applied to LFR Networks

We now test our pruning pipeline on LFR benchmark graphs in order to test performance when community sizes vary significantly. As in Figure S26, we generate graphs of $N = 500$ nodes with $\tau_1 = 2.5$ and $\tau_2 = 1.5$ which correspond to the middle of the typical parameter ranges provided in [30] ($2 \leq \tau_1 \leq 3$ and $1 \leq \tau_2 \leq 2$). As in Lancichinetti et al. [30] we also choose $\langle k \rangle = 20$ and sweep across a uniform grid of $\mu \in [0.1, 0.5]$ to include graphs ranging from very strong to weak community structure. At $\mu = 0.1$, approximately 90% of node neighbors are placed within communities. Beyond $\mu = 0.5$, communities are "no longer defined in the strong sense" as nodes have more neighbors in other communities than their own. For each choice of μ, we generate 100 realizations of LFR benchmark graphs. On each of these graphs we run the following four community detection strategies and compare the results.

1. Run the Louvain heuristic 500 times on a uniform grid of resolution parameter choices $\gamma \in [0.5, 2.0]$. This is used as a baseline to compare our pruning method against the modularity maximization heuristic with no post-processing.

2. Run the Louvain heuristic 500 times at the γ value given by evaluating the gamma estimate from Equation S3 on the ground truth community structure. We refer to this as running Louvain "with the ground truth gamma estimate". In practice, without the ground truth one would most likely not know *a priori* which value of γ is "correct", but we will find this to be useful for comparison purposes.

3. Run our modularity pruning pipeline starting from 500 runs of the Louvain heuristic on a uniform grid of $\gamma \in [0.5, 2.0]$.

4. Repeat strategy 3, but with the number of communities K fixed during pruning at the number of communities seen in the ground truth partition. Once again, we note that such a ground truth value is not always (or even typically) known in practice, but we include here for comparison with situations where K might be provided or reasonably estimated.

We evaluate the performance of these strategies by computing the NMI of the returned partitions with the ground truth partition from the LFR algorithm. Here, a value closer to 1.0 indicates better alignment with the ground truth. We show these results in Figure S27. First, we note that the modularity pruning method attains higher alignment with the ground truth and more consistent results than the other methods when stable partitions are found. That is, the lower left plot of Figure S27 shows higher and less variable values for the NMI with ground truth when comparing to the Louvain baselines in the top two plots. However, it's important to note that the lower left plot only includes NMIs from the runs in which our pruning method led to stable partitions (that is, fixed points of the corresponding map) with γ estimates inside the selected range, $[0.5, 2.0]$.

Notably, we observe that the probability of finding a stable partition decreases as the mixing parameter μ increases (i.e. as the strength of the underlying community structure decreases). This suggests that even in this case where the communities are not of roughly equal size (and thus the assumptions of the equivalence do not hold), our notion of stability is a good proxy for the significance or strength of the underlying community structure.

Moreover, we note the variability in the Louvain baselines is relatively large even for small μ. Given this, it may not be sufficient to simply run the iterative schemes of Newman and Pamfil et al. to convergence. Instead, one might need to repeatedly run modularity maximization heuristics at a converged value of γ in order to recover the latent community structure more completely. This may be particularly important when the latent community structure of a network is weak, since the variability in the Louvain baselines are then even further from the ground truth. Indeed, the consideration of many different partitions is precisely the feature of our method that tends to suppress the pseudo-random stochasticity originating in the heuristic nature of common community detection algorithms.

We also note that when we restrict focus to the ground truth number of communities, our modularity pruning method either outperforms or matches the behavior of Louvain running at the ground truth value for γ, even as μ approaches 0.5 and the ground truth community structure becomes weaker. Especially for μ close to 0.5, this implies that our pruning scheme is able to recover the underlying network structure at least as well as one would be able to by running the Louvain heuristic with *a priori* knowledge of a "correct" choice of γ. It is of course rarely the case that one would even have such knowledge, but our procedure performs at least as well without this knowledge, especially if the number of communities in the underlying data set can be estimated.

J Maximum γ Estimates

In this section, we derive maximum possible expected values for the γ estimates for ground truth community assignments in planted partition stochastic block models with equal-sized blocks. We first derive the result for non-degree-corrected planted partition SBMs and then show that the same maximums hold in the degree-corrected case. Finally, we demonstrate that real-world networks have community structure consistent with these derivations, yielding bounds on which values of γ "should" be used in modularity when searching for partitions of K communities.

J.1 Equal Block Size, Non-Degree-Corrected, Planted Partition SBMs

Consider a simple stochastic block model that follows some of the "assumptions of modularity" derived from Newman's equivalence [1] (discussed in section A.5). In particular, consider a planted partition stochastic block model in which all blocks have equal size. Let pairs of nodes within communities be connected with probability p_{in} and pairs of nodes between communities be connected with probability p_{out}. Here, we require that $p_{\text{in}} > p_{\text{out}}$ in order to have assortative SBMs that exhibit community structure (this makes the logic easier, though for this simple SBM the results hold for arbitrary p_{in} and p_{out}).

For simplicity, this SBM is not degree-corrected — we are defining the SBM in terms of p_{in} and p_{out} probabilities rather than ω_{in} and ω_{out} propensities. We further make the simplifying assumption that nodes cannot be connected to themselves; i.e.

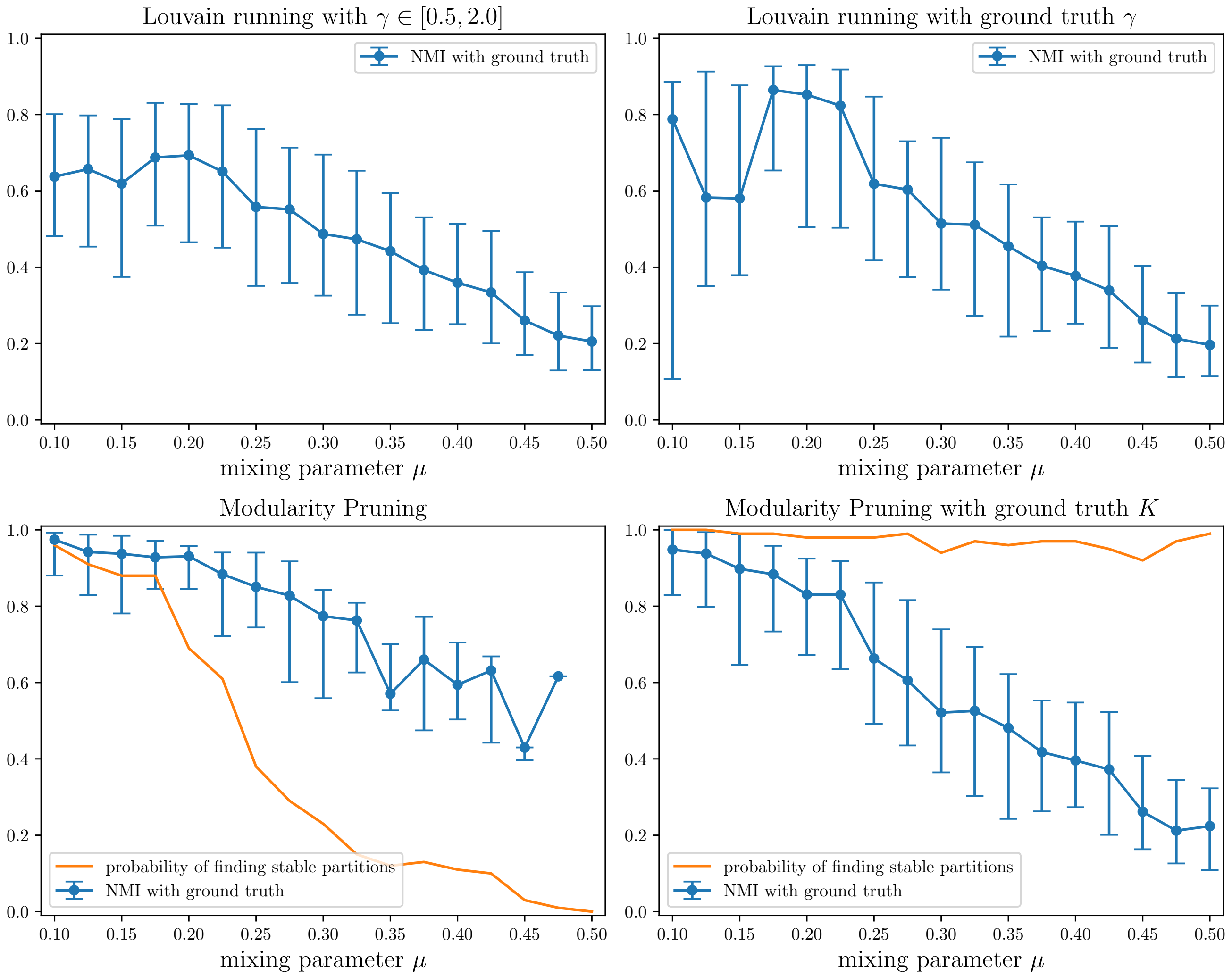

Figure S27. Median NMI between the partitions returned by the four methods described in section I.2 and the ground truth partitions of LFR benchmark networks. Each choice of μ corresponds to 100 realizations of the LFR algorithm. Error bars indicate the 25th and 75th percentiles of the NMIs obtained from running each of the four methods on these realizations. In the top left, we note that the Louvain heuristic is run at 500 different values of γ, and thus there is no basis for selecting any of these output partitions as better than others without performing CHAMP or some similar procedure; as such, we report here the distribution across the NMIs of each Louvain output versus the ground truth. In the top right, where we run Louvain 500 times at the single γ estimate obtained for each LFR ground truth partition, one could select the highest modularity partition from these 500 runs; but since this panel is already assuming information not typically available *a priori*, notably the correct γ estimate for the ground truth, we again plot the full distribution of NMIs across the Louvain outputs for more direct comparison with the results in the top left panel. In the bottom row, plotting the distributions of NMIs obtained for stable partitions identified by our method, the orange lines indicate the empirically observed fraction of the 100 LFR realizations at each μ that led to stable partitions with γ estimates inside the selected range, $[0.5, 2.0]$, for the corresponding pruning method.

self loops cannot exist. We thus generate networks with N nodes, divided into K blocks of size $B = N/K$ nodes each. These K communities are connected according to the $K \times K$ matrix of probabilities

$$\mathbf{P} = \begin{bmatrix} p_{\text{in}} & p_{\text{out}} & \cdots & p_{\text{out}} \\ p_{\text{out}} & p_{\text{in}} & \cdots & p_{\text{out}} \\ \vdots & \vdots & \ddots & \vdots \\ p_{\text{out}} & p_{\text{out}} & \cdots & p_{\text{in}} \end{bmatrix}.$$

We then have the following expected values:

$$\begin{aligned} \text{mean degree } \langle k \rangle &= (B-1)p_{\text{in}} + (K-1)B \cdot p_{\text{out}} \\ \sum_r \kappa_r^2 &= K \cdot \left[B(B-1)p_{\text{in}} + (K-1)B^2 \cdot p_{\text{out}}\right]^2 \\ m_{\text{in}} &= K \cdot \binom{B}{2} \cdot p_{\text{in}} \\ m_{\text{out}} &= \binom{K}{2} \cdot B^2 \cdot p_{\text{out}} \end{aligned}$$

and thus from Equation S4 we obtain that, in expectation,

$$\omega_{\text{in}} = \frac{K(B-1)p_{\text{in}}}{(B-1)p_{\text{in}} + (K-1)B \cdot p_{\text{out}}} \qquad \text{and} \qquad \omega_{\text{out}} = \frac{KB \cdot p_{\text{out}}}{(B-1)p_{\text{in}} + (K-1)B \cdot p_{\text{out}}}. \tag{S25}$$

Hence, our expected "ground truth" γ estimate from Equation S3 is given by

$$\gamma = \frac{K(B-1)p_{\text{in}} - KB \cdot p_{\text{out}}}{(B-1)p_{\text{in}} + (K-1)B \cdot p_{\text{out}}} \cdot \frac{1}{\ln\left(\frac{B-1}{B} \cdot \frac{p_{\text{in}}}{p_{\text{out}}}\right)}$$

and in the limit of large N, this yields

$$\lim_{N\to\infty} \gamma = \frac{K \cdot p_{\text{in}} - K \cdot p_{\text{out}}}{p_{\text{in}} + (K-1) \cdot p_{\text{out}}} \cdot \frac{1}{\ln\left(\frac{p_{\text{in}}}{p_{\text{out}}}\right)} = \frac{K\left(\frac{p_{\text{in}}}{p_{\text{out}}} - 1\right)}{\left(\frac{p_{\text{in}}}{p_{\text{out}}} + (K-1)\right)\ln\left(\frac{p_{\text{in}}}{p_{\text{out}}}\right)}. \tag{S26}$$

Note this limit only involves changing the $(B-1)$ factors to B, so the expected γ estimate above is often close to this limiting value as long as the size B of the blocks is not too small. Note also that, perhaps surprisingly, this value is independent of the expected degree in the network; it only depends on the ratio $p_{\text{in}}/p_{\text{out}}$ and the number of blocks K and is thus directly tied to the strength of the SBM's community structure. (Note we are implicitly assuming that $p_{\text{out}} \neq 0$ here, since this is required to keep the network connected.)

Even more surprisingly, Equation S26 implies that for a given value of K, the possible expected γ estimates are bounded above. This is shown in Figure S28, where we plot the expected γ estimates with respect to $p_{\text{out}}/p_{\text{in}}$ (to keep the ratio bounded within 0 and 1), so smaller values of $p_{\text{out}}/p_{\text{in}}$ actually represent stronger community structure. We numerically evaluate the maxima of this function (for $K > 2$, the maxima appear to be transcendental and thus cannot be written in any particularly nice way) and gathered maximum γ estimates for various K in Table S5.

K	2	3	4	5	6	7	8	9	10
$\gamma_{\max}$	1.0000	1.0926	1.2427	1.4027	1.5640	1.7241	1.8824	2.0388	2.1931

Table S5. Maximum expected ground truth γ estimates, $\gamma_{\max}$, rounded to four decimal places, for the non-degree-corrected planted partition SBM with equal-sized blocks, for $2 \leq K \leq 10$. We note that all of the γ estimates given in Newman's paper [1] on the modularity-SBM equivalence are below the corresponding $\gamma_{\max}$ derived here.

In this way, it is never "correct" to be searching for 5-community partitions beyond $\gamma \gtrsim 1.4$ if we wish to make modularity maximization equivalent to the maximum likelihood fit to the SBM (which is *not* degree-corrected here, and, again, also assumes equal-sized blocks). Indeed, since partitions that exhibit strong community structure are often close to optimal near

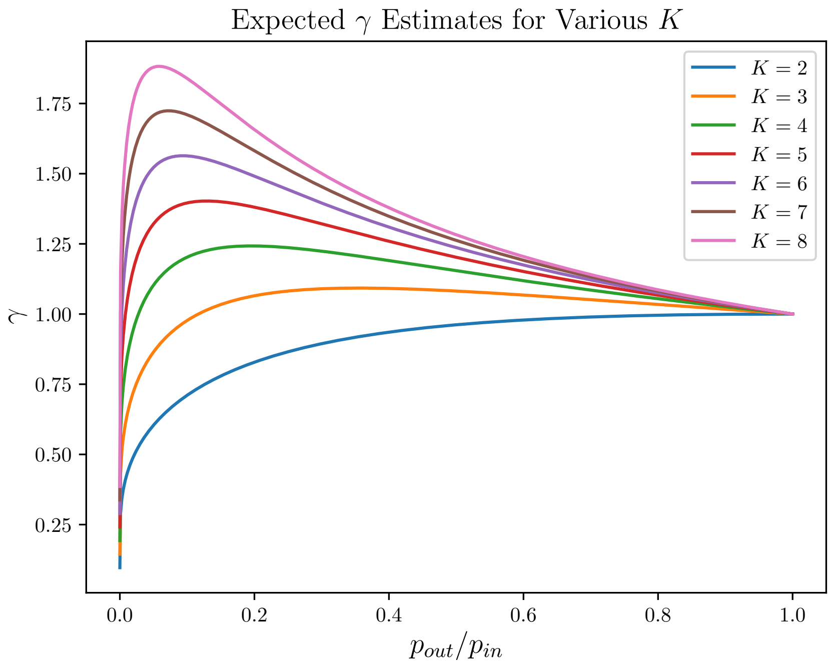

Figure S28. The expected γ estimates for various choices of K in our SBM as $p_{\text{out}}/p_{\text{in}}$ varies.

their γ estimates, this also suggests that one should not be run modularity maximization heuristics above the $\gamma_{\max}$ values associated with the maximum K of interest.

It is important to realize that the derivation of these maximum γ estimates in the present subsection are not immediately applicable in the degree-corrected case. Indeed, we can see immediately from Equation S25 that the expected ω_{in} and ω_{out} estimates are restricted to

$$\omega_{\text{in}} + (K-1)\omega_{\text{out}} = K \tag{S27}$$

for a K-block SBM here. In other words, denoting $\Omega = (\omega_{\text{in}}, \omega_{\text{out}})$, this simplified model restricts the degree-corrected SBM parameters to a line segment in the Ω-plane. We visually demonstrate this phenomenon in Figure S29.

J.2 Degree-Corrected, Planted Partition SBMs

We now show that the same maximum γ estimates hold in the (slightly more general) degree-corrected planted partition SBM with equal-sized blocks. For a fixed number of communities $K \geq 2$, we can rewrite our ω_{in} and ω_{out} estimates from Equation S4 as

$$\omega_{\text{in}} = \frac{4m \cdot m_{\text{in}}}{\sum_r \kappa_r^2} \qquad \text{and} \qquad \omega_{\text{out}} = \frac{4m^2 - 4m \cdot m_{\text{in}}}{4m^2 - \sum_r \kappa_r^2}.$$

Then, all $\kappa_r > 0$ and $\sum_r \kappa_r = 2m$, so the Cauchy-Schwarz inequality yields

$$\begin{aligned} \left(\sum_{r=1}^{K} \kappa_r \cdot 1\right)^2 &\leq \left(\sum_{r=1}^{K} \kappa_r^2\right) \cdot \left(\sum_{r=1}^{K} 1^2\right) \\ \frac{4m^2}{K} &\leq \sum_{r=1}^{K} \kappa_r^2. \end{aligned}$$

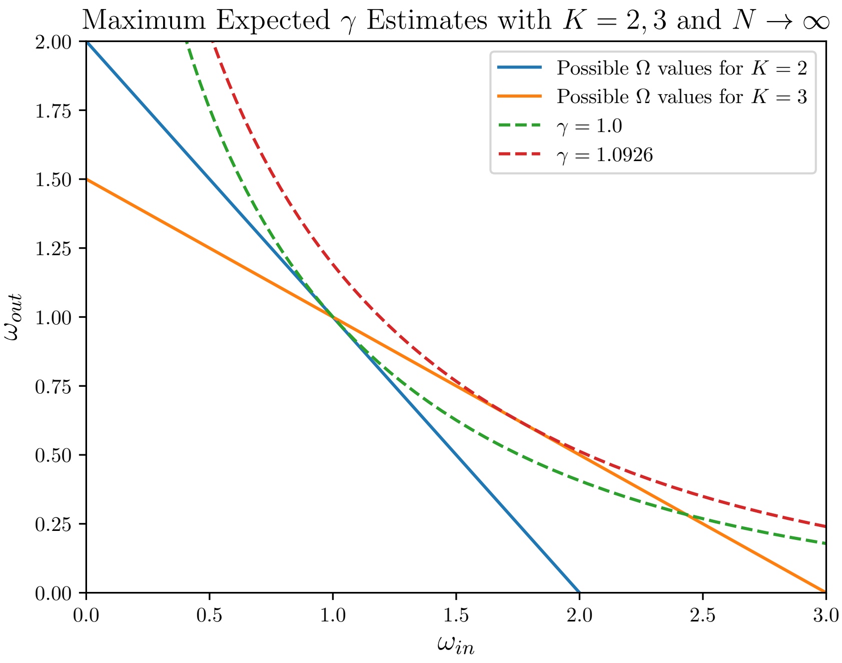

Figure S29. Visualization of possible ω_{in} and ω_{out} values and associated maximum expected $\gamma_{\max}$ values in a (not degree corrected) planted partition SBM with equal-sized blocks, for $K = 2$ and 3.

Moreover, when we have assortative community structure (i.e. $\omega_{\text{in}} > \omega_{\text{out}}$),

$$\begin{aligned}
\omega_{\text{in}} &> \omega_{\text{out}} \\
\frac{4m \cdot m_{\text{in}}}{\sum_r \kappa_r^2} &> \frac{4m^2 - 4m \cdot m_{\text{in}}}{4m^2 - \sum_r \kappa_r^2} \\
(4m \cdot m_{\text{in}}) \left(4m^2 - \sum_r \kappa_r^2\right) &> \left(4m^2 - 4m \cdot m_{\text{in}}\right) \left(\sum_r \kappa_r^2\right) \\
16m^3 \cdot m_{\text{in}} - \cancel{4m \cdot m_{\text{in}} \sum_r \kappa_r^2} &> 4m^2 \sum_r \kappa_r^2 - \cancel{4m \cdot m_{\text{in}} \sum_r \kappa_r^2} \\
4m \cdot m_{\text{in}} &> \sum_r \kappa_r^2 ,
\end{aligned}$$

and thus $\frac{4m^2}{K} \leq \sum_r \kappa_r^2 < 4m \cdot m_{\text{in}}$. Hence,

$$\omega_{\text{in}} + (K-1)\omega_{\text{out}} = \frac{4m \cdot m_{\text{in}}}{\sum_r \kappa_r^2} + (K-1)\frac{4m^2 - 4m \cdot m_{\text{in}}}{4m^2 - \sum_r \kappa_r^2} ,$$

and for these possible $\sum_r \kappa_r$ values,

$$\frac{\partial}{\partial m_{\text{in}}} \left[\omega_{\text{in}} + (K-1)\omega_{\text{out}}\right] = \frac{4m}{\sum_r \kappa_r^2} - (K-1)\frac{4m}{4m^2 - \sum_r \kappa_r^2} < 0 \iff \sum_r \kappa_r^2 > \frac{4m^2}{K} .$$

Finally, this means

$$\begin{aligned}
\omega_{\text{in}} + (K-1)\omega_{\text{out}} &\leq \frac{4m \cdot 0}{\sum_r \kappa_r^2} + (K-1)\frac{4m^2 - 4m \cdot 0}{4m^2 - \sum_r \kappa_r^2} \\
&\leq (K-1)\frac{4m^2}{4m^2 - 4m^2/K} \\
&\leq (K-1)\frac{1}{1 - 1/K} = (K-1)\frac{K}{K-1} = K ,
\end{aligned}$$

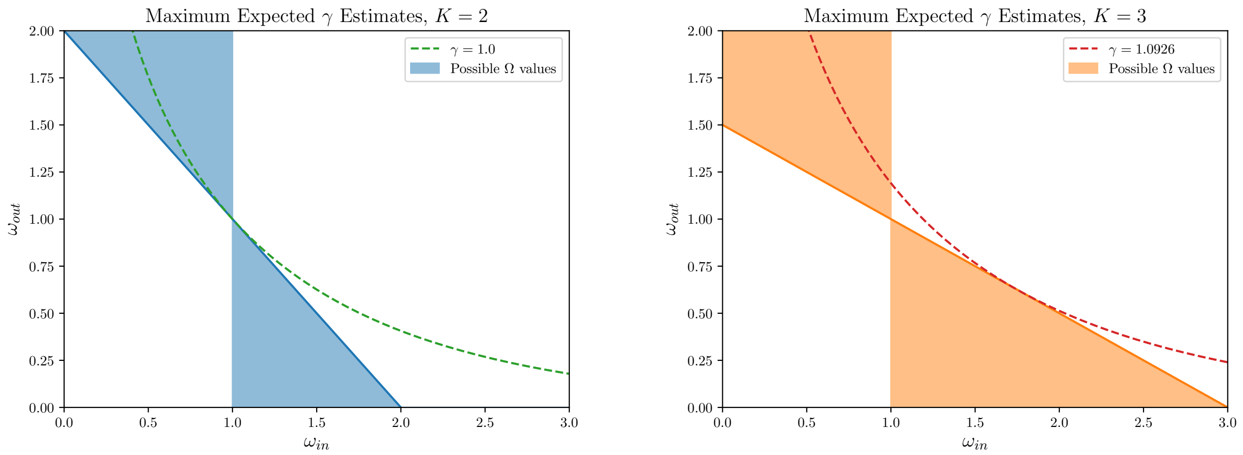

Figure S30. Visualization of possible ω_{in} and ω_{out} values in the degree-corrected SBM with equal-sized blocks for $K = 2$ and 3. Note that the maximum γ estimates from section J.1 hold when $\omega_{\text{in}} > \omega_{\text{out}}$, that is, in the assortative case.

which matches the bound we obtained in Equation S27 for the non-degree-corrected case. We visualize this bound in the $\Omega = (\omega_{\text{in}}, \omega_{\text{out}})$ plane in Figure S30.

J.3 Observed γ Estimates in Real-World Networks

We now compute γ estimates on the giant connected component of a handful of networks from the Stanford Large Network Dataset Collection [32]. Specifically, we consider 16 single-layer social networks ranging from 4k to 82k nodes and 17k to 948k edges:

- the 8 networks from the Gemsec Facebook dataset (gemsec-Facebook),
- the 3 networks from the Gemsec Deezer dataset (gemsec-Deezer),
- the 2 Slashdot social networks from November 2008 and February 2009 (soc-Slashdot0811 and soc-Slashdot0922),
- the (anonymized) social circles from Facebook (ego-Facebook),
- the Who-trusts-whom network of Epinions.com (soc-Epinions1), and
- the Wikipedia who-votes-on-whom network (wiki-Vote).

We ran the Louvain algorithm 1000 times on each network on a uniform grid of $\gamma \in [0, 10]$. We computed γ estimates for each partition and grouped the full collection of γ estimates by the number of communities in the partition, K. The results in Figure S31 are plotted along with the γ_{max} bound derived from Equation S26 and the "average γ estimate", γ_{mean}, obtained from the average value of $\gamma = (\omega_{\text{in}} - \omega_{\text{out}})/(\ln \omega_{\text{in}} - \ln \omega_{\text{out}})$ on the triangle in the $\Omega = (\omega_{\text{in}}, \omega_{\text{out}})$ plane with vertices $(1, 0)$, $(1, 1)$, and $(K, 0)$. (These triangles define the possible Ω estimates for assortative degree-corrected SBMs, as visualized in Figure S30.)

We confirm that all observed γ estimates from these Louvain runs on these networks lie below our γ_{max} values. At the same time, we observe that our γ_{mean} values obtained on the corresponding triangles in the Ω plane appear to only slightly overestimate the trend in K for the median values of the γ estimates obtained in practice. Thus we have *a priori* bounds on the range of resolution parameters that should be used in modularity maximization if a maximum desired number of communities is known or can be estimated. We are hopeful that these values can be used to further guide modularity-based community detection strategies in a less ad hoc way.

J.4 Limiting Behavior of γ_{max}

Even though the values for γ_{max} with $K > 2$ appear to be transcendental, one might wonder if there is a simple linear approximation for the function as K grows large. With the plot in Figure S31 suggesting a possible linear trend for large K, we further plot the function out to $K = 10^6$ in Figure S32.

However, one can simply check a few tangents to the function to see that γ_{max} is not as linear as it may visually appear here. We plot two such examples in Figure S33 to show that the function diverges from local linear approximations relatively quickly. We also plot there the central finite difference approximation to the first derivative, observing that it does not quickly

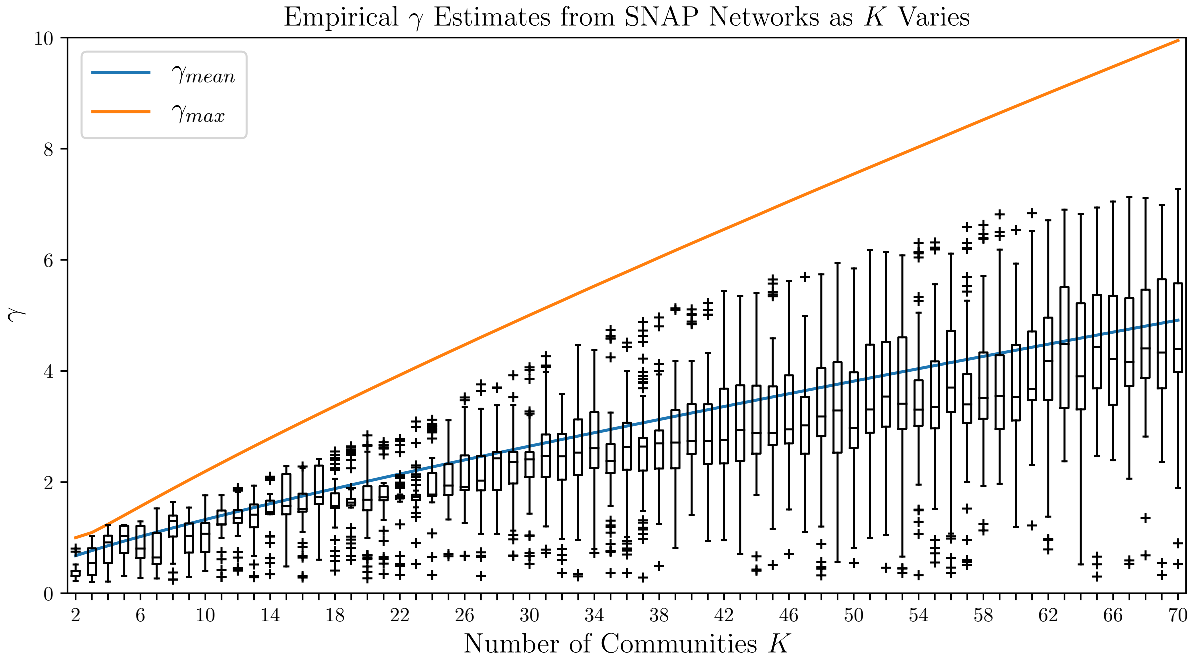

Figure S31. Boxplots of observed γ estimates on 16 social networks from SNAP [32], plotted alongside our γ_{max} bound and γ_{mean} expected average for the degree-corrected planted partition SBM with equal-sized blocks.

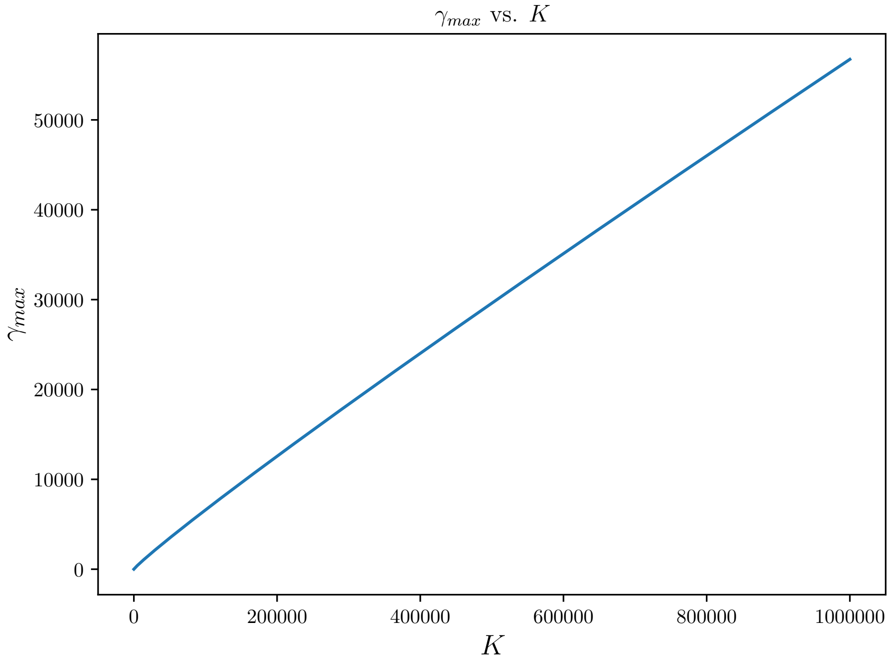

Figure S32. Plot of maximum γ estimates from $K = 2$ to $K = 10^6$.

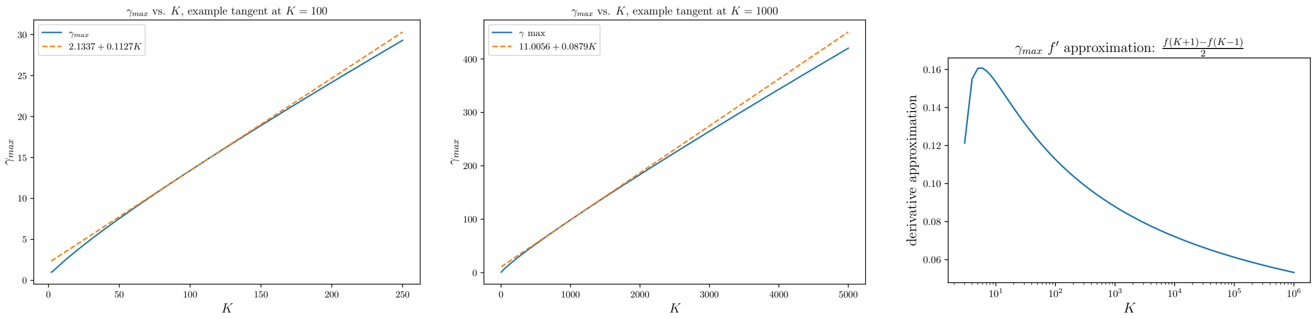

Figure S33. (Left and Center) Two example tangents to $\gamma_{\max}$, showing that the function is not as linear as it may appear at a first glance. (Right) The central finite difference approximation for the first derivative of $\gamma_{\max}(K)$. The lack of a useful long-term trend in the derivative makes local linear approximations diverge.

converge to any value; instead, it appears to be continuing to decrease very slowly with increasing K. There may be more complicated functions that approximate $\gamma_{\max}$ well, but this would be mostly of theoretical interest since fortunately the numerical maximization for computing $\gamma_{\max}(K)$ runs very quickly and seems relatively well behaved in our tests.

K Performance of Our Method

In this section, we analyze some performance aspects of our method. First, we demonstrate that the overhead of our pruning strategy is minimal compared to the time required to obtain the partitions of interest. Then, we investigate the existence of "parameter estimation loops" in networks, which could cause our method to fail to find potentially significant community structure. Importantly, the corresponding parameter estimation techniques proposed by Newman [1] and Pamfil et al. [2] could also have convergence issues if run on a graph exhibiting this phenomenon.

K.1 Overhead of Modularity Pruning

We now show that the runtime of our pruning technique is effectively negligible when compared to the underlying use of the modularity maximization heuristics. First, we generated an Erdős-Rényi random graph with 1000 nodes and 5000 edges. Then, we ran iterations of the Louvain heuristic with this graph on a uniform grid of $\gamma \in [0.0, 2.0]$ and ran the resulting partitions through our pruning pipeline. We show the elapsed runtimes from this test in Figure S34. We see here that the runtime of the pruning procedure is very small compared to the time required to obtain the partitions themselves. Indeed in this example, the step to prune the partitions runs in about 5% of the time required to obtain the partitions from the Louvain heuristic.

Moreover, we note that the majority of our procedure scales with the number of *unique* input partitions. Since we are running on a random graph here, the community structure is very weak (in fact, by definition, any significant community structure in this graph occurs by chance alone). As a result, nearly 80% of the Louvain runs in this experiment return a new, unique partition that was not found in earlier runs. In more practical, "real-world" scenarios we would expect the modularity maximization heuristic to return unique partitions less frequently and thus our procedure to run more quickly.

We additionally note that the implementation of Louvain [8] used here was written in C++ over the course of many years, using igraph [7], a networks library written in C "with an emphasis on efficiency". On the other hand, our procedure is mostly implemented in Python and we believe that its running times could be improved if needed.

Finally, recall that most of the overhead in our pruning procedure comes from CHAMP's partition-to-halfspace calculation and thus scales linearly with the size of the graph. In contrast, the Louvain heuristic is believed to run typically in time $O(N \log N)$ on sparse graphs with N nodes [16]. Thus, the relative overhead of our modularity pruning compared to modularity maximization will decrease (albeit slowly) as the size of the graph grows. Indeed, this matches the behavior we have seen on graphs derived from very large social networks data sets.

K.2 Potential Convergence Issues: Parameter Estimation Loops

Recall that Newman's iterative parameter estimation involves maximizing modularity at a resolution parameter value γ and then repeatedly updating this choice via Equation S3. This is repeated until convergence, but the best guarantee to date is Newman's note in [1] that

> "This procedure should converge to the correct value of γ (and the correct community structure) for sufficiently dense networks that actually are generated from the planted partition model. [...] In practice, we have found that it converges very quickly."

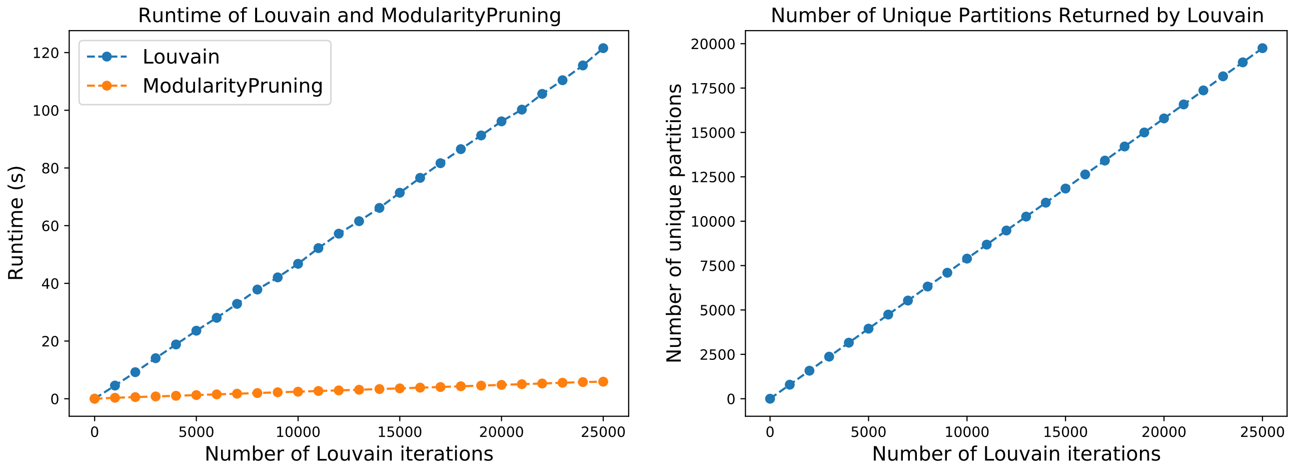

Figure S34. Left: The runtime of the Louvain heuristic and our pruning pipeline as the number of runs of Louvain increases. Right: A comparison between the number of runs of Louvain and the number of unique partitions returned by the algorithm. This test was run on a machine with an i7-9700K CPU and 16 GB of DDR4 RAM running at 3200 MHz. The iterations of Louvain were run in parallel on all 8 cores simultaneously.

However, there does not appear to be any general guarantee for how quickly this procedure would converge or when convergence can be guaranteed. Consider the following situation.

1. A modularity maximization procedure with $\gamma = \gamma_1$ returns a partition σ_1. This partition has gamma estimate γ_2.
2. The same modularity maximization procedure with $\gamma = \gamma_2$ returns a partition σ_2. This partition has gamma estimate γ_1.

In this case, Newman's iterative scheme would repeatedly iterate between γ_1 and γ_2 and thus never converge. Similarly, in the case where the true modularity maximizing partitions are σ_1 and σ_2, our procedure would behave the same way. That is, in this case the corresponding map has a periodic orbit of period 2. We call this phenomenon where the iteration endlessly returns to a particular value of γ a "parameter estimation loop".

K.3 Parameter Estimation Loops Exist

It is not particularly difficult to find a pair of partitions whose γ estimates lie in each others' domains of (modularity) optimality. If a modularity maximization scheme were to return these two partitions in their domains of optimality, iterative parameter estimation procedures would repeatedly swap between the two partitions and never terminate. We show such an example in Figure S35. However, it is important to note that we have been unable to find such an example in which both partitions satisfy $\omega_{\text{in}} > \omega_{\text{out}}$. In this sense, we have never observed this phenomenon when the partitions both represent assortative community structure. (Indeed, Newman's duality shows that the stochastic block model inference is actually equivalent to modularity *minimization* when $\omega_{\text{in}} < \omega_{\text{out}}$. Hence, the period 2 orbit in the iterative scheme here might be attributed to a breakdown in the equivalence itself.)

K.4 Attempts to Find Parameter Estimation Loops with Modularity Pruning

We attempted to generate networks with parameter estimation loops in partitions returned by modularity maximization heuristics in the following way.

1. Generate an Erdős-Rényi random graph with number of nodes $n \in [10, 100]$ and average degree $\langle k \rangle \in [6, 20]$. We rejected all realizations in which the graph was disconnected.
2. Run the Louvain heuristic 1000 times on a uniform grid of $\gamma \in [0, 3]$. This maximizes modularity and is thus expected to return assortative partitions that satisfy $\omega_{\text{in}} > \omega_{\text{out}}$.
3. Use the resulting partitions in our modularity pruning technique and determine if any partitions from the CHAMP set of somewhere dominant partitions belonged to a parameter estimation loop.

We repeated this more than 1 million times, but were unable to find *any* occurrences of this looping issue. From this, we expect that this phenomenom is either incredibly unlikely in practice or perhaps even impossible when considering assortative

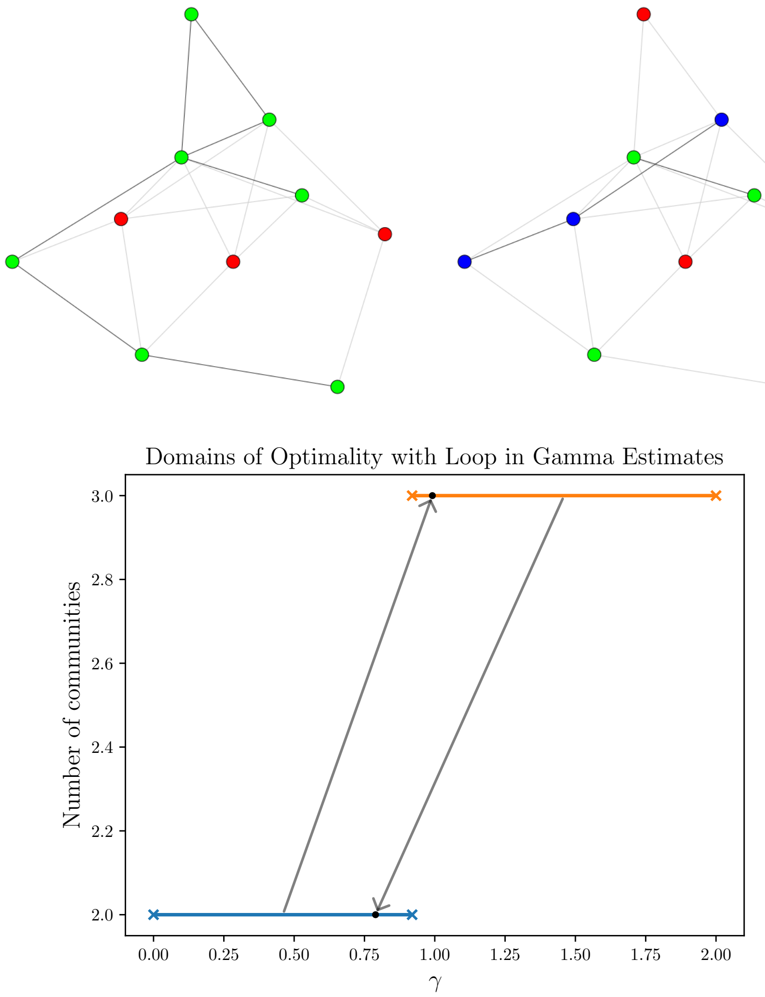

Figure S35. A pair of 2-community and 3-community partitions of a network whose γ estimates lie in each other's domains of optimality. Top: Force-directed layouts of the two partitions. Bottom: The partitions' domains of (modularity) optimality and their corresponding γ estimates.

partitions (i.e. $\omega_{\text{in}} > \omega_{\text{out}}$). If such periodic orbits (beyond simple fixed points) of the map are indeed impossible, our pruning framework and any deterministic modularity maximization algorithm used in tandem with Newman's iterative parameter estimation would never encounter this type of convergence issue.

In Figure S36, we show a few representative examples of the domains of optimality and gamma estimates from these realizations. Note that the iteration in our pruning framework typically converges to a central choice of γ and never enters a loop (except for stable partitions which correspond to self-loops). In practice, we have observed similar behavior on real-world networks where we typically find a small number of stable partitions and parameter estimation on the somewhere dominant partitions converges quickly.

References

1. Newman, M. E. J. Equivalence between modularity optimization and maximum likelihood methods for community detection. *Phys. Rev. E* **94**, 052315, DOI: 10.1103/PhysRevE.94.052315 (2016).

2. Pamfil, A. R., Howison, S. D., Lambiotte, R. & Porter, M. A. Relating Modularity Maximization and Stochastic Block Models in Multilayer Networks. *SIAM J. on Math. Data Sci.* **1**, 667–698, DOI: 10.1137/18M1231304 (2019).

3. Weir, W. H., Emmons, S., Gibson, R., Taylor, D. & Mucha, P. J. Post-Processing Partitions to Identify Domains of Modularity Optimization. *Algorithms* **10**, 93, DOI: 10.3390/a10030093 (2017).

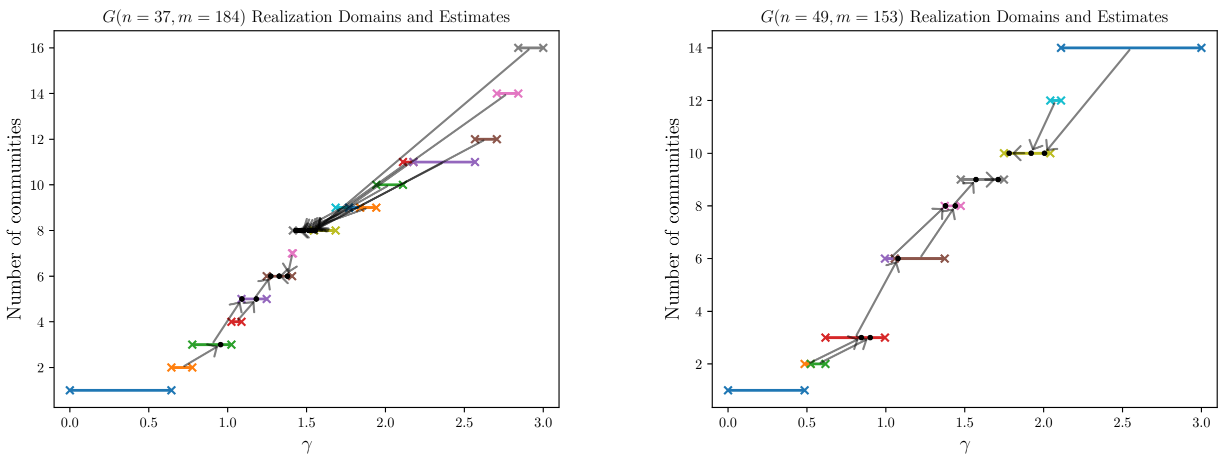

Figure S36. Two representative examples of the behavior in our attempts to find parameter estimation loops with our pruning scheme. Domains of optimality from the CHAMP algorithm are shown as horizontal segments in γ space with their position along the y axis determined by the number of communities in the corresponding partition. Each domain of optimality is annotated with an arrow that points to the partition's γ estimate.

4. Weir, W. H., Gibson, R. & Mucha, P. J. *CHAMP package: Convex Hull of Admissible Modularity Partitions in Python and MATLAB* (2017). https://github.com/wweir827/CHAMP.

5. Zachary, W. W. An Information Flow Model for Conflict and Fission in Small Groups. *J. Anthropol. Res.* **33**, 452–473, DOI: 10.1086/jar.33.4.3629752 (1977).

6. Emmanuel Lazega. *The Collegial Phenomenon: The Social Mechanisms of Cooperation Among Peers in a Corporate Law Partnership* (Oxford University Press, 2001).

7. Gabor Csardi & Tamas Nepusz. The igraph software package for complex network research. *InterJournal, Complex Syst.* **1695**, 1–9 (2006). http://igraph.org.

8. Traag, V. *Implementation of the Louvain algorithm for community detection with various methods for use with igraph in python.* (2019). https://github.com/vtraag/louvain-igraph.

9. Newman, M. E. J. & Girvan, M. Finding and evaluating community structure in networks. *Phys. Rev. E* **69**, DOI: 10.1103/physreve.69.026113 (2004).

10. Reichardt, J. & Bornholdt, S. Statistical mechanics of community detection. *Phys. Rev. E* **74**, DOI: 10.1103/physreve.74.016110 (2006).

11. Arenas, A., Fernandez, A. & Gomez, S. Analysis of the structure of complex networks at different resolution levels. *New J. Phys.* **10**, 053039, DOI: 10.1088/1367-2630/10/5/053039 (2008).

12. Fortunato, S. & Barthélemy, M. Resolution limit in community detection. *Proc. Natl. Acad. Sci.* **104**, 36–41, DOI: 10.1073/pnas.0605965104 (2007).

13. Brandes, U. *et al.* *Maximizing Modularity is hard* (2006). _eprint: physics/0608255.

14. Brandes, U. *et al.* On Finding Graph Clusterings with Maximum Modularity. In Brandstädt, A., Kratsch, D. & Müller, H. (eds.) *Graph-Theoretic Concepts in Computer Science*, Lecture Notes in Computer Science, 121–132 (Springer Berlin Heidelberg, 2007).

15. Dinh, T. N., Li, X. & Thai, M. T. Network Clustering via Maximizing Modularity: Approximation Algorithms and Theoretical Limits. In *2015 IEEE International Conference on Data Mining*, 101–110, DOI: 10.1109/ICDM.2015.139 (2015).

16. Blondel, V. D., Guillaume, J.-L., Lambiotte, R. & Lefebvre, E. Fast unfolding of communities in large networks. *J. Stat. Mech. Theory Exp.* **2008**, P10008, DOI: 10.1088/1742-5468/2008/10/P10008 (2008).

17. Traag, V. A., Waltman, L. & van Eck, N. J. From Louvain to Leiden: guaranteeing well-connected communities. *Sci. Reports* **9**, DOI: 10.1038/s41598-019-41695-z (2019).

18. Karrer, B. & Newman, M. E. J. Stochastic blockmodels and community structure in networks. *Phys. Rev. E* **83**, DOI: 10.1103/PhysRevE.83.016107 (2011).

19. Zhang, L. & Peixoto, T. P. Statistical inference of assortative community structures. *Phys. Rev. Res.* **2**, 043271, DOI: 10.1103/PhysRevResearch.2.043271 (2020).

20. Peixoto, T. P. *Bayesian Stochastic Blockmodeling*, 289–332 (in Advances in Network Clustering and Blockmodeling, John Wiley & Sons, Ltd, 2019).

21. Decelle, A., Krzakala, F., Moore, C. & Zdeborová, L. Inference and Phase Transitions in the Detection of Modules in Sparse Networks. *Phys. Rev. Lett.* **107**, 065701, DOI: 10.1103/PhysRevLett.107.065701 (2011).

22. Mucha, P. J., Richardson, T., Macon, K., Porter, M. A. & Onnela, J.-P. Community Structure in Time-Dependent, Multiscale, and Multiplex Networks. *Science* **328**, 876–878, DOI: 10.1126/science.1184819 (2010).

23. Bazzi, M. *et al.* Community Detection in Temporal Multilayer Networks, with an Application to Correlation Networks. *Multiscale Model. & Simul.* **14**, 1–41, DOI: 10.1137/15M1009615 (2016).

24. Kivelä, M. *et al.* Multilayer Networks. *J. Complex Networks* **2**, 203–271, DOI: 10.1093/comnet/cnu016 (2014).

25. Lomi, A., Robins, G. & Tranmer, M. Introduction to multilevel social networks. *Soc. Networks* **44**, 266–268, DOI: 10.1016/j.socnet.2015.10.006 (2016).

26. Lucas G. S. Jeub, Marya Bazzi, Inderjit S. Jutla & Peter J. Mucha. A generalized Louvain method for community detection implemented in MATLAB (2011–2019). http://netwiki.amath.unc.edu/GenLouvain, https://github.com/GenLouvain.

27. Barber, C. B., Dobkin, D. P., Dobkin, D. P. & Huhdanpaa, H. The Quickhull Algorithm for Convex Hulls. *ACM Trans. Math. Softw.* **22**, 469–483, DOI: 10.1145/235815.235821 (1996).

28. Ghasemian, A., Zhang, P., Clauset, A., Moore, C. & Peel, L. Detectability Thresholds and Optimal Algorithms for Community Structure in Dynamic Networks. *Phys. Rev. X* **6**, 031005, DOI: 10.1103/PhysRevX.6.031005 (2016).

29. Pamfil, R. Itermodmax (2019). https://github.com/roxpamfil/IterModMax.

30. Lancichinetti, A., Fortunato, S. & Radicchi, F. Benchmark graphs for testing community detection algorithms. *Phys. Rev. E* **78**, 046110, DOI: 10.1103/PhysRevE.78.046110 (2008).

31. Hagberg, A. A., Schult, D. A. & Swart, P. J. Exploring Network Structure, Dynamics, and Function using NetworkX. In Varoquaux, G., Vaught, T. & Millman, J. (eds.) *Proceedings of the 7th Python in Science Conference*, 11 – 15 (2008).

32. Leskovec, J. & Krevl, A. *SNAP Datasets: Stanford Large Network Dataset Collection* (2014). http://snap.stanford.edu/data.

Acknowledgements

We are grateful to Zach Boyd, Jim Moody, Roxana Pamfil, Mason Porter, Dane Taylor and William Weir for helpful conversations. We are additionally grateful to William Weir for his contributions to the CHAMP package, which helped make this work possible. This work was supported by the National Science Foundation (BCS-2140024, in collaboration with BCS-2024271) and the James S. McDonnell Foundation (21st Century Science Initiative - Complex Systems Scholar Award grant # 220020315). Additional support was provided by the Army Research Office (MURI award W911NF-18-1-0244). The content is solely the responsibility of the authors and does not necessarily represent the official views of any agency supporting this research.
